# The mitochondrial genomes of *Ancylostoma caninum *and *Bunostomum phlebotomum *– two hookworms of animal health and zoonotic importance

**DOI:** 10.1186/1471-2164-10-79

**Published:** 2009-02-11

**Authors:** Aaron R Jex, Andrea Waeschenbach, Min Hu, Jan A van Wyk, Ian Beveridge, D Timothy J Littlewood, Robin B Gasser

**Affiliations:** 1 Department of Veterinary Science, The University of Melbourne, 250 Princes Highway, Werribee, Victoria 3030, Australia; 2 Department of Zoology, The Natural History Museum, Cromwell Road, London, UK; 3 Department of Veterinary Tropical Diseases, Faculty of Veterinary Science, University of Pretoria, Private Bag X04, 0110 Onderstepoort, South Africa

## Abstract

**Background:**

Hookworms are blood-feeding nematodes that parasitize the small intestines of many mammals, including humans and cattle. These nematodes are of major socioeconomic importance and cause disease, mainly as a consequence of anaemia (particularly in children or young animals), resulting in impaired development and sometimes deaths. Studying genetic variability within and among hookworm populations is central to addressing epidemiological and ecological questions, thus assisting in the control of hookworm disease. Mitochondrial (mt) genes are known to provide useful population markers for hookworms, but mt genome sequence data are scant.

**Results:**

The present study characterizes the complete mt genomes of two species of hookworm, *Ancylostoma caninum *(from dogs) and *Bunostomum phlebotomum *(from cattle), each sequenced (by 454 technology or primer-walking), following long-PCR amplification from genomic DNA (~20–40 ng) isolated from individual adult worms. These mt genomes were 13717 bp and 13790 bp in size, respectively, and each contained 12 protein coding, 22 transfer RNA and 2 ribosomal RNA genes, typical for other secernentean nematodes. In addition, phylogenetic analysis (by Bayesian inference and maximum likelihood) of concatenated mt protein sequence data sets for 12 nematodes (including *Ancylostoma caninum *and *Bunostomum phlebotomum*), representing the Ascaridida, Spirurida and Strongylida, was conducted. The analysis yielded maximum statistical support for the formation of monophyletic clades for each recognized nematode order assessed, except for the Rhabditida.

**Conclusion:**

The mt genomes characterized herein represent a rich source of population genetic markers for epidemiological and ecological studies. The strong statistical support for the construction of phylogenetic clades and consistency between the two different tree-building methods employed indicate the value of using whole mt genome data sets for systematic studies of nematodes. The grouping of the Spirurida and Ascaridida to the exclusion of the Strongylida was not supported in the present analysis, a finding which conflicts with the current evolutionary hypothesis for the Nematoda based on nuclear ribosomal gene data.

## Background

Hookworms (Nematoda: Strongylida: Ancylostomatoidea) are blood-feeding nematodes that inhabit the small intestines of their mammalian host. Species of *Ancylostoma*, *Necator*, *Bunostomum *and *Globocephalus*, for instance, are of major human or animal health significance in various countries [1-6]. The infective, third-stage larvae (L3) can be ingested or penetrate the skin of the host and migrate *via *the circulatory system and the lungs to finally reside, as dioecious adults, usually in the duodenum. The adults attach *via *their buccal capsule to the intestinal mucosa, rupture capillaries and feed on blood. The pathogenesis of hookworm disease in humans and other animals is mainly a consequence of the blood loss, which occurs during parasite attachment and feeding in the intestine. Cutaneous infection can occur and is often associated with inflammatory/immune responses and painful, eruptive lesions during the migration of larvae through the skin [7,8].

Current estimates indicate that more than 740 million people are infected with the hookworms *Ancylostoma duodenale *and *Necator americanus *[9], and ~80 million are severely clinically affected by hookworm disease [10]. In a large number of developing countries, hookworms are a leading cause of iron deficiency anaemia, which, in heavy infections, can cause physical and mental retardation and deaths in children as well as adverse maternal-foetal outcomes [10,11]. Although there is considerably less information on the prevalence and geographical distribution of hookworms of animals [7,12-15], these parasites are also clinically important in dogs (*Ancylostoma braziliense*, *Ancylostoma caninum*, *Ancylostoma ceylanicum *and *Uncinaria stenocephala*), cats (*Ancylostoma tubaeforme*), ruminants (*Bunostomum phlebotomum*, *Bunostomum trigonocephalum *and *Gaigeria pachyscelis*), pigs (e.g., *Globocephalus urosubulatus*) and other hosts [16]. Hookworms were originally thought to be host-specific [17,18]; however, the canine hookworm, *Ancylostoma caninum*, for example, can infect humans and cause dermatitis and eosinophilic enteritis [19], and some hookworm species, such as the bovine hookworm, *Bunostomum phlebotomum*, have been linked to cutaneous lesions in humans [20]. Significant genetic variation has been described among individuals of *Ancylostoma caninum *from dogs in Australia [21]. Such variation might reflect differences in host specificity, infectivity and/or pathogenicity among individual nematodes within a population or, in some cases, might be indicative of speciation events, as has been hypothesized previously for human hookworms [21,22]. Presently, there are no published studies of genetic variation within and among populations of *Bunostomum phlebotomum *and no molecular data are publicly available for this species.

The ability to accurately identify hookworms to species and to assess genetic variability in hookworm populations is central to studying their epidemiology as well as to diagnosis and control. Sequences of the first and second internal transcribed spacers (ITS-1 and ITS-2) of nuclear ribosomal DNA (rDNA) [23-25] and of cAMP-dependent protein kinase [26] have been utilized to identify and differentiate hookworm species. However, the ITS-1 and ITS-2 regions do not usually display sufficient within-species sequence variability to enable the study of the genetic structuring within and among hookworm populations [24]. In contrast, mitochondrial (mt) genomes have been shown to contain useful genetic markers for studying the population structures of hookworm species [27-31], because of their rapid mutation rates and apparent maternal inheritance [32-34]. Although the protein-coding mt gene cytochrome *c *oxidase subunit 1 (*cox1*) is applicable to population studies of a range of invertebrates, including parasitic platyhelminths [35,36] and some nematodes [37,38], there are still limited sequence data for *cox1 *and other mt genes of hookworms, and limited published information is available on sequence heterogeneity therein. Building on advances in long polymerase chain reaction (PCR)-based mt genome sequencing [39-41], the present study determined the sequences and structures of the two mt genomes from an individual of *Ancylostoma caninum *(from a dog) from Australia and a specimen of *Bunostomum phlebotomum *(from a calf) from South Africa. The sequences derived for the mt genomes of these two hookworms were compared in detail with mt genomic data available for the predominant hookworms of humans, *Ancylostoma duodenale *and *Necator americanus *[42], as well as those available for other selected species belonging to the orders Strongylida [41], Ascaridida [43-45] and Spirurida [46-48].

## Results and Discussion

### Mitochondrial genome features, characteristics and gene organization

**Figure 1:**
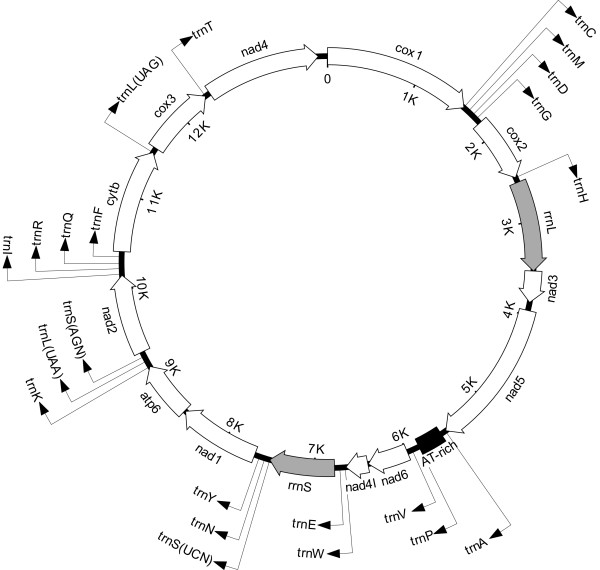
**A representation of the circular mt genomes of *Ancylostoma caninum *(13717 bp) and *Bunostomum phlebotomum *(13790 bp) (GenBank accession numbers **FJ483518**and **FJ483517, **respectively)**. All 12 protein-coding genes and the large and small ribosomal RNA genes are indicated. Each tRNA gene is identified by its single letter amino acid code, according to the international union of pure and applied chemistry (IUPAC) code. The two leucine and the two serine tRNA genes are differentiated by their respective anti-codons (in brackets). The direction of transcription is indicated by an arrow. The circular map has been drawn approximately to scale; "K" indicates sequence length in 'thousands of base pairs' from the first nucleotide position of the cytochrome *c *oxidase subunit 1 (*cox1*) gene.

The circular mt genomes of *Ancylostoma caninum *and *Bunostomum phlebotomum*, sequenced from single adult worms, were 13717 and 13790 bp in size, respectively (Figure 1). Each genome contained 36 genes, including 12 protein coding genes (adenosine triphosphatase subunit 6 [*atp6*], the cytochrome *c *oxidase subunits [*cox1*-*3*], cytochrome *b *[*cytb*], and the nicotinamide dehydrogenase subunits [*nad1-6 *and *nad4l*]), 22 transfer RNA (tRNA) genes and 2 ribosomal RNA genes (small [*rrn*S] and large [*rrn*L] subunits), and was consistent with gene arrangement 2 (GA2) [49]. This arrangement is characteristic for the mt genomes of all members of the Strongylida and Ascaridida, as well as the free-living nematode *Caenorhabditis elegans *(Rhabditida), but not for *Strongyloides stercoralis *(Rhabditida) [41-49]. In accordance with other species of Strongylida for which complete mt genome sequences are available [41,42], the AT-rich regions for both *Ancylostoma caninum *and *Bunostomum phlebotomum *were located between the genes *nad5 *and *nad6*, flanked at the 5'-end by the tRNA gene for alanine, and at the 3'-end by the tRNA genes for proline and alanine.

Each protein-coding gene for each of the two species had an open reading frame (ORF), and all genes were located on the same strand and transcribed in the same direction (5' to 3'), consistent with the known mt genomes of secernentean nematodes [37]. The nucleotide usages (coding strand) of A, C, G and T in each mt genome were 29.0%, 6.5%, 16.1% and 48.5%, respectively, for *Ancylostoma caninum *(Table 1) and 26.9%, 6.2%, 16.7% and 50.1%, respectively, for *Bunostomum phlebotomum *(Table 1), with overall A+T contents of 77.5% and 77.0%, respectively. The A+T content of protein coding genes ranged from 70.9% (*cox1*) to 81.3% (*nad6*) for *Ancylostoma caninum*, and from 70.4% (*cox1*) to 82.6% (*nad3*) for *Bunostomum phlebotomum*. The A+T content for *rrn*S, *rrn*L (= ribosomal RNA genes) and the AT-rich region were 78.1%, 80.9% and 90.1%, respectively, for *Ancylostoma caninum*, and 75.2%, 82.4% and 88.0%, respectively, for *Bunostomum phlebotomum*. For the mt genome of *Ancylostoma caninum*, codon usage in individual protein coding genes (n = 12) ranged from 0% for CGC (arginine) and CCC (proline) to 15.7% for TTT (phenylalanine). For the mt genome of *Bunostomum phlebotomum*, codon usage ranged from 0% for CGC (arginine), CAC (histidine), CTC (leucine), CCC (proline), TCC (serine) and GTC (valine) to 15.0% for TTT (phenylalanine). For both species, individual tRNA structures were consistent with those predicted previously for hookworms and other secernentean nematodes [37,42,45,50,51]. All tRNA genes, except *trn*S(AGN) and *trn*S(UCN), had a predicted secondary structure containing a TV-replacement loop instead of the TψC arm and loop (not shown). The predicted secondary structure of each of the two serine tRNAs contained the TψC arm and loop but lacked the DHU loop. The genes *rrn*S and *rrn*L were 694 bp and 935 bp in length, respectively; the predicted secondary structures for the ribosomal RNA gene subunits for *Ancylostoma caninum *and *Bunostomum phlebotomum *(not shown) were similar to those of *Necator americanus *and *Ancylostoma duodenale *[42], which is also supported by the high nucleotide sequence similarity in the mt genes among these four hookworms (see Tables 2 and 3).

**Table 1:** Nucleotide (nt) composition (%) and A+T contents (%) of the 12 mitochondrial protein coding genes.

	*Ancylostoma caninum*						*Bunostomum phlebotomum*					
**Gene or region**	**Length (nt)**	**A**	**C**	**G**	**T**	**A+T**	**Length (nt)**	**A**	**C**	**G**	**T**	**A+T**

*atp6*	600	29.0	6.0	16.5	48.5	77.5	600	25.7	5.2	18.0	51.2	76.8
*cox1*	1578	25.3	9.6	19.5	45.6	70.9	1578	25.2	9.6	19.5	45.7	70.9
*cox2*	696	29.0	7.5	18.5	45.0	73.4	696	26.0	7.5	19.0	47.6	73.6
*cox3*	766	23.0	7.6	18.7	50.8	73.8	766	24.2	7.0	17.6	51.2	75.3
*cytb*	1113	26.3	7.6	17.6	48.4	74.8	1113	24.6	7.4	17.9	50.1	74.8
*nad1*	870	23.9	7.5	18.4	50.2	74.1	870	22.2	7.4	20.6	49.9	72.1
*nad2*	846	27.3	5.1	14.4	53.2	80.5	846	24.0	4.3	15.0	56.7	80.7
*nad3*	336	26.8	2.4	17.3	53.6	80.4	336	27.1	2.4	14.9	55.6	82.7
*nad4*	1230	27.6	7.1	13.5	51.8	79.4	1230	24.5	6.6	13.5	55.4	79.9
*nad4L*	234	26.9	3.0	16.2	53.9	80.8	234	24.4	3.4	17.5	54.7	79.1
*nad5*	1582	26.6	5.3	16.6	51.5	78.1	1582	26.2	4.9	16.7	52.2	78.4
*nad6*	432	25.7	3.2	15.5	55.6	81.3	432	20.6	4.4	17.8	57.2	77.8
*rrnL*	963	36.2	5.9	13.2	44.7	80.9	963	34.7	5.6	12.0	47.6	82.3
*rrnS*	694	37.0	6.8	15.1	41.1	78.1	694	34.0	7.5	17.3	41.2	75.2
AT-rich	272	44.1	6.3	2.9	46.7	90.1	234	40.6	4.7	7.3	47.4	88.0
Genome	13735	28.9	6.4	16.1	48.5	77.4	13790	26.9	6.2	16.7	50.1	77.0

Large and small ribosomal RNA subunits, AT-rich regions and complete mitochondrial genomes of *Ancylostoma caninum *and *Bunostomum phlebotomum*.

**Table 2:** Pairwise comparison (%) of the amino acid sequences inferred for each of the mitochondrial protein coding genes.

**Protein or gene**	***Bp***	***Ad***	***Na***	***Hc***	***Ans***	***Ass***	***Tc***	***Bm***	***Di***	***Ov***
ATP6	85.9	98.4	89.4	82.4	76.3	77.3	76.8	19.8	22.2	21.2
COX1	97.3	99.2	97.1	94.4	89.5	90.4	91.2	50.7	50.9	51.4
COX2	89.6	96.1	92.6	88.3	83.1	83.6	84.4	41.0	42.3	41.0
COX3	96.0	99.2	95.2	91.0	81.5	81.9	84.7	33.9	33.5	32.0
CYTB	87.0	98.1	85.9	81.6	70.8	74.5	73.5	51.0	48.9	50.8
NAD1	84.4	95.1	87.9	75.5	70.0	71.7	70.8	46.8	50.6	49.1
NAD2	72.5	90.0	77.9	51.6	48.9	46.5	51.2	33.3	28.7	32.0
NAD3	81.9	96.3	81.0	69.3	67.5	68.4	66.6	35.7	35.7	35.7
NAD4	79.2	95.1	85.8	73.1	64.3	63.5	63.0	45.2	44.9	45.8
NAD4L	93.5	100.0	93.5	69.6	75.3	72.7	68.8	35.0	29.1	37.5
NAD5	78.5	94.3	83.6	71.1	66.0	63.4	65.5	38.9	37.4	39.3
NAD6	67.3	88.8	70.8	51.3	59.0	56.2	51.3	26.4	26.4	27.1
*rrn*L	83.4	91.6	82.5	75.4	70.5	70.0	66.4	62.6	61.3	60.4
*rrn*S	86.8	94.9	86.5	78.7	73.0	73.3	70.9	60.6	60.6	59.4

In addition: nucleotide sequences for each of the ribosomal RNA genes of *Ancylostoma caninum *(*Ac*) and *Bunostomum phlebotum *(*Bp*) with those published for species of Strongylida [*Ancylostoma duodenale *(*Ad*), *Necator americanus *(*Na*) and *Haemonchus contortus *(*Hc*)], species of Ascaridida [*Anisakis simplex *(*Ans*), *Ascaris suum *(*Ass*) and *Toxocara canis *(*Tc*)] and species of Spirurida [*Brugia malayi *(*Bm*), *Dirofilaria immitis *(*Di*) and *Onchocerca volvulus *(*Ov*)].

**Table 3:** Pairwise comparison (%) of the amino acid sequences inferred for each of the mitochondrial protein coding genes and nucleotide sequences for each of the ribosomal RNA genes of *Ancylostoma caninum *(*Ac*) and *Bunostomum phlebotum *(*Bp*).

**Protein or gene**	***Ac***	***Ad***	***Na***	***Hc***	***Ans***	***Ass***	***Tc***	***Bm***	***Di***	***Ov***
ATP6	85.9	85.4	86.9	78.8	73.3	74.3	74.8	19.8	21.7	21.2
COX1	97.3	96.9	97.3	94.2	89.7	90.8	91.6	50.7	50.9	51.4
COX2	89.6	88.3	92.2	87.4	79.7	82.7	81.8	41.4	41.8	40.5
COX3	96.0	95.6	94.5	91.8	80.7	82.3	83.5	33.2	33.2	32.0
CYTB	87.0	87.0	83.7	79.1	69.4	73.2	72.4	53.2	51.6	53.5
NAD1	84.4	84.4	84.8	74.4	69.3	69.6	69.5	48.4	52.3	50.1
NAD2	72.5	71.1	69.7	50.1	53.5	48.1	52.2	35.4	30.8	35.2
NAD3	81.9	81.9	75.6	67.5	64.8	67.5	63.9	38.3	37.5	39.2
NAD4	79.2	79.4	80.6	69.6	67.4	65.0	65.0	45.2	44.4	44.1
NAD4L	93.5	93.5	92.2	65.8	71.4	68.8	64.9	30.3	37.5	33.7
NAD5	78.5	77.9	79.1	67.3	63.2	64.7	64.5	42.1	39.1	39.9
NAD6	67.3	70.1	70.8	48.6	58.3	57.6	52.7	27.8	28.4	29.8
*rrn*L	83.4	82.6	80.2	73.9	69.2	70.1	67.5	63.9	62.8	61.6
*rrn*S	86.8	85.6	86.0	75.6	74.5	71.2	70.6	61.1	61.3	60.5

With those published for species of Strongylida [*Ancylostoma duodenale *(*Ad*), *Necator americanus *(*Na*) and *Haemonchus contortus *(*Hc*)], species of Ascaridida [*Anisakis simplex *(*Ans*), *Ascaris suum *(*Ass*) and *Toxocara canis *(*Tc*)] and species of Spirurida [*Brugia malayi *(*Bm*), *Dirofilaria immitis *(*Di*) and *Onchocerca volvulus *(*Ov*)].

The AT-rich regions for *Ancylostoma caninum *and *Bunostomum phlebotomum *were 272 bp and 234 bp, respectively, and both exhibited complex secondary structures (not shown), as predicted previously for the AT-rich regions of nematodes [41,42,45,47,49]. Four AT-repeat regions of variable length were identified in the AT-rich region of the mt genome of *Ancylostoma caninum*: two were 6 nucleotides (nt) (3 AT-repeats), one was 14 nt (7 AT-repeats) and the longest was 16 nt (8 AT-repeats). Similar dinucleotide repeats have been described in the AT-rich region of the mt genomes of other nematode species (e.g., [41,42,44]). Other repetitive elements have been identified within this region in the free-living nematode *Caenorhabditis elegans*, the largest and most conspicuous of which are the repetitive sequence motifs CR1-CR6 [45]. However, no such elements were identified in the AT-rich region of the mt genome of either *Ancylostoma caninum*, *Bunostomum phlebotomum *or any other species of animal-parasitic nematode sequenced to date [41,44,47,49].

### Comparative analyses with other nematodes

The identities (%) in inferred amino acid sequences of each protein-coding mt gene were calculated based upon pairwise comparisons between *Ancylostoma caninum *and *Bunostomum phlebotomum *(Tables 2 and 3). Based on these comparisons, the sequence identities (in decreasing order) were COX1 (97.3%), COX3 (96.0%), NAD4L (93.5%), COX2 (89.6%), CYTB (87.0%), ATP6 (85.9%), NAD1 (84.4%), NAD3 (81.9%), NAD4 (79.2%), NAD5 (78.5%), NAD2 (72.5%) and NAD6 (67.3%). In addition, the amino acid sequences inferred from each coding mt gene of *Ancylostoma caninum *and *Bunostomum phlebotomum *were compared, again in a pairwise manner, with those inferred from published mt genomes of *Anisakis simplex *[43], *Ascaris suum *[45] and *Toxocara canis *[44] (Ascaridida), *Ancylostoma duodenale*, *Necator americanus *[42] and *Haemonchus contortus *[41] (Strongylida), and *Brugia malayi *[46], *Dirofilaria immitis *[47] and *Onchocerca volvulus *[48] (Spirurida). The most conserved protein sequences among all species, assessed relative to *Ancylostoma caninum *and *Bunostomum phlebotomum*, were inferred to be COX1, COX3 and NAD4L, and the least conserved were NAD2 and NAD6 (see Tables 2 and 3).

### Phylogenetic analyses of selected species of Ascaridida, Spirurida and Strongylida using concatenated amino acid sequence data inferred from mt genes

**Figure 2:**
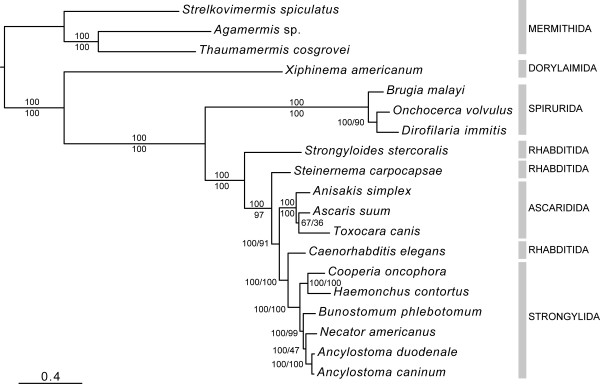
**Phylogenetic analysis (using Bayesian inference) of concatenated mt amino acid sequence data inferred from all protein coding mitochondrial genes (n = 12) for 16 secernentean nematodes, including *Ancylostoma caninum *and *Bunostomum phlebotomum *(GenBank accession numbers **FJ483518**and **FJ483517, **respectively)**. The concatenated mitochondrial amino acid sequence of three mermithids were employed as outgroups. Bayesian posterior probability values (as a percentage) and maximum likelihood bootstrap support (n = 100) are indicated above and below the lines, respectively. The scale indicates an estimate of substitutions per site, using the optimized model setting.

Because of the high degree of intraspecific variation in nucleotide sequence in the mt genes of nematodes [37,38,52] and the limited availability or lack of multiple mt genome sequences for each species, previous work has suggested that phylogenetic analyses for nematodes be conducted using concatenated amino acid sequence datasets, utilizing sequences inferred from individual mt protein coding genes [47]. In order to further assess systematic relationships within and among members of the Ascaridida, Spirurida and Strongylida, a phylogenetic analysis was carried out using Bayesian inference (BI) and maximum likelihood (ML) (Figure 2). Almost all clades in the consensus tree were supported by maximum BI posterior probability (pp) values (pp = 1.00; expressed as a percentage in Figure 2) and/or ML bootstrap support (100). The phylogenetic analysis conducted herein clearly supports the distinct classification of the orders Ascaridida, Spirurida and Strongylida, each as monophyletic clades with maximum statistical support. The order Rhabditida appears to be paraphyletic, with *Caenorhabditis elegans *grouping closely with the Strongylida, and *Steinernema carpocapsae *and *Strongyloides stercoralis *placed externally to a clade comprising the Ascaridida, Strongylida and *C. elegans*. This relationship is consistent with the proposed molecular phylogeny for the Nematoda based on small subunit (18S) nuclear ribosomal DNA data [53]. In addition, the hookworms were represented as a monophyletic clade within the Strongylida.

For hookworms, the phylogenetic analysis using BI indicated a closer relationship between *Ancylostoma *spp. and *Necator americanus *than between either of them and *Bunostomum phlebotomum*. This finding conflicts with the current classification of the Strongylida [16], wherein both *Necator *and *Bunostomum *are placed within the subfamily Bunostominae, whereas *Ancylostoma *is placed within the subfamily Ancylostominae (poorly supported by the ML analysis; bootstrap support = 47). A larger analysis, including mt data for more hookworm species, is needed to test further this hypothesis.

The present phylogenetic analysis did not support the grouping of the Ascaridida and Spirurida to the exclusion of the Strongylida, which contrasts markedly the results of a previous study based on nuclear ribosomal gene data (e.g., clade III *versus *clade V; ref. [53]). The "common heritage" hypothesized herein for the Ascaridida and Strongylida to the exclusion of the Spirurida has been supported by previous studies using mt gene order data [49] and using concatenated amino acid sequence data inferred from protein-coding mt genes [38]. These findings stimulate further study of the evolutionary relationships among taxa within this phylum using mt datasets. The high-throughput sequencing potential of 454 technology [54] and the recent validation of this technique for the sequencing of mt genomes [41] should provide a platform for an in-depth analysis of the phylogeny of the Nematoda.

## Conclusion

### Utility of mt gene markers for population genetic, ecological and epidemiological studies of hookworms

Although some nuclear genetic regions (e.g., ITS-1 and ITS-2 of nuclear rDNA [22-25] or the cAMP-dependent protein kinase gene [26]) have been shown to be suitable for the specific identification and differentiation of hookworms, the nuclear loci examined to date do not usually display sufficient levels of intraspecific sequence variability for the investigation of the genetic structures of hookworm populations (or the identification of population variants or "strains"). The ability to estimate genetic variability within and among hookworm populations is central to studying their epidemiology and population genetics, and can have important practical implications in relation to control.

Sequence-based analyses (including mutation scanning) of protein-coding mt genes, such as *cox1 *and *nad1*, have been particularly useful or population genetic studies [21,27,29-31,55-59]. For example, Hu *et al*. [21] employed a single-strand conformation polymorphism (SSCP)-coupled sequencing approach to explore haplotypic variability within a limited number of *Ancylostoma caninum *specimens from Australia and each of the human hookworms (*Ancylostoma duodenale *and *Necator americanus*). Significant population sub-structuring was recorded within each of these three species, and two genetically distinct subpopulations were detected within *Ancylostoma caninum *from dogs from Townsville, Australia. Previous morphological and clinical studies had shown that *Ancylostoma caninum *in Townsville (Australia) is not specific to dogs and can also infect humans (but not complete its life-cycle), causing eosinophilic enteritis [19]. It has been speculated [21] that particular, genetically distinct subpopulations within *Ancylostoma caninum *can selectively infect the non-canine host. The pattern of haplotypic variability within *Ancylostoma caninum *might be due to secondary contact between populations or subpopulations, which could have arisen due to host movement from other geographical areas where this hookworm has been recorded and where ecological conditions are distinct; for example, *Ancylostoma caninum *is endemic in tropical north-east Queensland, Australia [60], but also occurs in the north-west area of Western Australia [61]. It is also possible that feral dogs or dingoes (in different geographical or climatic regions) might harbour one or more genetic variants which might "spill-over" into domestic dogs and/or humans [60]. Future study of the genetic variation among *Ancylostoma caninum *specimens from domestic and feral dogs, cats and humans as well as between populations from other geographical and climatic regions in Australia and South-East Asia would allow such questions to be addressed. A comparison of the genetic make-up of *Ancylostoma caninum *from humans affected by eosinophilic enteritis with those from domestic dogs in the Townsville area would be particularly interesting.

In contrast to *Ancylostoma caninum*, no studies have yet explored the genetics or molecular epidemiology of *Bunostomum phlebotomum*. Mitochondrial markers might be used to examine sub-structuring in *Bunostomum phlebotomum *populations in endemic regions of South Africa. In addition, although there has been anecdotal evidence suggesting that *Bunostomum phlebotomum *may cause cutaneous larval migrans in humans ([20] and unpublished observations [JVW]), the zoonotic potential of this species of hookworm has not yet been tested molecularly. In view of the lack of distinguishing morphological characters allowing the identification of individual larvae, the provision of molecular markers for *Bunostomum phlebotomum *might allow the extent of the zoonotic potential of this species to be assessed for the first time.

The two mt genomes characterized herein provide a solid foundation for studies of the epidemiology, ecology and population genetics of both *Ancylostoma caninum *and *Bunostomum phlebotomum*, which could have important implications for the control of infections by these parasites. Given the lack of morphological characters for specific identification and differentiation of hookworm larvae, there is a clear need for species and population genetic markers for in-depth exploration of the epidemiology of hookworms [59]. Combined with the use of specific markers in the internal transcribed spacers (ITS-1 and ITS-2) of nuclear rDNA [23-25], investigating the mt haplotypic variability in populations of *Ancylostoma caninum *and *Bunostomum phlebotomum *(irrespective of developmental stage) could provide important insights into host affiliations, gene flow and transmission patterns (cf. [62,63]) and thus assist in the control of these hookworms. Furthermore, the direct sequencing of the mt genome of *Ancylostoma caninum *by 454 technology is the second example of the use of this approach for the sequencing of mt genomes of nematodes [41] and re-enforces the exciting potential of emerging technologies for the high-throughput sequencing of relatively small organellar genomes.

## Methods

### Parasites and DNA extraction

An adult male of *Ancylostoma caninum *(designated Ac1) was collected (by IB) at necropsy from the duodenum of a dog from Townsville, Australia [23]. An adult male of *Bunostomum phlebotomum *(Bp1) was collected at autopsy from the same site from a calf monospecifically infected with an isolate of *Bunostomum phlebotomum*, originally derived from a Jersey cow in Pretoria North suburb, South Africa (by JvW). Nematodes were washed in physiological saline, identified morphologically to species [16], fixed in 50% (v/v) ethanol and stored at -20°C until use. Total genomic DNA was isolated from individual worms using sodium dodecyl-sulphate/proteinase K treatment [64], followed by spin-column purification (Wizard Clean-Up, Promega). The specific identity of each nematode was verified using the sequence of the second internal transcribed spacer (ITS-2) of nuclear ribosomal DNA, which provides species-specific genetic markers for hookworms [25]. The ITS-2 sequence derived from sample Ac1 was identical to that reported previously for *Ancylostoma caninum *(accession number AJ001591) [25] and that obtained from Bp1 (accession number FJ616999) was 82.3% identical to the closely related species *Bunostomum trigonocephalum *(accession number AJ001595) [25].

### Long PCR-coupled mt genome sequencing

The complete mt genome of each *Ancylostoma caninum *and *Bunostomum phlebotomum *was amplified as two overlapping amplicons (~10 kb and ~5 kb, respectively) from ~20–40 ng of the genomic DNA from each specimen by long-PCR (BD Advantage 2, BD Biosciences) using each of the primer pairs 39F-42R and 5F-40R [39,40,42], as described by Hu *et al*. [39], with minor modifications. The cycling conditions (2720 thermal cycler, Applied Biosystems) were: 92°C, 2 min (initial denaturation); then 92°C, 10 s (denaturation); 50°C, 30 s (annealing); 68°C (for the ~10 kb region) or 60°C (for the ~5 kb region), 10 min (extension) for 10 cycles, followed by 92°C, 10 s; 50°C, 30 s; 68°C or 60°C, 10 min for 20 cycles, with an elongation period of 10 s for each cycle, and a final extension at 68°C or 60°C for 7 min. Following the PCR, individual amplicons were resolved in ethidium bromide-stained agarose (1%) gels and shown to represent single bands. Amplicon size was estimated based on comparison with a 1 kb DNA size ladder (Promega). Amplicons of ~10 kb or ~5 kb were purified over a mini-column (Wizard PCR Preps, Promega). Subsequently, the amount of DNA in each purified amplicon was estimated spectrophotometrically (ND-1000 UV-VIS spectrophotometer, v.3.2.1, NanoDrop Technologies). The purified amplicons were then subjected to sequencing.

The mt genome of *Ancylostoma caninum *(designated AcMG-454; GenBank accession no. FJ483518) was sequenced (454 technology by AJ/RBG) using a Genome Sequencer 20 (Roche), according to an established protocol [54]. The complete mt genome of *Bunostomum phlebotomum *(designated BpMG-PW; GenBank accession no. FJ483517) was sequenced (AW/DTL) by primer walking, as described previously [44]. The AcMG-454 sequence was assembled automatically, whereas that of BpMG-PW was assembled manually using Sequencher v.4.8 (Gene Codes Corporation). Both mt genome sequences were annotated and subjected to analysis using standard approaches [41,44], and their structures were compared with each other and with those of the two human hookworms, *Ancylostoma duodenale *(GenBank accession number AJ417718; ref. [42] and *Necator americanus *(AJ417719; ref. [42], *Haemonchus contortus *(EU346694; ref. [41]) (Strongylida); *Anisakis simplex *(AY994157; ref. [43], *Ascaris suum *(X53453; ref. [45]), and *Toxocara canis *(EU730761; ref. [44]) (Ascaridida); *Brugia malayi *(AF538716; ref. [46]), *Dirofilaria immitis *(AJ537512; ref. [47]) and *Onchocerca volvulus *(AF015193; ref. [48]) (Spirurida).

### Phylogenetic analysis

The analysis of amino acid sequence data was conducted *via *Bayesian inference (BI) using the software package MrBayes v.3.1.2  and maximum likelihood (ML) using GARLI ([65]; ), each running on a four dual-core Opteron-based Unix cluster. For individual species, the amino acid sequences inferred from all protein coding mt genes were concatenated. A selection of published mermithid mt genomes were used as outgroups (*Strelkovimermis spiculatus*, accession NC_008047; *Agamermis *sp., NC_008231; *Thaumermis cosgrovei*, NC_008046) and included a range of ingroup taxa (*Xiphinema americanum*, NC_005928; *Brugia malayi*, NC004298; *Onchocerca volvulus*, NC_001861; *Dirofilaria immitis*, NC_005305; *Strongyloides stercoralis*, NC_005143; *Caenorhabditis elegans*, NC_001328; *Steinernema carpocapsae*, NC_005941; *Necator americanus*, NC_003416; *Ancylostoma duodenale*, NC_003415; *Cooperia oncophora*, NC_004806; *Haemonchus contortus*, NC_010383; *Anisakis simplex*, NC_007934; *Ascaris suum*, NC_003127; *Toxocara canis*, NC_010690). Amino acid sequences were aligned using MUSCLE [66]. Ambiguous sites were excluded using G-Blocks ([67,68]; see Additional File 1 for alignment).

For BI of amino acid data, tree construction and posterior probabilities (pp) were calculated *via *2000000 generations (ngen = 2000000) using the Metropolis-coupled Monte Carlo Markov Chain (MCMCMC) method and four simultaneous tree-building chains (nchains = 4), with every 10^th ^tree being saved (samplefreq = 10). A suitable burnin (burnin = 1000) was chosen using 'Trace' in the program Tracer v1.4 . Evolutionary distance was estimated using the most appropriate amino acid model and calculated employing the MrBayes program (aamodelpr = mixed), allowing for a gamma-shaped variation in mutation rates with a proportion of invariable sites (rates = invgamma). Upon completion of the analysis, a 50% majority rule = consensus tree was constructed in TreeviewX v.0.5.0 . For the ML analysis using GARLI, tree construction was estimated with the model GTR+I+g using the mtRev amino acid substitution matrix, for two replicate runs, and termination criteria with setting genthresholdfortopoterm = 20000 (no new significantly better scoring topology found in > 20000 generations). Nodal support in the ML analysis was estimated by bootstrap re-sampling (n = 100) using GARLI and the same model settings.

## Authors' contributions

ARJ and RBG conceived the study. ARJ and AW contributed equally to molecular work, assisted by MH. DTJL provided technical advice and conducted phylogenetic analyses. IB and JvW provided morphologically-identified parasite material. ARJ and RBG drafted the manuscript, with active inputs from all other authors. All authors approved the final manuscript.

## Supplementary Material

Additional file 1 **Amino acid alignment of all nematode taxa used for phylogenetic analysis.** Alignment output from Gblocks. The Gblocks server  was used to align the concatenated amino acids using the most conserved settings. Blocks of "XXXX" represent markers between genes and were not included in the analyses; parameters are listed at the end of the alignment. Gblocks selected 2837 positions (marked *) to be included. Gene partitions are in the following order: *atp6*, *cox1*, *cox2*, *cox3*, *cytb*, *nad1*, *nad2*, *nad3*, *nad4*, *nad4L*, *nad5*, *nad6*. The concatenated mt genomic sequences used in the alignment represent species of Mermithida (*Agamermis *sp., *Thaumamermis cosgrovei *and *Strelkovimermis spiculatus*), Ascardida (*Anisakis simplex*, *Ascaris suum *and *Toxocara canis*), Dorylaimida (*Xiphenema americanum*), Rhabditida (*Caenorhabditis elegans*, *Steinernema carpocapsae *and *Strongyloides stercoralis*), Spirurida (*Brugia malayi*, *D. immitis *and *O. volvulus*) and Strongylida (*Ancylostoma caninum*, *Ancylostoma duodenale*, *Bunostomum phlebotomum*, *Cooperia oncophora*, *Haemonchus contortus *and *Necator americanus*). The species used as outgroups are denoted in bold text. Click here for file
