# Supplementary material for: The mitochondrial genomes of Ancylostoma caninum and Bunostomum phlebotomum – two hookworms of animal health and zoonotic importance
Source: BMC Genomics. 2009 Feb 11;10:79. doi: 10.1186/1471-2164-10-79 (PMC2656527; doi:10.1186/1471-2164-10-79)
Supplement: Additional file 1 — Amino acid alignment of all nematode taxa used for phylogenetic analysis. Alignment output from Gblocks. The Gblocks server was used to align the concatenated amino acids using the most conserved settings. Blocks of "XXXX" represent markers between genes and were not included in the analyses; parameters are listed at the end of the alignment. Gblocks selected 2837 positions (marked *) to be included. Gene partitions are in the following order: atp6, cox1, cox2, cox3, cytb, nad1, nad2, nad3, nad4, nad4L, nad5, nad6. The concatenated mt genomic sequences used in the alignment represent species of Mermithida (Agamermis sp., Thaumamermis cosgrovei and Strelkovimermis spiculatus), Ascardida (Anisakis simplex, Ascaris suum and Toxocara canis), Dorylaimida (Xiphenema americanum), Rhabditida (Caenorhabditis elegans, Steinernema carpocapsae and Strongyloides stercoralis), Spirurida (Brugia malayi, D. immitis and O. volvulus) and Strongylida (Ancylostoma caninum, Ancylostoma duodenale, Bunostomum phlebotomum, Cooperia oncophora, Haemonchus contortus and Necator americanus). The species used as outgroups are denoted in bold text. [file 1471-2164-10-79-S1.doc]

Supplementary Table 1.

Alignment output from Gblocks. The Gblocks server (<http://molevol.cmima.csic.es/castresana/Gblocks_server.html>) was used to align the concatenated amino acids using the most conserved settings. Blocks of “XXXX” represent markers between genes and were not included in the analyses; parameters are listed at the end of the alignment. Gblocks selected 2837 positions (marked *) to be included. Gene partitions are in the following order: *atp6*, *cox1*, *cox2*, *cox3*, *cytb*, *nad1*, *nad2*, *nad3*, *nad4*, *nad4L*, *nad5*, *nad6*. The concatenated mt genomes used in the alignment represent species of Mermithida (*Agamermis* sp., *Thaumamermis cosgrovei* and *Strelkovimermis spiculatus*), Ascardida (*Anisakis simplex*, *Ascaris suum* and *Toxocara canis*), Dorylaimida (*Xiphenema americanum*), Rhabditida (*Caenorhabditis elegans*, *Steinernema carpocapsae* and *Strongyloides stercoralis*), Spirurida (*Brugia malayi*, *D. immiti* and *O. volvulus*) and Strongylida (*Ancylostoma caninum*, *Ancylostoma duodenale*, *Bunostomum phlebotomum*, *Cooperia oncophora*, *Haemonchus contortus* and *Necator americanus*). The species used as outgroups are denoted in bold text.

60

=========+=========+=========+=========+=========+=========+

***Agamermis sp.*** ----------------------MIL--MSPFHSLNLSFSMLPMILVFVALMFMSYKLKN-

***T. cosgrovei*** ----------------------MVYNFMSPFHFMNLSMSAGVILMFIMMFSYLNFTPRSE

***S. spiculatus*** -MVMIGYFSFSFKVNWFTNYYKLINKMIIPFHFFNDYMSNWLMLCLLVIPFHLYIKPDLN

*X. americanum* MTTLFNVFCPLMCSFYPVSSQSLFILFTSMFCLVFLFAFSMEAFIPLWELLFPLEFSSFV

*B. malayi* -----------ILLFIIWFLMLFYLFYMELLKFGYLGFLGYFFNFLVSGFSHQGVQSSFF

*O. volvulus* ----------ILLFFFIWFWLVFYLFYMELSKFSSLGILGVFVNVLVSSFSHQGFQSNVF

*D. immitis* -----------LLFVFFWFFLVFYLFYMELSKFVLVG--GFFFDVMIFSFLHQGLQSSFF

*N. americanus* ------INQVFFLDVFMFVFFLQYMLYFKESMLNVLLKNFFKSLINVFSYSSSLPMSSVI

*C. oncophora* ------IKQVFFLDVFMFVFFLQYLLYFKEGMLNIVVKKFFVSLVEVFSYSKSLPMSSVV

*A. duodenale* ------INQVFFLDIFMFVFFLQYLLYFKESMLNSLVKKFFKGLIEVFSYTSVLPMSSVI

*A. caninum* ------INQVFFLDIFMFVFFLQYLLYFKESMLNTLVKKFFKGLIEVFSYTSILPMSSVI

*B. phlebotomum* ------INQVFFLDIFMFVFFLQYILYFKEGMLNVLVKNFLGSLVNVFSYSKCLPLSFVV

*H. contortus* ----------------MFVFFLQYMLYFNNSMINVLVKKFFLGLKEVFSYSVVLPLSSLI

*C. elegans* ------INQVYFLDIFMFVFVLQFLFYFKESMLNTLVKKFLNSLVGVFSYTNTLPLSSVI

*S. carpocapsae* ------INNVYFLDIFMFFYLLQFIFYFKESMMNVLVKKFFFGLIGVFSFSSSLPLSSII

*A. suum* ------ITNVYFLDIFMFVYVLQFLFYFKESMLGVLVNKFLGLLVVVFSYTDSLPLSSVI

*A. simplex* ------LSNVYFLDIFMFIYVLQFLFYFKESMINVLVKKFLGVLVTVFSYTEELPLSSVI

*T. canis* ------IGNVYFLDIFMFVYVLQFLFYFKESMLGVLFKKFMGGLVMVFGYSSDLPMSSVV

*S. stercoralis* ------LDFFNFFDFFLFVFILQFIFCQKDGLINVFSNKFFSFLVGVFSYNSSCFISYIF

--------------------------------------------------------****

120

=========+=========+=========+=========+=========+=========+

***Agamermis sp.*** --KIIYTIVMLNMLGLIPEFWSPTSYLWGNMLMGFFTWMLYSMNKIFNNFKFWSSHMLPI

***T. cosgrovei*** LFMIFGLLLYMNMLGLIPYSWGLTSFMMSNFFLGLLMWSSFYFYKFVNLPSVSLAHFLPV

***S. spiculatus*** -MVSLTVMLLLNVLGLVPYSWSWASYYWSVLILSILLFLMILLFNFSKQTNKFLSHLLPN

*X. americanum* FMFMFGLLLMFNLISLTLGSYTITTTLSFNLFFSFMYWVASILLFIFLKP-YSLSSLLPL

*B. malayi* FKFMVFFLTMFWFCGLVFPLFSPWASVGFLFFITNFSWLGVRTFTLAIDS--FMVCFEDD

*O. volvulus* FKFVVFFLLVFWMSGLLFPLFSPWACVGFLFFITNFSWLGVRTFTLAVDS--FLIFFESD

*D. immitis* FKFACFFLVMFWMSGLFFPLFSPWACVGFLFFLTNFSWLGARVLTLSMDS--FLVFFEDD

*N. americanus* SFFTFIVLFICCFGGYFTYSFCPCGMIEFTFVYAMVAWLSTLLSFISSEK--FSVYMSKG

*C. oncophora* SFFTFIVLLICCFGGYFTYSFXPCGMIEFTFVYAVVAWMSTLMTFMSSEK--FSVYMSKN

*A. duodenale* SFFTFIVLLICCFGGYFTYSFCPCGMVEFTFIYAMVAWMSTLLTFISSEK--FSIYMSKS

*A. caninum* SFFTFIVLLICCFGGYFTYSFCPCGMVEFTFIYAMVAWMSTLLTFISSEK--FSIYMSKS

*B. phlebotomum* SFFTFIILLICCFGGYFTYSFCPCGMVEFTFVYAMIAWMSTLLSFISSEK--FSVYMSKG

*H. contortus* SVFTFVVLLICCFGGYFCYSFCPCGMVEFTFVYAMMAWMSTLLSFMSSEK--FSIYMSKS

*C. elegans* SIFTFIVLLTCCFGGYFTYSFCPCGMVEFTFVYAAVAWLSTLLTFISSEK--FSVYMSKP

*S. carpocapsae* SVFTFIVLLVCCFGGYFTYSFCPCGMIEFTFVYAVVAWMSTFLTFISSEK--FSVFMSKE

*A. suum* SVFTFLVLLTCCFGGYFMYSFCPCGMIEFTFVYAMVAWLSTLLTFITSEK--FSIYISKA

*A. simplex* SIFTFIVLLTCCFGGYFSYSFCPCGMIEFTFVYAIVAWMSTFFTFITSEK--FSIYMAKE

*T. canis* SVFTFVVLLVCCFGGYFTYSFCPCGMVEFTFVYAVVAWMSTFLSFISSEK--FSVYISKP

*S. stercoralis* SSFIFVFLLTCCFGGYFSYSFCPCGIIEFTFFFSFVSWFSTFLFLISSQK--FSVYMKKS

**************************************************--********

180

=========+=========+=========+=========+=========+=========+

***Agamermis sp.*** GS-PLFLWSFLVILELLSQMIRPLTLSLRLTCNLMTGHVMLSLIMS-----SKHMILFLF

***T. cosgrovei*** SS-PLFLWSFLICLEVISQIIRPVTLSLRITCNIMAGHVVLSLVSN-----LNLVTICLF

***S. spiculatus*** GS-PIFLWNILIILETISFLIRPITLSLRLTCNMMAGHIVLSLVSN-----NMLTAMLIF

*X. americanum* GS-PMLLSPFLCMIELVSISARPITLCFRLLANMCAGHVILGLVFK-----ANWGVWLLG

*B. malayi* HSWEWVSSLVMFFSHWLSFLMSGVALTLRISIIFLIGHFL-MFTILDLGFFFS---FFSL

*O. volvulus* HSWEWFSSLVMFFSHWLSFLMSGVALTLRISIIFLIGHFL-MFTVLDMSVFYS---LFFL

*D. immitis* HSWDWVSSLVMFFSHFLSFFMSGVALTLRISIIFLIGHFL-MFTFLDFGFFCS---FFSL

*N. americanus* GD-KFLKTFSMLLVELVSEFSRPLALTVRLTVNIMVGHLISMMLYMGIENFMGEKYLWVS

*C. oncophora* ND-KFLKTFSMLLVEIVSEFSRPLALTVRLTVNIMVGHLISMMLYMGVENSLGEKYVWIS

*A. duodenale* GD-TFFKTFSMLLVEIVSEFSRPLALTVRLTVNIMVGHLISMMLYMGIENFLGEKYLWIS

*A. caninum* GD-TFLKTFSMLLVEIVSEFSRPLALTVRLTVNIMVGHLISMMLYMGIENFLGEKYLWIS

*B. phlebotomum* GD-IFLKTFSMLLVEIVSEFSRPLALTVRLTVNIMVGHLISMMLYMGIENYIGESFFWFS

*H. contortus* GD-KFLKTTSMLMVELVSEFSRPLALTVRLTVNIAVGHLISMMLYMMMENVVGEKYVWLT

*C. elegans* GD-TYLKTLSMLLIEIVSEFSRPLALTVRLTVNITVGHLVSMMLYQGLELSMGDQYIWLS

*S. carpocapsae* GD-SFLKTLSMLLVELVSEFSRPLALTVRLTVNIMVGHLISMMIYQFIEISSGVGYIWIT

*A. suum* GD-SFLKTFSMLLVELVSEVSRPLALTVRLTVNVLVGHVISMMLYQLLELYLGIFYVWIV

*A. simplex* GD-SFLKTFSMLLVEIVSEISRPLALTVRLTVNVLVGHMISMMLYQLLELTLGLGYVWLT

*T. canis* GD-SFLKTFSMLLVEIVSEVSRPLALTVRLTVNVLVGHMISMMLFQLLELFLGFGYVWLT

*S. stercoralis* GD-GFFKTFFLLFVEIISEFSRPIALSVRLFANIMVGHVISACFYVFFECIFG--FSFLI

-**-********************************************************

240

=========+=========+=========+=========+=========+=========+

***Agamermis sp.*** V-IMLFEMCVAVIQSVVFNLLLDSYKFE----XXXXXXXX---------------LSSVN

***T. cosgrovei*** ---LMFELVVGVIQAVVFSLLLYSYKTE----XXXXXXXX--------------MATSVN

***S. spiculatus*** ----YFELLVSIIQSHIFFILVNVYFKEM---XXXXXXXX--------------MMTSLN

*X. americanum* MPLMLLEVLVAFIQAFVFSMLISVYFQEAVSHXXXXXXXX------------MLWLTSCN

*B. malayi* LFIMPVELFFAFLQSYIFLTLICMFLLNMI--XXXXXXXXIFCGMTFGNVLKQSIINTVN

*O. volvulus* LLVVPVELFFAFLQSYIFLTLVCMFLLNMI--XXXXXXXXIFCGMTFGNSMKQSIINTVN

*D. immitis* LLLVPVELFFAFLQSYIFLTLICMFLCNMI--XXXXXXXXIFCGMTFGNSMKQSIFSTVN

*N. americanus* IFAIMMECFVFFIQSYIFSRLIYLYLNE----XXXXXXXX-INLYKKYQGGLATWLESSN

*C. oncophora* IFAIMMECFVFFIQSYIFSRLIYLYLNE----XXXXXXXXILNMYKKYQGGLSVWLESSN

*A. duodenale* ILAIMMECFVFFIQSYIFSRLIYLYLNE----XXXXXXXX-INLYKKYQGGLATWLESSN

*A. caninum* ILAIMMECFVFFIQSYIFSRLIYLYLNE----XXXXXXXX-INLYKKYQGGLATWLESSN

*B. phlebotomum* ILAIMMECFVFFIQSYIFSRLIYLYLNE----XXXXXXXX-INLYKKYQGGLSTWLESSN

*H. contortus* IFAIMMECFVFFIQSYIFSRLIYLYLNE----XXXXXXXX--NMYMKYQGGLSVWLESSN

*C. elegans* ILAIMMECFVFFIQSYIFSRLIFLYLNE----XXXXXXXX-INLYKKYQGGLAVWLESSN

*S. carpocapsae* ILAIMMECFVFFIQSYIFSRLIYLYLNE----XXXXXXXX-LNFKHSESSYFSVWLESSN

*A. suum* VLAIVMECFVFFIQSYIFSRLIYLYLNE----XXXXXXXX-ISGFYKYQGGLSVWLESSN

*A. simplex* VFAIMMECFVFFIQSYIFSRLIYLYLNE----XXXXXXXX-LKSFTKYQGGLSVWLESSN

*T. canis* IFAIMMECFVFFIQSYIFSRLIFLYLNE----XXXXXXXX--KNFYKYQGGLSIWLESSN

*S. stercoralis* VFAIIFECFVFILQSYIFSRLVYLYINE----XXXXXXXX-----------LVFVFYSTN

****************************-----------------------------***

300

=========+=========+=========+=========+=========+=========+

***Agamermis sp.*** HKHIGSMYFILSMWSGVMGISMSILVRLSLSDTSTN-WTLSNMYFLFYNTMTTSHALLMI

***T. cosgrovei*** HKHIGSMYFILSLWSGILGSSLSLVIRLSLSAPSIS-WNLSNLYFLFYNSVVTNHALLMI

***S. spiculatus*** HKNIGSLYFIFSVWMGLLGTSLSMFIRLSLSSTFVN-WTLSSSYFSFYNSVVTMHAILMI

*X. americanum* HKEIGILYLIQALALGVLGASTSVLMRWTLLLPLSSWWSQTANFSNFYNSVVTLHAFLMI

*B. malayi* HKTIGTYYIVLGYWAGLGGSLLSMIIRFELSSPGGYLFFGSG---QVYNSVLTMHGVLMI

*O. volvulus* HKTIGTYYIVLGYWAGLGGSVLSMLIRFELSSPGGHLFFGSG---QVYNSVLTMHGVLMI

*D. immitis* HKTIGTFYIVLGYWAGLGGSVLSMLIRFELSSPGGYLFFGSG---QVYNSVLTMHGVLMI

*N. americanus* HKDIGTLYFLFGLWSGMVGTSLSLIIRLELAKPGLLLGNGQ-----LYNSAITAHAILMI

*C. oncophora* HKDIGTLYFLFGLWSGMIGTSLSLIIRLELAKPGLLLSNGQ-----LYNSIITAHAILMI

*A. duodenale* HKDIGTLYFLFGLWSGMVGTSLSLIIRLELAKPGLLLGNGQ-----LYNSIITAHAILMI

*A. caninum* HKDIGTLYFLFGLWSGMVGTSLSLIIRLELAKPGLLLGNGQ-----LYNSIITAHAILMI

*B. phlebotomum* HKDIGTLYFLFGLWSGMVGTSLSLIIRLELAKPGLLLGNGQ-----LYNSIITAHAILMI

*H. contortus* HKDIGTLYFLFGLWSGMVGTSLSLIIRLELAKPGILLNNGQ-----LYNSIITAHAILMI

*C. elegans* HKDIGTLYFIFGLWSGMVGTSFSLLIRLELAKPGFFLSNGQ-----LYNSVITAHAILMI

*S. carpocapsae* HKDIGTLYFIFGLWSGMVGTSLSLIIRLELAQPGLLLGNGQ-----LYNSIITAHAILMI

*A. suum* HKDIGTLYFLFGLWSGMVGTSLSLVIRLELAKPGLLLGSGQ-----LYNSVITAHAILMI

*A. simplex* HKDIGTLYFLFGLWSGMVGTALSLIIRLELAKPGLFLGNGQ-----LYNSVITAHAILMI

*T. canis* HKDIGTLYFLFGLWSGMVGTGLSLIVRLELAKPGLFLGNGQ-----LYNSVITAHAILMI

*S. stercoralis* HKDIGSIYLIFGLWAGMIGSALSMIIRIELSKPGMFIGDGQ-----LYNSILTAHAILMI

*****************************************-----**************

360

=========+=========+=========+=========+=========+=========+

***Agamermis sp.*** FFMVMPAFIGGFGNWMLPIMMLCVDLIFPRLNSMSFWILPFSLWFLLMSITINSGPGSGW

***T. cosgrovei*** FFMVMPSLIGGFGNWMLPMFIGATDLAYPRLNSMSFWIMPFSLWFLLMSMLVESGAGTGW

***S. spiculatus*** FFMVMPSLIGGFGNWMVPLFLYSSDLIFPRLNSFSFWLMPFSFWFLMISMMVNMGPGVGW

*X. americanum* FFMVMPILIGGFGNILLPSMLGAQDMCFPRLNNFSFWLLPVSGLLMMSSMLLGCGAGTGW

*B. malayi* FFMVMPILIGGFGNWMLPVMLGAPEMAFPRVNALSFWFTFVALLMVYQSFFIGGGPGSSW

*O. volvulus* FFLVMPILIGGFGNWMLPLMLGAPEMAFPRVNALSFWFTFVALLMVYQSFFIGGGPGSSW

*D. immitis* FFMVMPILIGGFGNWMLPLMLGAPEMAFPRVNALSFWITFVALLMVYQSFFIGGGPGSSW

*N. americanus* FFMVMPSMIGGFGNWMLPLMLGAPDMSFPRLNNLSFWLLPTAMFLILDSCFVDMGCGTSW

*C. oncophora* FFMVMPSMIGGFGNWMLPLMLGAPDMSFPRLNNLSFWLLPTAMFLILDSCFVDMGCGTSW

*A. duodenale* FFMVMPTMIGGFGNWMVPLMLGAPDMSFPRLNNLSFWLLGTDMFLILDSCFVDMGCGTSW

*A. caninum* FFMVMPTMIGGFGNWMVPLMLGAPDMSFPRLNNLSFWLLPTAMFLILDSCFVDMGCGTSW

*B. phlebotomum* FFMVMPSMIGGFGNWMLPLMLGAPDMSFPRLNNLSFWLLPTAMFLILDSCFVDMGAGTSW

*H. contortus* FFMVMPTMIGGFGNWMLPLMLGAPDMSFPRLNNLSFWLLPTAMFLILDSCFVDMGAGTSW

*C. elegans* FFMVMPTMIGGFGNWLLPLMLGAPDMSFPRLNNLSFWLLPTSMLLILDACFVDMGCGTSW

*S. carpocapsae* FFMVMPSMIGGFGNWMLPLMLGAPDMSFPRLNNLSFWLLPTAMFLILDSCFVDTGCGTSW

*A. suum* FFMVMPTMIGGFGNWMLPLMLGAPDMSFPRLNNLSFWLLPTAMFLILDACFVDMGCGTSW

*A. simplex* FFMVMPTMIGGFGNWMLPLMLGAPDMSFPRLNNLSFWLLPTAMFLILDSCFVDMGSGTSW

*T. canis* FFMVMPTMIGGFGNWMLPLMLGAPDMSFPRLNNLSFWLLPTAMFLILDACFVDMGCGTSW

*S. stercoralis* FFMVMPSMIGGFGNWMVPLMLGAPDMSFPRLNNISFWLLPASIFLVFLACFVDNGLGTSW

************************************************************

420

=========+=========+=========+=========+=========+=========+

***Agamermis sp.*** TIYPPLSNE-GHLNSTIDYLIFSLHLAGISSILGSINFVVTSMIMRSKGMDWDRIPLFVW

***T. cosgrovei*** TIYPPLSSLSGHSNFSVDMLIFSLHLAGISSILGSINFMVSSLSYRMLMMSWDRIPLFVW

***S. spiculatus*** TMYPPLSNL--VDNMSMDFMILSLHIAGISSILSSINFLVTCIVCRPSVLTWDRISLFVW

*X. americanum* TIYPPLSSIAGHSNWSVDLVIFSLHLAGVSSIAGSINFLCTINNLKSSSISWMCMPLFLI

*B. malayi* TFYPPLSVE-GQPEVSLDVMILGLHTVGIGSLFGAINFMVTTQNMRSVAVTLDQASMFVW

*O. volvulus* TFYPPLSVE-GQPELSLDTMILGLHTVGIGSLLGAINFMVTTQNMRSTAVTLDQISMFVW

*D. immitis* TFYPPLSVE-GQPELSLDSMILGLHTVGIGSLLGAINFMVTVQNMRSTAVTLDQISMFVW

*N. americanus* TVYPPLSTL-GHPGSSVDLAIFSLHCAGLSSILGGINFMCTTKNLRSSSISLEHMSLFVW

*C. oncophora* TVYPPLSTL-GHPGSSVDLAIFSLHCAGLSSILGGINFMCTTKNLRSSSISLEHMSLFVW

*A. duodenale* TVYPPLSTL-GHPGSSVDLAIFSLHCAGLSSILGGINFMCTTKNLRSSSISLEHMSLFVW

*A. caninum* TVYPPLSTL-GHPGSSVDLAIFSLHCAGLSSILGGINFMCTTKNLRSSSISLEHMSLFVW

*B. phlebotomum* TVYPPLSTL-GHPGSSVDLAIFSLHCAGLSSILGGINFMCTTKNLRSSSISLEHMSLFVW

*H. contortus* TVYPPLSTL-GHPGSSVDLAIFSLHCAGLSSILGGINFMCTTKNLRSSSISLEHMSLFVW

*C. elegans* TVYPPLSTM-GHPGSSVDLAIFSLHAAGLSSILGGINFMCTTKNLRSSSISLEHMTLFVW

*S. carpocapsae* TVYPPLSTL-GHPGSSVDLAIFSLHCAGISSILGAINFMCTTKNLRSSSISLEHMSLFVW

*A. suum* TVYPPLSTM-GHPGGSVDLAIFSLHCAGVSSILGAINFMTTTKNLRSSSISLEHMSLFVW

*A. simplex* TIYPPLSTM-GHPGSSVDLAIFSLHCAGVSSILGAINFMTTTKNLRSSSISLEHMSLFVW

*T. canis* TVYPPLSTM-GHPGSSVDLAIFSLHCAGVSSILGAINFMTTTKNLRSSSISLEHMSLFVW

*S. stercoralis* TIYPPLSTS-GHPGSSVDLAIFSLHLSGISSILGGINFMCTIKNLRSSSVSLNNMSLFIW

******** **************************************************

480

=========+=========+=========+=========+=========+=========+

***Agamermis sp.*** SIIVTILLLIISLPVLAGAITMLLTDRNFNTSFYVSSGGGDPILFQHLFWFFGHPEVYVL

***T. cosgrovei*** SVLVTIFLLIISLPVLAGAITMLLTDRNFNTSFYYSSGGGDPILFEHLFWFFGHPEVYIL

***S. spiculatus*** SVMITVFLLLLSLPVLAGAITMLLTDRNFNTSFFISSGGGDPVLYQHLFWFFGHPEVYIL

*X. americanum* SVWVTAFLLVLSLPVLAGGITMLLFDRNLNTSFFDPLGGGDPILFQHLFWFFGHPEVYIL

*B. malayi* TSYLTSFLLVLSVPVLAGSLLFLLFDRNFNTSFYDTKNGGNPLLYQHLFWFFGHPEVYVI

*O. volvulus* TSYLTSFLLVLSVPVLAGSLLFLLLDRNFNTSFYDTKKGGNPLLYQHLFWFFGHPEVYVI

*D. immitis* TSYLTSFLLVLSVPVLAGSLLFLLLDRNFNTSFYDANKGGNPLLYQHLFWFFGHPEVYVI

*N. americanus* TVFVTVFLLVLSLPVLAGAITMLLTDRNLNTSFFDPSTGGNPLIYQHLFWFFGHPEVYIL

*C. oncophora* TVFVTVFLLVLSLPVLAGAITMLLTDRNLNTSFFDPSTGGNPLIYQHLFWFFGHPEVYIL

*A. duodenale* TVFVTVFLLVLSLPVLAGAITMLLTDRNLNTSFFDPSTGGNPLIYQHLFWFFGHPEVYIL

*A. caninum* TVFVTVFLLVLSLPVLAGAITMLLTDRNLNTSFFDPSTGGNPLIYQHLFWFFGHPEVYIL

*B. phlebotomum* TVFVTVFLLVLSLPVLAGAITMLLTDRNLNTSFFDPSTGGNPLIYQHLFWFFGHPEVYIL

*H. contortus* TVFVTVFLLILSLPVLAGAITMLLTDRNLNTSFFDPSTGGNPLIYQHLFWFFGHPEVYIL

*C. elegans* TVFVTVFLLVLSLPVLAGAITMLLTDRNLNTSFFDPSTGGNPLIYQHLFWFFGHPEVYIL

*S. carpocapsae* AVFVTVFLLVLSLPVLAGAITMLLTDRNLNTSFFDPSSGGNPLIYQHLFWFFGHPEVYIL

*A. suum* TVFVTVFLLVLSLPVLAGAITMLLTDRNLNTSFFDPSTGGNPLIYQHLFWFFGHPEVYIL

*A. simplex* TVFVTVFLLILSLPVLAGAITMLLTDRNLNTSFFDPSTGGNPLIYQHLFWFFGHPEVYIL

*T. canis* TVFVTVFLLVLSLPVLAGAITMLLTDRNLNTSFFDPSTGGNPLIYQHLFWFFGHPEVYIL

*S. stercoralis* TIFVTVFLLVLSLPVLAGAITMLLIDRNFNGSFFDPSFGGNPLIYQHLFWFFGHPEVYIL

************************************************************

540

=========+=========+=========+=========+=========+=========+

***Agamermis sp.*** ILPAFGIMSHLTMVEGNKKAVFGFLGMVYAMMSIALLGCVVWAHHMFSVGLDLDSRSFFT

***T. cosgrovei*** ILPAFGILSHLTMTLSGKKNVFGYLGMIYAMVSIGLLGCVVWAHHMFSVGLDIDSRSYFT

***S. spiculatus*** ILPAFGIISQATIMLSNKKNAFGYLGMIYAMLSIGLLGCVVWAHHMFTVGLDIDSRSYFT

*X. americanum* ILPGFGLVSHAVMISSGKPSPFGVPGMFLAITSIGVLGCVVWAHHMFSVGLDMDTRLYFT

*B. malayi* ILPVFGIISEAVLFLTDKDRLFGQTSMTFASIWIAVLGTSVWGHHMYTAGLDIDTRTYFS

*O. volvulus* ILPVFGIISEAVLFLTDKDRLFGQTSMTFASIWIAVLGTSVWGHHMYTAGLDIDTRTYFS

*D. immitis* ILPVFGIVSECVLFLTDKDRLFGQTSMTFASIWIAVLGTSVWGHHMYTAGLDIDTRTYFS

*N. americanus* ILPAFGIISQSTLYLTGKKEVFGSLGMVYAILSIGLIGCVVWAHHMYTVGMDLDSRAYFT

*C. oncophora* ILPAFGIISQSSLYLTGKKEVFGSLGMVYAILSIGLIGCVVWAHHMYTVGMDLDSRAYFT

*A. duodenale* ILPAFGIVSQSTLYLTGKKEVFGSLGMVYAILSIGLIGCVVWAHHMYTVGMDLDSRAYFT

*A. caninum* ILPAFGIVSQSTLYLTGKKEVFGSLGMVYAILSIGLIGCVVWAHHMYTVGMDLDSRAYFT

*B. phlebotomum* ILPAFGIISQSTLYLTGKKEVFGSLGMVYAILSIGLIGCVVWAHHMYTVGMDLDSRAYFT

*H. contortus* ILPAFGIVSQSSLYLTGKKEVFGSLGMVYAILSIGLIGCVVWAHHMYTVGMDLDSRAYFT

*C. elegans* ILPAFGIVSQSTLYLTGKKEVFGALGMVYAILSIGLIGCVVWAHHMYTVGMDLDSRAYFS

*S. carpocapsae* ILPAFGIVSQSTLYLTGKKEVFGSLGMVYAILSIGLIGCVVWAHHMYTVGMDLDSRAYFT

*A. suum* ILPAFGIISQSSLYLTGKKEVFGSLGMVYAILSIGLIGCVVWAHHMYTVGMDLDSRAYFT

*A. simplex* ILPAFGIISQSSLYLTGKKEVFGSLGMVYAILSIGLIGCVVWAHHMYTVGMDLDSRAYFT

*T. canis* ILPAFGIISQSSLYLTGKKEVFGSLGMVYAILSIGLIGCVVWAHHMYTVGMDLDSRAYFT

*S. stercoralis* ILPAFGIISQCTLYLTGKKEVFGYLGMVYAILSIGLIGCVVWAHHMYTVGMDFDSRAYFT

************************************************************

600

=========+=========+=========+=========+=========+=========+

***Agamermis sp.*** GASMVIAIPTGIKVFSWLSTLYGTMSNFDALYLWGVGFLFLFTLGGLTGITLSSSSLDLI

***T. cosgrovei*** AATMIIAIPTGIKIFSWIATMYGSSLKLHVLSLWGLGFLFLFTLGGLTGITLSSSSLDVL

***S. spiculatus*** SATMIIAVPTGIKIFSWVATMYSSSINMSPLVLWVSGFLFMFTLGGLTGITLSSSSLDLL

*X. americanum* AATMIIAVPTGIKVFSWLASFSGSKMLMMPLQLWILGFLFLFTVGGLTGIVLANGTLDLL

*B. malayi* AATMIIAIPSAVKIFNWLGTFFGSNQKMQPLWCWTYSFIFLFTVGGLSGIILSAASLDVV

*O. volvulus* AATMIIAIPSAVKIFNWLGTFFGSNQKMQPLWCWTYSFIFLFTVGGLSGIILSAASLDII

*D. immitis* AATMIIAIPSAVKIFNWLGTFFGSHQKIQPLWCWTYSFIFLFTIGGLSGIILSSASLDII

*N. americanus* AATMVIAVPTGVKVFSWLATMFGMKMVFQPVLLWVLGFIFLFTIGGLTGVILSNSSLDII

*C. oncophora* AATMVIAVPTGVKVFSWLATLFGMKMKFQPVLLWVLGFIFLFTIGGLTGVVLSNSSLDII

*A. duodenale* AATMVIAVPTGVKVFSWLATLFGMKMIFQPLLLWVLGFIFLFTIGGLTGVVLSNSSLDII

*A. caninum* AATMVIAVPTGVKVFSWLATLFGMKMNFQPLLLWVLGFIFLFTIGGLTGVVLSNSSLDII

*B. phlebotomum* AATMVIAVPTGVKVFSWLATLFGMKMIFQPVLLWVLGFIFLFTIGGLTGVILSNSSLDII

*H. contortus* AATMVIAVPTGVKVFSWLATLFGMKMNFQPVLLWVLGFIFLFTIGGLTGVILSNSSLDII

*C. elegans* AATMVIAVPTGVKVFSWLATLFGMKMVFNPLLLWVLGFIFLFTLGGLTGVVLSNSSLDII

*S. carpocapsae* AATMVIAVPTGVKVFSWLATLFGMKMLFQPVLLWVLGFIFLFTIGGLTGVILSNSSLDII

*A. suum* AATMVIAVPTGVKVFSWLATLFGMKMVFQPLLLWVMGFIFLFTIGGLTGVMLSNSSLDII

*A. simplex* AATMVIAVPTGVKVFSWLATLFGMKMVFQPLLLWVLGFIFLFTVGGLTGVVLSNSSLDVI

*T. canis* AATMVIAVPTGVKVFSWLATLFGMKMVFQPLLLWVLGFIFLFTIGGLTGVMLSNSSLDII

*S. stercoralis* AATMVIAVPTGVKVFSWLATFFGTVMVYQPLLFWVMGFIFLFTIGGLTGVMLSNSSIDII

************************************************************

660

=========+=========+=========+=========+=========+=========+

***Agamermis sp.*** LHDTYFVVGHFHFVLSMGAVFGIIMGMTLWFPMILGISFNHILAFSIFWVLFIGVNLTFI

***T. cosgrovei*** LHDTYFVVGHFHFVLSLGAVYGILSGVMISFPIILGIEMNYLLSTAMFLVLFVGVNLTFI

***S. spiculatus*** LHDTYFVVGHFHFVLSMGAVFGIMAGFTMWYPVVMSISFNYNLMSMSFWTLFIGVNLTFI

*X. americanum* YHDTYYVVAHFHYVLSMGAVFTIMIGLVNWWPVMTGLALNNTLSLTQFFMLFMGVNITFF

*B. malayi* LHDTYYVVAHFHYTLSLGAVYGIFCGFCLWLPYMYGVSFDGLLMIAIFFCFFIGTNMTFF

*O. volvulus* LHDTYYVVAHFHYTLSLGAIYGIFCGFCLWLPYMYGISFDSVMMMAVFVCFFVGTNMTFF

*D. immitis* LHDTYYVVAHFHYTLSLGAVYGIFCGFCLWLPYMYGISFDSLMMMAVFVCFFFGTNMTFF

*N. americanus* LHDTYYVVSHFHYVLSLGAVFGIFTGISLWWSFMTGYVYDKLMMSSVFFLMFFGVNLTFF

*C. oncophora* LHDTYYVVSHFHYVLSLGAVFGIFTGISLWWSFMTGYVYDKLMMSAVFFLMFFGVNLTFF

*A. duodenale* LHDTYYVVSHFHYVLSLGAVFGIFTGISLWWSFMTGYVYDKLMMSSVFFLMFVGVNLTFF

*A. caninum* LHDTYYVVSHFHYVLSLGAVFGIFTGISLWWSFMTGYVYDKLMMSSVFFLMFVGVNLTFF

*B. phlebotomum* LHDTYYVVSHFHYVLSLGAVFGIFTGISLWWSFMTGFVYDKLMMSSVFFLMFVGVNLTFF

*H. contortus* LHDTYYVVSHFHYVLSLGAVFGIFLGINLWWSFMTGYVYDKLMMSVVFFLMFFGVNLTFF

*C. elegans* LHDTYYVVSHFHYVLSLGAVFGIFTGVTLWWSFITGYVLDKLMMSAVFILLFIGVNLTFF

*S. carpocapsae* LHDTYYVVSHFHYVLSLGAVFGIFTGVSLWWSFITGFVYDKMVMCAVFFLMFVGVNMTFF

*A. suum* LHDTYYVVSHFHYVLSLGAVFGIFTGVTLWWSFITGFVYDKMMMSSVFVLMFVGVNLTFF

*A. simplex* LHDTYYVVSHFYYVLSLGAVFGIFTGISLWWGFMTGFVYDKMVMSVVFILMFIGVNTTFF

*T. canis* LHDTYYVVSHFHYVLSLGAVFGIFTGISLWWSFMTGFVYDKMMMSSVFVLMFVGVNLTFF

*S. stercoralis* LHDTYYVVSHFHYVLSLGAVFGLFAGVTLWWSFITGFVLNKIYMYSVFFLFFIGVNMTFF

************************************************************

720

=========+=========+=========+=========+=========+=========+

***Agamermis sp.*** PQHFMGLNGMPRRYGDYLDEYLFMHSISSMGSWISLTAMLISFFMIYESIMSNRKTLHLN

***T. cosgrovei*** PHHFMGLNGMPRRYGDYLDSYLIMHSLSSWGSWISMMGLLLFLFLTFESILAARKVLHCN

***S. spiculatus*** PHHFMGLNGMPRRYAEFMDSFLGMHVMSSWGSYISSASFIIIIIMMVESLVTCNKVLHLN

*X. americanum* PMHFLGIQGMPRRYSDYLTTFSFWHSFSSTGSLMSLIATLLLFFIWWESFVSMRVILSIL

*B. malayi* PMHFAGMQGMPRKVLDYPDCYSMFQVFSSLGSVITFIGFMLFNYLMIDSIFFSRFLGISF

*O. volvulus* PMHFAGLQGMPRKILDYPDCYSTFQIISSLGSVITFVGFVLFNYLLIDSIFFSRFLGVSF

*D. immitis* PMHFAGLQGMPRKILDYPDCFSTFQIISSLGSVITFVGFILFNYLMIDSVFLSCFLGVSF

*N. americanus* PLYFAGLHGFPRKYLDYPDVYSVWNVMSSYGSMISVFALFLFLYVLIESFFSYRLVLNDS

*C. oncophora* PLHFAGLHGFPRKYMDYPDVYSVWNVLSSYGSMVSVFAMFLFLYVLIESFFSYRLVLVDN

*A. duodenale* PLHFAGLHGFPRKYLDYPDVYSVWNVMSSYGSMISVFALFLFLYVLFESFFSYRLVLSDN

*A. caninum* PLHFAGLHGFPRKYLDYPDVYSVWNVMSSYGSMISVFALFLFLYVLFESFFSYRLVLSDN

*B. phlebotomum* PLHFAGLHGFPRKYLDYPDVYSVWNVMSSYGSMISVFALFLFLYVLLESFFSYRLVLSDN

*H. contortus* PLHFAGLHGFPRKYMDYPDVYSVWNVMSSYGSMVSVFALFMFLYVLIESFFSNRMVLIEK

*C. elegans* PLHFAGLHGFPRKYLDYPDVYSVWNIIASYGSIISTAGLFLFIYVLLESFFSYRLVISDY

*S. carpocapsae* PLHFAGLHGYPRKYLDYPDIYSVWNIVASYGSLISTFALFLFIYVLLESFWSCRLVMSDY

*A. suum* PLHFAGIHGYPRKYLDYPDVYSVWNIMASYGSMISVFALFLFIYVLLESFVGHRIFLFDY

*A. simplex* PLHFAGLHGFPRKYLDYPDVYSVWNIMASYGSMITVFALFLFIYALLESFMGHRILLCDY

*T. canis* PLHFAGLHGYPRKYLDYPDVYSVWNILASYGSMISVFALFLFXYVLLESFVGHRLLLLDY

*S. stercoralis* PLHFAGLHGFPRKYVDYPDVYSFWNVLSSFGSLISIFSLFFFVFVLLESFISYRVLVVDY

************************************************************

780

=========+=========+=========+=========+=========+=========+

***Agamermis sp.*** HINSGESMIMMSPPEHMNYQPHVFEQHPY-----------------------XXXX-MKN

***T. cosgrovei*** FLNT-EFLSNISPSEYLTSHYIIYS---------------------------XXXX---I

***S. spiculatus*** YINS---DMLMYSSEHLNNTLFSSKL--------------------------XXXXMTTT

*X. americanum* WGSTMIDSMNKGPTMLHSYSETPACFKTS-----------------------XXXX---M

*B. malayi* YNGHSPSYVSNVPPLPDSFLEDILGYGLCWKVFCKNTPSYSYRRVGYGYYSKXXXX----

*O. volvulus* YNYHSPAYALNVPPLPDSFTEEAFIMGLHWKIISKDTPSYSYRRVGYGYHSKXXXX----

*D. immitis* YNYHSPAYTINVPPLSDSFTEETFIMGLHWKVISSDTPSYSYRRVGYGYHSKXXXX----

*N. americanus* FINSSPEYSLSSYVFGHSYQSEIYFSCSVIK---------------------XXXX---M

*C. oncophora* FVNSSPEYSYSSYVFGHSYQSDIYFSSSVMKX--------------------XXXX---I

*A. duodenale* FINSSPEYSLSSYVFGHSYTSEIYFSCSVLKL--------------------XXXX---I

*A. caninum* FINSSPEYSLSSYVFGHSYQSEIYFSCSVLKL--------------------XXXX---M

*B. phlebotomum* FVNSSPEYSLSSYVFGHSYQSEVYFSCSVMK---------------------XXXX---I

*H. contortus* FINSSPEYSLSSYVFGHSYQSEIYFSSSVL----------------------XXXX---M

*C. elegans* YSNSSPEYCMSNYVFGHSYQSEIYFSTTSLKN--------------------XXXX---I

*S. carpocapsae* YVNSSPEYSMGGYVFGHSYQSEIFFNSSFKI---------------------XXXX---F

*A. suum* YVNSGPEYSLSGYVFGHSYQSEIFYSSIVFKF--------------------XXXX---L

*A. simplex* NVNSSPEYTSSGYVFGHSYQSEIFYSSTVLKF--------------------XXXX---L

*T. canis* YINSSPEYSMSGYVFGHSYQSEVFYSSTVIKF--------------------XXXX---V

*S. stercoralis* FNGVSPENTNLGYNFYHSFQDGVYLSVPH-----------------------XXXX----

******************------------------------------------------

840

=========+=========+=========+=========+=========+=========+

***Agamermis sp.*** LLLAQYLGLNFMN-QMS-TEHYLDLYNDLVIYLELIIACTVMIMILSIILNTWFQN-VDH

***T. cosgrovei*** LKFSQYLKLNLAN-QMNLVSWELDLYNDLVMLVELNTALSVFTYLSYVSSNKWYQK-LNH

***S. spiculatus*** FNFSEYNCLNIMDLVYSLFGLDLDNFNNLVLFLEFMICTNVLGYIFFCSTNKFYIKAFSH

*X. americanum* PFWGESAFQNFSSGMMSSLETLHDPCYGLAGCYFGSCFFGEIWSVFFKSN----AILSLD

*B. malayi* -IYLQNYVFPVPSNSYVFCCYFIHNYYSHVIFFGFFIMFLVSFGLYFYGNSFKFNLKRSD

*O. volvulus* -IYLQNYVFPIPGNSYVFCCYYIHNYYSHIIFFGFFVMFLVSGGIYFFGNSFKFNLKRSD

*D. immitis* -IYFQNYIFPNPGSSYVYCCYYIHNYYSHVIFFGFFLMLLVCIGVYFFGSSFKFNLKRSD

*N. americanus* GNYFQGYNLNFSSSLFSSYMDWFHGFNCSLLLG--VLIFVVLLFFYLMLNNYYFKSKKIE

*C. oncophora* GNYFQGYNLNFSNSLFASYMDWFHSFNCSLLLG--VLTFVVVLLLYLVNNIWYFKSKKIE

*A. duodenale* GNYFQGYNLNFSNSMFSSYMDWFHSFNCSLLLG--VLVFVVMLFMYLMMNNFYFKSKKIE

*A. caninum* GNYFQGYNLNFSNSMFSSYMDWFHSFNCSLLLG--VLVFVVMLFMYLMMNNYYFKSKKIE

*B. phlebotomum* GNYFQGYNLNFSSSIFSSYMDWFHGFNCSLLLG--VLVFVVLLFLYLIFNSYYFKSKKIE

*H. contortus* NNYFQGLNLNFSNSLFSSYMDWFHNFNCSLLLG--VLVFVVILLLYLMMNNYYFKSKKIE

*C. elegans* NNFFQGYNLLFQHSLFASYMDWFHSFNCSLLLG--VLVFVTLLFGYLIFGTFYFKSKKIE

*S. carpocapsae* YNFFQGYNLGFSNSLFASYMDWFHNFNCSLLFG--VLVFVTLMFVYLILNSFYFKSKKIE

*A. suum* NNFFQDFNLLFSSSLFSSYMDWFYNFNCSLLFG--VLSFVSTMFVYLLLSSFYFKSKKIE

*A. simplex* NNFFQDFGLMFSNSLFSGYMDWFHNFNCSLLFG--VLSFVSVMFGYLLFSNFYFKSKKIE

*T. canis* NNFFQDFGLMFSNSLFSSYMDWFHNFNCSLLFG--VLSFVSTSFVYLLLSKFYFKSKKIE

*S. stercoralis* FNYFQGYMMGFSNNYLCSYMDWFHNFNCSLLFG--VLFFVFMVIVNLFVNSYYFKSHNIE

----*****************************--*************************

900

=========+=========+=========+=========+=========+=========+

***Agamermis sp.*** WIVLESIWTMSPSIVLLILGIPSIKMIYSLEYTNFFSNLSIKIVGYQWYWNYSFPEF-EV

***T. cosgrovei*** WGALELLWTLFPVSVLVVLGCPSLKMLYMQELYQFSSNLSVKVLGYQWYWSYDFLEW-SV

***S. spiculatus*** SMSLEVTWTLIPVVVLIFLGMPSLKILFLSEILMTKINISLKVTGYQWYWSYSFPEF-NI

*X. americanum* SLSLEVIWSCLPMVILASVAFPSLLLLSKQETLG-KPLFTLKMISNQWSWASEYDHHNSY

*B. malayi* SRMIELILQVLVVNFLVIMAGPGFWLIQYQGRMFRQSELTLKVIGHQWYWSYEYGDDSSL

*O. volvulus* SRMIELILQVLIVNFLIMMAGPGFWLIQYQGRMFRQSELTLKVIGHQWYWSYEYGDSGKL

*D. immitis* SRMIELVLQVLIVNFLIMMAGPGFWLIQYQGRMFRQSELTLKVIGHQWYWSYEYGDSGKL

*N. americanus* YQFGELLCSVFPTLILLMQMVPSLSLLYYYGLMNMDSNLTVKVTGHQWYWSYEFSDIPGL

*C. oncophora* YQFGELLCSIFPTLILLMQMVPSLSLLYYYGLMNLDSNLTVKVVGHQWYWSYEFSDIPGL

*A. duodenale* YQFGELLCSVFPTLIFLMQMVPSLSLLYYYGLMNLDSNLTIKVTGHQWYWSYEFSDIPGL

*A. caninum* YQFGELLCSVFPTLILLMQMIPSLSLLYYYGLMNLDSNLTIKVTGHQWYWSYEFSDIPGL

*B. phlebotomum* YQFGELLCSVFPTLILLMQMVPSLSLLYYYGLMNLDSNLTVKVTGHQWYWSYEFSDVPGL

*H. contortus* YQFGELLCSIFPTLILLMQMVPSLSLLYYYGLMNLDSSLTVKVTGHQWYWSYEFSDIPGL

*C. elegans* YQFGELLCSIFPTIILLMQMVPSLSLLYYYGLMNLDSNLTVKVTGHQWYWSYEYSDIPGL

*S. carpocapsae* YQVGELLCSVFPTIILLFQMVPSLSLLYYYGLMSFDTGLTVKVTGHQWYWSYEFSDIPGL

*A. suum* YQFGELLCSVFPTLILVMQMVPSLSLLYYYGLMNLDSSLTVKVTGHQWYWSYEFSDIPGL

*A. simplex* YQFGELLCSIFPTLILVAQMVPSLSLLYYYGLMNLDSNLTVKVTGHQWYWSYEFSDIPGL

*T. canis* YQFGELLCSVFPTLILVMQMVPSLSLLYYYGLMNLDSNLTVKVTGHQWYWSYEFSDIPGL

*S. stercoralis* YQFGEFLCSLLPLFILVFQMFPSLSLLYFSGLSYLDSSLTVKVVGHQWYWSYDYSDI-GI

************************************************************

960

=========+=========+=========+=========+=========+=========+

***Agamermis sp.*** EIDSYPKLMSDLFR-----MGESNLLVLPIYTNLRLLMTSNDVIHSWALPSISLKMDAVP

***T. cosgrovei*** SLDSFPKMFSNLYR-----LGESELLVLPVSFNIRLLISSEDVIHSWTIPSLGVKMDATP

***S. spiculatus*** SLMSYPNYLALFYR-----LAESDLLILPMSSSIQTLITSQDVIHSWALPSLGFKMDACP

*X. americanum* DHLLDFDEIEVLSN-------LETPVFLPSGKVVRILLSSSDVLHSLGLPSLGVKLDSVP

*B. malayi* VFDSFMKPVDDLSLGDFRLFDVDNRCVLPIGVNIGLYCTSSDVIHSFAVPKCFVKMDALN

*O. volvulus* CFDSFMKSLDDLSLGDFRLFDVDNRCVLPVGVNVGVYCTSSDVIHSFAIPKCFIKMDALN

*D. immitis* CFDSFMKSLDDLSLGDYRLFEVDNRCVLPVGVNVGIYCTSSDVIHSFAVPKCFVKMDALN

*N. americanus* EFDSYMKSLDQLELGEPRLLEVDNRCVVPCDTNIRFCITSADVIHSWSLSSLSIKLDAMS

*C. oncophora* XXXSYMKSLDQLNVGEPRLLEVDNRCVVPCNTNIRFCITSADVIHSWALSSLSVKLDAMS

*A. duodenale* EFDSYMKYLDQLELGEPRLGEVDNRCVVPCETNIRFCITSADVIHSWALPSMSVKLDAMS

*A. caninum* EFDSYMKSLDQLELGEPRLLEVDNRCVVPCDTNIRFCITSADVIHSWALPSMSVKLDAMS

*B. phlebotomum* EFDSYMKSLDQLNLGEPRLLEVDNRCVVPCDTNIRFCITSADVIHSWSLLLLSVKLDAMS

*H. contortus* EFDSYMKSLDQLNLGEPRLLEVDNRCVIPCGTNIRFCITSADVIHSWALSSLSIKLDAMS

*C. elegans* EFDSYMKSLDQLSLGEPRLLEVDNRCVIPCDTNIRFCITSADVIHAWALNSLSVKLDAMS

*S. carpocapsae* EFDSYMKSLDQLDLGEPRLLEVDNRCIVPCDVNIRFCITSADVIHAWALSTLSIKLDAMS

*A. suum* EFDSYMKSLDQLELGEPRLLEVDNRCVVPCDVNIRFCITSGDVIHSWALPSMSIKLDAMS

*A. simplex* EFDSYMKSVDQLELGEPRLLEVDNRCVVPCDINVRFCITSGDVIHSWALPSMSIKLDAMS

*T. canis* EFDSYMKSLDQLELGEPRLLEVDNRCVVPCDTNIRFCITSGDVIHSWALPSMAIKLDAMS

*S. stercoralis* EFDSYMKSIDSLLIGEPRLLEVDNRCVVPCDTNIRFCITSADVIHSWSLPSFCIKLDAMS

************************************************************

1020

=========+=========+=========+=========+=========+=========+

***Agamermis sp.*** GRLNMLSTKILSIGNYMGQCSELCGNYHSWMPIYAESTNSIIFNQWIKLLFTFNYLTYTX

***T. cosgrovei*** GRLNFINILNNQPSKFIGQCSELCGNFHSWMPIYVEFSSTNIFIEWCKIYKI-------X

***S. spiculatus*** GRLNYYVLSSNLPSLHIGQCSELCGSYHSWMPIMVEFTSCSLFIEWLKNIL--------X

*X. americanum* GRINSTIID-GSSSVSIGSCYELCGTGHSAMPVSFILF---------------------X

*B. malayi* GLLTKITFNFSCCGLFYGQCSEICGANHSFMPIALELTSLECWKAWSLGYLLG------X

*O. volvulus* GLLTKVTCSFSCSGLFFGQCSEICGANHSFMPIVLELTSLECWKGWSVNYLLG------X

*D. immitis* GLLTKITCNFSCSGLFYGQCSEICGANHSFMPIVLELTSMECWKGWCVNFLLV------X

*N. americanus* GILSTLCYSFPVVGVFYGQCSEICGANHSFMPIAVEVTLLDNFKSWCYLNMN-------X

*C. oncophora* GILSTLCYSFPMIGVFYGQCSEICGANHSFMPIALEVTLMDNFKSWCYLNLEX------X

*A. duodenale* GILSTLCYSFPVVGVFYGQCSEICGANHSFMPIAIEVTLLDNFKSWCYLSMD-------X

*A. caninum* GILSTLCYSFPMVGVFYGQCSEICGANHSFMPIAIEVTLLDNFKSWCYLNMD-------X

*B. phlebotomum* GILSTLCYSFPVIGVYYGQCSEICGANHSFMPIAVEVTLLDNFKSWCFLNLG-------X

*H. contortus* GILSTLCYSFPLIGVFYGQCSEICGANHSFMPIALEVTLMENFKNWCLMNL--------X

*C. elegans* GILSTFSYSFPMVGVFYGQCSEICGANHSFMPIALEVTLLDNFKSWCFGTME-------X

*S. carpocapsae* GILSTISYSFPMIGVFYGQCSEICGANHSFMPIALEVTLLDNFKSWCLGMVE-------X

*A. suum* GILSTLSYSFPVVGVFYGQCSEICGANHSFMPVALEVTLLDNFKSWCVGLLSD------X

*A. simplex* GILSTVSYSFPTVGVFYGQCSEICGANHSFMPIALEVTLLDNFKSWCMGFMEN------X

*T. canis* GILTTLSYSFPVVGVFYGQCSEICGANHSFMPIVLEVTLLDNFKSWCLGLLDE------X

*S. stercoralis* GILSTVTCSFPMAGVFYGQSSEICGANHSFMPICLEVSLLENFKDWCYLMSS-------X

**********************************************--------------

1080

=========+=========+=========+=========+=========+=========+

***Agamermis sp.*** XXX------LQYNTSNSLWPLYTSFSMFLLLLTFYYYLKTTSALFDIS-YILLMINSKFW

***T. cosgrovei*** XXX----LTLQFNTGASWYPLFMSMSIFISILSFFMFLNFKLLTIWLS-LIFFGLMSFMW

***S. spiculatus*** XXX-ISLFILQSHLTKSWLPLYISFFLFLFLLSFLKFVTYKSMLFMLVVLMMLVLNMFIW

*X. americanum* XXX----------MSNSWWPLHMSLATFLIIMSLLSFLKGCFKLINCILLLPAMVIFFLW

*B. malayi* XXXIFLKFRKYHKMEFSYYPIMFGLGVLGIDFGLVLFMSMGIFCSFFICLLYLLYVSFLW

*O. volvulus* XXXILLKFRKYHKMEYSYYPLMVGVGILGFDVSLVLFMGMGMFYSIFICFLYLVYVFFLW

*D. immitis* XXXILLKFRKFHKMDYSYYPLMFSFGVFGVDVGLVLFMCLGMYYPFFFCFLYLIYVSFLW

*N. americanus* XXX---IFHNFHILSLSSYAYYMFFASLSLTSSFVIFFKYGLVVPFLFSLLVVLFISFAW

*C. oncophora* XXX---LLHNFHILSLSSYAYFMFFASLSLTSSFVVFFKYGLVLPFVFSLMVVLFIAFVW

*A. duodenale* XXX---IFHNFHILSLSSYAYYMFFASLGLTSSFVIFFKYGMVLPFLFSLFVVLFISFAW

*A. caninum* XXX---IFHNFHILSLSSYAYYMFFASLGLTSSFVIFFKYGMVLPFLFSLFVVLFISFAW

*B. phlebotomum* XXX---IFHNFHILSLSSYAYYMFFASLGLTSSFVIFFKYGLVFPFLFSLFVVLFISFAW

*H. contortus* XXX---MFHNFHILSLSSYAYYMFFSSLSLTSSFVIFFKYGLWLPFVFSLMVVLFISFAW

*C. elegans* XXX---MFHNFHILSLSSYAYNLFFASAGMLSSLVMFFKFGLYELFIFTLFSVLFISFAW

*S. carpocapsae* XXX---FFHNFHILSLSSYPFLMFFASLGLTSSLVIFLKYGLCLGFFFSFFFILYISFVW

*A. suum* XXX---VFHNFHILSLSSYPILIFCSSLGFTSSLVVFFKNGIFGGLLFCLFSIFLVSFAW

*A. simplex* XXX---LFHNFHILSLSSYPLLVFFSTLGFTSSLVVFFKYGLVGGVLFCLFSICYVAFLW

*T. canis* XXX---MFHNFHILSLSSYPLLVFFSSLGLTSSLVVFFKYGLSLGLLFCLFSIMFVSFAW

*S. stercoralis* XXX---FFHNFHVLSFSTYPFFMFISLSSLFSSFVVFFKFGIFSFLFFSFFSFLVLIFCW

----------------********************************************

1140

=========+=========+=========+=========+=========+=========+

***Agamermis sp.*** FGDLIRESFLKGTQNFLMQSSLKFGMIFFIVSEFFFFISFFWSYFHFMFMQSGEVGMEWP

***T. cosgrovei*** YRDLVRESLYVGSQNLLMILSIKLGMVFMILSEVFFFVSFFWTFMHFMFMYSGEMGYVWP

***S. spiculatus*** FKELNLDSFVKGMITKLMAWSFKYGMVMFIFSELMFFITFFWSYTHFMLLVMGEVGLMWP

*X. americanum* TRDVSRESVFQGNHSRLVAASLKWGLIWFLFSEVWFFFGIFWSFFHASVSPITTGNNTWP

*B. malayi* MKDVFLEDVS-GQYSFYDYRMFSQGFRLFLFSELTLFFSIFWTFLDSSLCPLTWLGGVWS

*O. volvulus* IKDVILEDIS-GQYSFYDYRMFNQGFRLFLFSELTLFVSIFWTFLDTALCPLTWLSGVWS

*D. immitis* VKDLLLEDVS-GQYSFYDYRIFAQGFRLFLFSELTLFFSIFRTFLDSALGPLTWLGGVWS

*N. americanus* GKDISMEGLS-GFDNFFVMDGFKFGVVLFIFSEFMFFFSIFWVFFDAALVPVHELGESWP

*C. oncophora* GKDISMEGLS-GYHNFFVMDGFKFGMMLFIFSEFMFFFSIFWVFFDAALVPVHELGESWS

*A. duodenale* GKDISMEGLS-GYHNFFVMDGFKFGVVLFIFSEFMFFFSIFWVFFDAALVPVHELGESWS

*A. caninum* GKDISMEGLS-GYHNFFVMDGFKFGVVLFIFSEFMFFFSIFWVFFDAALVPVHELGESWS

*B. phlebotomum* GKDISMEGLS-GYHNFFVMDGFKFGVILFIFSEFMFFFSIFWVFFDAALVPVHELGESWS

*H. contortus* GKDISMEGLS-GYHNFFVMDGFKFGVILFIFSEFMFFFSIFWVFFDAALVPVHELGESWS

*C. elegans* GKDIAMEGLS-GYHNFFVMDGFKFGVILFVFSEFMFFFCIFWTFFDAALVPVHELGETWS

*S. carpocapsae* SKDISMEGLS-GYHNFFVMDGFKFGVLLFIFSEFMFFFGIFWTFFDAALVPVHEMGESWS

*A. suum* GKDIVMEGLS-GYHNFFVMDGFKFGVLVFIFSEFMFFFGIFWTFFDAALVPAHDVGGVWS

*A. simplex* GKDIVMEGLS-GYHNFFVMDGFKFGVLVFIFSEFMFFLGIFWTFFDAALVPAHDLGGVWS

*T. canis* GKDIAMEGLS-GFHNFFVMDGFKFGVLIFIFSEFMFFLGIFWTFFDAALVPAHDLGGVWS

*S. stercoralis* FKDICMEGLC-GYHNFYVMDGFKLGFVLFIFSEFMFFFSVFWFYFDSALVPAHEIGCGWG

**********-*************************************************

1200

=========+=========+=========+=========+=========+=========+

***Agamermis sp.*** PKGVYAINCLSVPMLNSFLLLSSGVSLTVSHNMLLFEKKEF-KFLLLMTAILGVLFSYCQ

***T. cosgrovei*** PVNINSVNYMSIPLLNTLILLSSGCSLTISHMFLLMSSQQM-KSYLMYTIFLGLVFTFCQ

***S. spiculatus*** PMGINKVDFMTLALLNSVLLLSSAYFLTISHLMLVTLNKMLSKFFMIITIFLGMVFLGYQ

*X. americanum* FVGLDIIPPFQVPLLNTIVLLMSGVTATLSHHEVLA-GEK--SMWLFYSLLLGIYFLMLQ

*B. malayi* PVGILSPDYLGINGMASLFLMMNSQVLKYSRRYLCL-NVNKCENLLLLCIFIGSFFLCFQ

*O. volvulus* PLGILSPDYLGLNGMASLFLMMNSQVLKYSRRYLCL-NSSKCEEFLLVCIFIGVGFLCFQ

*D. immitis* PLGILSPDYLGLNGTASLFLMMNSQFLKYSRRYLCL-NSCKCELFLLFCIFVGSGFLCFQ

*N. americanus* PIGMHLVNPFGVPLLNTIILLSSGVSVTWAHHSLLS-NKS-CISSMVLTCILAVYFTGIQ

*C. oncophora* PIGLHLVNPFGVPLLNTIILLSSGVTVTWAHHSLLS-NKS-CTNSMILTCLLAVYFTSIQ

*A. duodenale* PIGMHLVNPFGVPLLNTIILLSSGVSVTWAHHSLLS-NKS-CTNSMVLTCILAAYFTGIQ

*A. caninum* PIGMHLVNPFGVPLLNTIILLSSGVSVTWAHHSLLS-NKS-CTNSMILTCVLAAYFTGIQ

*B. phlebotomum* PIGMHLVNPFGVPLLNTIILLSSGVSVTWAHYSLLS-NKS-CINSMILTCLLAVYFTSIQ

*H. contortus* PMGLNLVNPFGVPLLNTIILLSSGVSVTWAHHSLLS-NKS-CTNSMVLTCLLALYFTLIQ

*C. elegans* PFGMHLVNPFGVPLLNTIILLSSGVTVTWAHHSLLS-NKS-CTNSMILTCLLAAYFTGIQ

*S. carpocapsae* PMGLHLVNPFGVPLLNTIILLSSGVSVTWAHYSLLS-NKS-CTNSLILTCVLAIYFTGIQ

*A. suum* PIGMHLVNPFGVPLLNTIILLSSGVSVTWAHYSLLS-NKG-CANSLMLTCILAVYFTGIQ

*A. simplex* PIGMHLVNPFGVPLLNAIILLSSGVSVTWAHYSLLS-NKS-CSTSLALTCILAAYFTGVQ

*T. canis* PIGMHLVNPFGVPLLNTIILLSSGVSVTWAHYSLLS-NKG-CVNSLVLTCVLAAYFTGIQ

*S. stercoralis* FFGLDKINPFGVPLLNTVILLSSGVTVTWSHYCLLN-NFY-CLDGLILTIFLSFIFILVQ

************************************-----*******************

1260

=========+=========+=========+=========+=========+=========+

***Agamermis sp.*** YWEYKMLDFLWSDSYYGSIFFLGTGFHGFHVIMGSLILLTIWFRSLYFQILT-DSMMFEL

***T. cosgrovei*** SWEYWMLDFLWSNSCFGSIFYMGTGFHGIHVIIGSLILIIVFSRSLSTSISS-INNIFEL

***S. spiculatus*** MMEYMMLEFLWSDSVYGSIFYMTTSFHAFHVMVGLFMLSSVLT--LMTKMN---MMIFEF

*X. americanum* GMEYYSSLFSISSSVFGSLFFLGTGFHGFHVCLGAVMLFISFLRVNMNKLSLSHHFFLEF

*B. malayi* FFEYSHNCFVMNDSIYGSVFYIGTGLHGSHVLIGVCFLIVNFFRAKLHNFNWYHTQAYDM

*O. volvulus* FYEYNNNSFVMSDSAYGSIFYMGTGLHGLHVFVGVCFLIVNFFRVKLFNFNWYHIQAYDM

*D. immitis* FYEYSDNCFGMNDSIYGSIFYVGTGLHGFHVLVGVFFLMVNFFRIKLFNFNWYHIQAYDM

*N. americanus* LMEYSEASFSISDGIFGSIFYLSTGFHGIHVLCGGLFLGFNLFRLLKSHFNYNHHLGLEF

*C. oncophora* LMEYKEASFSISDGIFGSVFYLSTGFHGIHVLCGGLFLGFNLLRLLKSHFNYNHHLGLEF

*A. duodenale* LMEYSEASFSISDGIFGSIFYLSTGFHGIHVLCGGLFLGFNLFRLLKSHFNYNHHLGLEF

*A. caninum* LMEYSEASFSISDGIFGSIFYLSTGFHGIHVLCGGLFLGFNLFRLLKSHFNYNHHLGLEF

*B. phlebotomum* LMEYKEASFSISDGIFGSIFYLSTGFHGIHVLCGGLFLGFNLFRLMKSHFNYNHHLGLEF

*H. contortus* LMEYKEASFSISDGIFGSIFYLSTGFHGIHVLCGGLFLLFNLLRLMKYHFNYNHHLGLEF

*C. elegans* LMEYMEASFSIADGVFGSIFYLSTGFHGIHVLCGGLFLAFNFLRLLKNHFNYNHHLGLEF

*S. carpocapsae* AMEYAEASFSISDGIFGSIFYLSTGFHGMHVLFGGLFLFFNLIRLLMSHFNYNHHLGLEF

*A. suum* LMEYKEASFSISDGIFGSIFYLSTGFHGVHVLFGGLFLFFNLLRLLMSHFNYNHHLGLEF

*A. simplex* LMEYSEASFSISDGIFGSIFYLSTGFHGLHVFCGGLFLFFNLWRLLLSHFNYNHHLGLEF

*T. canis* LMEYSEASFSISDGIFGSIFYLSTGFHGVHVLCGGLFLFFNLLRLCMSHFNYNHHLGLEF

*S. stercoralis* FFEYKSSSFSISDGVYGSIFYFSTGFHGFHVILGAVFLTYNLFRFLLYHFNYSHHLGYEF

************************************************************

1320

=========+=========+=========+=========+=========+=========+

***Agamermis sp.*** GAWYWHFVDVIWMILFIEFYWWSKH-XXXX--IYVNFVNEYNKLKLKLKAPLNLSYWWNL

***T. cosgrovei*** GSWYWHFVDVVWIFLFSEFYWFSF--XXXX--------MFIKKLVFSLKSPMNLSYWWNL

***S. spiculatus*** SAWYWHFVDVIWLMLYIFYYWFPWMSXXXX----------MKSYKFSLMTPMNLSYWWNI

*X. americanum* SLWYWHFVDVVWLFLFFWVYVWNNF-XXXX---MMTSYMKGLSILWELPSPKTLSYWWGF

*B. malayi* SIDYWRFLEWMWGIMFCLLYVWGS--XXXX-----ILVGFLN-SLIFLPASFSLSYMWNF

*O. volvulus* SIDYWRFLEWMWGVMFCLLYVWGS--XXXX-------IAIFN-SLVFLPASFTLSYMWNF

*D. immitis* SIDYWRFLEWMWGIMFSLLYVWGS--XXXX-------VGVFN-SLVFLPASFSLSYMWNF

*N. americanus* AILYWHFVDVVWLFLFVFVYWWSY--XXXXIKSKSNLYNFVSSLVITLPSSKSLTINWNF

*C. oncophora* AILYWHFVDVVWLFLFVFVYWWSY--XXXXMKMKXNVLSFVNSLLVVLPSSKSLSLGWNF

*A. duodenale* AILYWHFVDVVWLFLFVFVYWWSY--XXXXIKSKNNIMNFISSLVVTLPSSKSLTIGWNF

*A. caninum* AILYWHFVDVVWLFLFVFVYWWSY--XXXXIKSKNNIMNFISSLVVTLPSSKSLTIGWNF

*B. phlebotomum* AILYWHFVDVVWLFLFVFVYWWSY--XXXXIKSKNNVINFVSSLVVTLPSSKSLTISWNF

*H. contortus* AILYWHFVDVVWLFLFVFVYW-----XXXX------VLNFVNSLVVTLPASKSLTLGWNF

*C. elegans* AILYWHFVDVVWLFLFVFVYWWSY--XXXXLKINNSLLNFVNGMLVTLPSSKTLTLSWNF

*S. carpocapsae* AIIYWHFVDVVWLFLFVFVYWWSY--XXXX--FLKGFFGFLNSLVINLPSSKTLTLYWNF

*A. suum* AIIYWHFVDVVWLFLFVFVYWWSY--XXXX-----IKLDFVNSMVVSLPSSKVLTYGWNF

*A. simplex* GIIYWHFVDVVWLFLFVFVYWWSY--XXXX----MGLFAFVNSMVVSLPSSKSLTLNWNY

*T. canis* AIIYWHFVDVVWLFLFVFVYWWSY--XXXX----LNLYGFISSLVVSLPSSKSLTLNWNF

*S. stercoralis* STLYWHFVDVVWLFLFVFVYWWGY--XXXX---------FILLSFSMFPVSFSLSVLRNF

**********************-------------------------*************

1380

=========+=========+=========+=========+=========+=========+

***Agamermis sp.*** GSFVSLIMSIQIITGLFLTFYYENS-ENCFDSIYIIHIETFYGVLVHFIHLNVSSIIFFL

***T. cosgrovei*** GSLLGLMIVVQILSGFMLTFYYENS-PYSFNSLWMIHLDMVKGYLLHYIHLNMASYVFMI

***S. spiculatus*** GSLLGVLMMVQIMTGIMLVLYYDNS-DLSFISVMMIHLEINFGYLFHIIHLGFSNYIFIA

*X. americanum* GSLLGILMAIQVASGLILAFCYSSG-FLAWSSVVEITREVYAGWLVRSIHSNTASFVFFL

*B. malayi* GSMLGIMLMSQIITGFFLTFYYTAG--DAFSSVQYIMFEVNLGWLVRIMHSNGASMFFLF

*O. volvulus* GSMLGIMLMSQILTGFFLTFYYTAG--EAFSSVQYIMFEVNLGWLLRIMHSNGASMFFLF

*D. immitis* GSMLFVMLVSQIFTGFFLTFYYTSG--GAFASVQYIMFEVNMGWFLRILHSNGASMFFLF

*N. americanus* GSMLGMVLVFQIFTGTFLAFYYTADGLMAFSAGQYIMYEVNCGWIFRIFHFNGASLFFIF

*C. oncophora* GSMLGMILVFQIFTGTFLAFYYTADGTMAFSAVQYIMYEVNYGWIFRIFHFNGASLFFVF

*A. duodenale* GSMLGMVLVFQILTGTFLAFYYTADGLMAFNAVQYIMYEVNYGWIFRIFHFNGASLFFIF

*A. caninum* GSMLGMVLAFQILTGTFLAFYYTADGSMAFNAVQYIMYEVNYGWIFRIFHFNGASLFFIF

*B. phlebotomum* GSMLGMILLFQIFTGTFLAFYYTADGSMAFSAVQYIMYEVNFGWLFRIFHFNGASLFFIF

*H. contortus* GSMLGMILVFQIFTGTFLAFYYTADSLMAFNAVQYIMYEVNYGWIFRIFHFNGASLFFIF

*C. elegans* GSMLGMVLIFQILTGTFLAFYYTPDSLMAFSTVQYIMYEVNFGWVFRIFHFNGASLFFIF

*S. carpocapsae* GSMLGMVLVFQILTGTFLAMYYSADSMLAFSSVQYIMYEVNFGWSFRIFHFNGASLFFVF

*A. suum* GSMLGMVLGFQILTGTFLAFYYSNDGALAFLSVQYIMYEVNFGWIFRVLHFNGASLFFIF

*A. simplex* GSMLGVILVFQILTGTFLAFYYTNDGASAFGSVQYIMYEVNFGWVFRLFHSNGASLFFIF

*T. canis* GSMLGMVLIFQILTGTFLAFYYCDDSMGAFASVQYVMYEVNFGWVFRVFHFNGASLFFIF

*S. stercoralis* GSILGMVLFFQIFSGLFLSFYYVADGFNFFFSVQYIMSDVNFGWVFRILHFNGASLFFFF

************************************************************

1440

=========+=========+=========+=========+=========+=========+

***Agamermis sp.*** IYLHMTKGLIFSSFSMMKITWISGWIMMMLSMLSAFMGYVLPWGQMSLWGATVITNLLSA

***T. cosgrovei*** MYLHIIKGLLYNSFSQLKYLWISGWVLMMIMMMIAFMGYVLPWGQMSLWGATVITNLISA

***S. spiculatus*** LYLHMIKGLLNNSFTKFKLLWISGCIMMVMIMMIAFLGYVLPWGQMSLWGATVITNLLSV

*X. americanum* MFMHFFRGIIQSSF-YLVLPWISGFVLMLLTMAAAFLGYVLPWGQMSFWGATVIINLLSI

*B. malayi* IYLHIFKGLINSSY-RLSSVWISGIAMYLVLMGIAFTGYVLIWGQMSYWAAVVITSLMTS

*O. volvulus* IYLHIFKGLIYGSY-RLIGVWLSGIFIYFLLMGIAFTGYVLIWGQMSYWAAVVITSLMTS

*D. immitis* IYFHIFKGLVYGSY-RLRFVWLSGIFIYFLLKGVAFTGYVLIWGQMSYWAAVVITSLMTS

*N. americanus* LYMHIFKGLFMMSY-RLKMVWISGLTIYLLVMMEAFMGYVLVWAQMSFWAAVVITSLLSV

*C. oncophora* LYLHIFKGLFMVSY-RLKKVWVSGLTIYLLVMMEAFMGYVLVWAQMSFWAAVVITSLLSV

*A. duodenale* LYLHIFKGLFMMSY-RLKMVWVSGLTIYLLVMMEAFMGYVLVWAQMSFWAAVVITSLLSV

*A. caninum* LYLHIFKGLFMMSY-RLKMVWVSGLTIYLLVMMEAFMGYVLVWAQMSFWAAVVITSLLSV

*B. phlebotomum* LYLHIFKGLFMMSY-RLKLVWISGLTIYLLLMLEAFMGYVLVWAQMSFWAAVVITSLLSV

*H. contortus* LYLHIFKGMFMMSY-RLKKVWVSGLTLYLLIMMEAFMGYVLVWAQMSFWAAVVITSLLSV

*C. elegans* LYLHIFKGLFFMSY-RLKKVWMSGLTIYLLVMMEAFMGYVLVWAQMSFWAAVVITSLLSV

*S. carpocapsae* LYLHFFKGLFFSSY-RLKNVWASGITIFLFVMMEAFMGYVLVWAQMSFWASVVITSLLSV

*A. suum* LYLHLFKGLFFMSY-RLKKVWVSGIVILLLVMMEAFMGYVLVWAQMSFWASVVITSLLSV

*A. simplex* LYLHIFKGLFFFSY-RLKKVWASGLFILLLLMAEAFMGYVLVWAQMSFWASVVITSLLSV

*T. canis* LYAHLFKGLFFVSY-RLKKVWSSGLVILLLVMMEAFMGYVLVWAQMSFWASVVITSLLSV

*S. stercoralis* LYLHFFKGLFFCSY-RLSGTWIIGLTIFFFVMMEAFMGYVLVWAQMSFWASFVITSLLSV

**************-*********************************************

1500

=========+=========+=========+=========+=========+=========+

***Agamermis sp.*** IPVIGKMMVEWIWGGYFVSNFTMKMFFSFHFLVPFIILIFIMIHLIILHYYGSNNP-IGN

12/11/2008 13:49 pasted_alignment

Page 7 of 16 http://molevol.cmima.csic.es/cgi-bin/gb_s.pl?

***T. cosgrovei*** FPIVGISLVEWVWGGYFVSNFTMKLFFSLHFLIPFILLIMILMHLFILHLFGSSSP-LGG

***S. spiculatus*** LP-YGSSLVKWVWGGYFVSNFTLKIFFCLHFLLPIVLIAIIVMHLIILHYSGSSNP-LGL

*X. americanum* LP-YGKMLVVWLWGGFYVSAATCSFFFALHYIVPFAVLVVIMVHLFFLHFSGSSSFGGMN

*B. malayi* VPYLGKYMVWWIWGSFSVCENTLKFFYSIHFIFPWLLLVMVIFHLFFLHFTGSSSS-LYC

*O. volvulus* VPYLGKYLVWWIWGSFSVCENTLKFFYSVHFILPWSLMVLVVFHLFFLHFTGSSSS-LYC

*D. immitis* VPYLGKYLVWWIWGSFSVCDNTLKFFYSIHFILPWFLLLLVVVHLFFLHFTGSSSV-IYC

*N. americanus* IPVWGSTFVMWIWSGFGVTSATLKFFFVLHFLLPWGLLVLVLGDMIFLHSTGSTSS-IYC

*C. oncophora* IPIWGATIVMWVWSGFGVTSATLKFFFVLHFLLPWGLLLLVMIHLMLLHSTGSTSL-LYC

*A. duodenale* IPIWGPSIVMWVWSGFGVTSATLKFFFVLHFLLPWGLLVLVLMHLIFLHSTGSTSS-MYC

*A. caninum* IPIWGPSIVMWVWSGFGVTSATLKFFFVLHFLLPWGLLVLVLMHLVFLHSTGSTSS-MYC

*B. phlebotomum* VPIWGPKIVMWIWSGFGVTSATLKFFFVLHFLLPWGLLVLVLMHLIFLHSTGSTSS-IYC

*H. contortus* IPIWGSTLVMWVWSGFGVTSATLKFFFVLHFLLPWGLLVLMMIHMIMLHSTGSTSI-VYC

*C. elegans* IPIWGPTIVTWIWSGFGVTGATLKFFFVLHFLLPWAILVIVLGHLIFLHSTGSTSS-LYC

*S. carpocapsae* IPVWGPSIVTWIWSGFSVSGATLKFFFVLHFLVPWFVLVLVLFHLIFLHSTGSTST-VYC

*A. suum* IPVWGFAIVTWIWSGFTVSSATLKFFFVLHFLVPWGLLLLVLLHLVFLHETGSTSK-LYC

*A. simplex* IPIWGPAIVTWIWSGFTVSSATLKFFFVVHFLLPYGLLVFVLAHLLFLHETGSTSK-LYC

*T. canis* IPVWGPSIVTWIWGGFTVSGATLKFFFVLHFLVPWGLLVIVLFHLLLLHETGSTSK-LYC

*S. stercoralis* IPYFGFSIVYWIWSGFSVVNSTPKFFFVIHFLMPWLVFLLVFVHLFFLHSTGSTSV-LYC

*******************************************************--***

1560

=========+=========+=========+=========+=========+=========+

***Agamermis sp.*** FS-LMKIEFNPIYSFKDLMSIIFIILMFVLFFYSPYFMVDPENFIKSNPMISPIHIQPEW

***T. cosgrovei*** FNMLMKEEFDLIYIWKDAVNILILMIMMILSLFFPYVFGDPENFIKASPMISPIHIQPEW

***S. spiculatus*** SSSLLKLEFAPAYVYKDLLNIIIIMLLILLTLSSPYLFSDPDNFIKSNSMVSPIHIKPEW

*X. americanum* YSDSLKVKFGLLFSLKDMVNVIFIWVGFLVILSMPDLFSDPVNFLPADLSSSPVHIQPEW

*B. malayi* HGDYDKIHFFPSYWFKDGFDIFFYFFLVLFSLYFSFNLSDPMIFVESDSMASPAHVVPEW

*O. volvulus* HGDYDKIHFFPSFWLKDGFDIFFYFFLILFSLYFSFDLSDPMIFVESDSMASPAHVVPEW

*D. immitis* HGDYDKVHFFPGYWLKDGLDILFYFFFVLFSLYFSFNLSDPMIFVESDSMVSPTHVVPEW

*N. americanus* HGDYDKICFGPVYWNKDAYNMVFWLVFVLFSLVCPFSLGDPEMFIEADPMMSPVHIVPEW

*C. oncophora* HGDYDKICFGPDYWNKDFYNVIFWICFFVFSLFNPFSLGDPEMFIEANPMMSPVHIVPEW

*A. duodenale* HGDYDKICFGPEYWNKDAYNVIFWLLFIVFSLFYPFSLGDPEMFIEADPMMSPVHIVPEW

*A. caninum* HGDYDKICFGPEYWNKDAYNVIFWLLFIVFSLFYPFNLGDPEMFIEADPMMSPVHIVPEW

*B. phlebotomum* HGDYDKVCFGPEYWNKDAYNLIFWMIFFVFSLFFPFILGDPEMFVEADPMMSPVHIVPEW

*H. contortus* HGDYDKLNFGPEYWNKDLYNLIFWIVFFMFSLFYPFVLGDPEMFIEANPMVSPVHIVPEW

*C. elegans* HGDYDKVCFSPEYLGKDAYNIVIWLLFIVLSLIYPFNLGDAEMFIEADPMMSPVHIVPEW

*S. carpocapsae* HGDYDKICFFPEYWGKDAYNLFLWLFFFIFSLCYPFIMGDPEMFIEADPMMSPVHIVPEW

*A. suum* HGDYDKVCFYPEYWVKDFLNVVVWFVFIFFSLGYPFLLGDPEMFIESDPMMSPVHIVPEW

*A. simplex* HGDYDKICFFPEFWVKDALNLVVWFVFVVFCFLAPYLLGDPEMFIESDPMMSPVQIVPEW

*T. canis* HGDYDKVCFYPEYWVKDALNLVVWLVFIGFSLVAPFYLGDPEMFIESDPMMSPVHIVPEW

*S. stercoralis* HGDYDKISFFPYYFYKDSYNLVVFFLFFIFSFFFPFLLGDPEMFVESDPMVSPVHIVPEW

************************************************************

1620

=========+=========+=========+=========+=========+=========+

***Agamermis sp.*** YFLQYYAILRAIPNKLGGVLFFLLSLILLMMMIFFNYSMMMNNMKLWNLMTIMFIMSNII

***T. cosgrovei*** YFLHYYAILRSIPNKLGAVIFFIMALMMVLSLGLLSAKFQVQSTNSWFLSVNIFVVVNMM

***S. spiculatus*** YFLQYYAILRSIPSKLGGIICFIMSVLILFMLIFLKNKQNLFSFKVVLMSWSWYVVLNLL

*X. americanum* YFLHFYAVLRAIPNKVGGLIMFFLAIFIILFFSWMSSELSLSHLFYYDFLAWSFVFLNFL

*B. malayi* YFLFAFTILRSVPNKLFGVVLMFGSIFVLLVFIWFNNYQAMMDNILY-FLSMCFVWVFFW

*O. volvulus* YFLFAFTILRSVPSKLLGVILMFSSVFVLTILIWPGSYRSILDNFLY-FFVMCFVWVFFW

*D. immitis* YFLLTFTILRSVPDKLLVVVLMFSSVFVLAILIWPGAYYSILDNFLY-FFVMCFVWIFFW

*N. americanus* YFLFAYAILRAIPNKILGVVALLMSIVIFYFFVLINNYTSCLNKLNK-FLVFIFIVVSVV

*C. oncophora* YFLFAYAILRAIPNKVLGVVALLMSIVSFYFFVLVNNYVSXMVKVNK-FLVYSFIVSSIL

*A. duodenale* YFLFAMAILRAIPNKVLGVVALLMSIVSFYFFVFVNNYTSCLVKLNK-MLVFGFIISAVI

*A. caninum* YFLFAYAILRAIPNKVLGVVALLMSIVSFYFFVFINNYTSCLVKLNK-MLVFGFIISAVI

*B. phlebotomum* YFLFAYAILRAIPNKVLGVIALLLSILVFYFFVLVNNYTSCLVKLNK-FLVFSFIVVAII

*H. contortus* YFLFAYAILRAIPNKILGVIALLMSIVIFYFFVFINNYTSCLNKMNK-MLVFLFILIASV

*C. elegans* YFLFAYAILRAIPNKVLGVIALLMSIVTFYFFALVNNYTSCLTKLNK-FLVFMFIISSTI

*S. carpocapsae* YFLFAYAILRAIPNKILGVLALLMSIVVFYFFIFFSNYHSVLDILNK-FFVFNFILISII

*A. suum* YFLFAYAILRAIPNKVLGVVSLFASILVLVVFVLVNNYVSVMSKLNK-FLVFVFIFVLVV

*A. simplex* YFLYAYAILRAIPNKVLGVVAMFGSLLLLFLFVFIHNYVSMLSKLNK-IFVFLLLFTLVM

*T. canis* YFLFAYAILRAIPDKVLGVLALFMSILVFFLFVFVDNYTSVMSKVNK-CLVWIFIFVSVV

*S. stercoralis* YFLFAYAILRAIPNKVLGVFFLVFSIFIFYFYVFFNNYYSVLDNLNF-FLVVFFILVSVF

***********************************************-************

1680

=========+=========+=========+=========+=========+=========+

***Agamermis sp.*** LVWLGGMPVEYPYLILSQILTFIYFLNFLLIYMFNYLNFILGMYFIYSEHLKSHSKYNML

***T. cosgrovei*** LMWLGGCPVEAPFLLLSKIFTIMYFINPVMLFFLVK------------------------

***S. spiculatus*** LMWLGGCPVEYPFIFLSQILSALYFIYFMLFFMLVIMINF--------------------

*X. americanum* LIWLGSQPVEDPFIWLSQLMTLLYFSFFFLLKSLDFLTSFFF------------------

*B. malayi* LTWAGHYPTDYPFSYFNLICTIFYFIFIFFMCFVNFLSFKLFS-----------------

*O. volvulus* LTWAGHYPTDYPFNYFNLFCTFFYFCCIFFVCLINFFSDKLFS-----------------

*D. immitis* LTWAGYYPTDYPFNYFNLFCTFFYFCFIFFICLVNYVGCKIFS-----------------

*N. americanus* LSWLGQCLVEDPFTMLSPLFSLLYFLLILLLLFVFKFSKNLFI-----------------

*C. oncophora* LSWLGQCLVEEPYTMLSPLFSFIYFFLIFVLFLFYSKSKNLFM-----------------

*A. duodenale* LSWLGQCLVEEPFTILSPLFSFIYFFLILLMLLVFYFSKKLFI-----------------

*A. caninum* LSWLGQCLVEEPFTLLSPLFSFIYFFLILLMLLVFYFSKKLFI-----------------

*B. phlebotomum* LSWLGQCLVEDPFTYLSPLFSFFYFFFVVLIFLMFYFSKKLFV-----------------

*H. contortus* LSWLGQCVVENPYVLMSFIFSFLYFMIIIMMMX---------------------------

*C. elegans* LSWLGQCTVEDPFTILSPLFSFIYFGLAYLMLFIFMSSKLLFK-----------------

*S. carpocapsae* LSWLGQCMVEYPFTFLSIVFSILYFFVIFILLFIYILEIFIFS-----------------

*A. suum* LSWLGQCLVEDPFVFLSMVFSFLYFFVIFLLFLVYYFAGRVFM-----------------

*A. simplex* LSWLGQCLVEEPFITLSMVFSAIYFVLVLTLLFIFFITKFIFK-----------------

*T. canis* LSWLGQCLVEAPFVFLSMFFSFMYFFVVVVMVFLFMFFGKLFS-----------------

*S. stercoralis* LSWLGQCHVEYPFINLSVVFSFLYFFFAFLIVFSRFLTKFIFS-----------------

*************************-----------------------------------

1740

=========+=========+=========+=========+=========+=========+

***Agamermis sp.*** MILXXXXLYVNLKWFKLKMSIMAKMKLMLLLTVIFILLSVAFFTLTERKFLSYSQLRLGP

***T. cosgrovei*** ---XXXX---------MMIKGAKNNHANISKLSMRILISIAFFTLLERKFLGSTQLRFGP

***S. spiculatus*** ---XXXX-------------------MQMIISIILIMISIGFFTLLERKLLSLSQIRLGP

12/11/2008 13:49 pasted_alignment

Page 8 of 16 http://molevol.cmima.csic.es/cgi-bin/gb_s.pl?

*X. americanum* ---XXXX--------------MMLWMASTLILLILGLGLVAFFTLLERKLLGFSQIRVGP

*B. malayi* ---XXXX-------------LFIFYYIGIFLMIIFILQAIAFLTLMERHFLGGTQCRIGP

*O. volvulus* ---XXXX-------------LVIFYYLGLLVMIVFILQAIAFLVLLERHFLGGSQCRVGP

*D. immitis* ---XXXX-------------LFFLFYFGFLIMIVFILQSVAFLTLLERHFLGGSQCRIGP

*N. americanus* ---XXXX--------------IFLSFLMIILMMFFILQAIAFITLYERHLLGSSQNRLGP

*C. oncophora* ---XXXX--------------LLLMVLLILLMMIFILQGVAFITLYERHLLGGSQNRLGP

*A. duodenale* ---XXXX--------------ILLSFLMIILMIVFILQAIAFITLYERHLLGSSQNRLGP

*A. caninum* ---XXXX--------------ILLSFLMIILIVVFILQAIAFITLYERHLLGSSQNRLGP

*B. phlebotomum* ---XXXX--------------VVLSFLLVFLMMIFILQAIAFVTLYERHLLGSSQNRLGP

*H. contortus* ---XXXX-------------------------MVFILQAVAFITLYERHLLGSSQSRLGP

*C. elegans* ---XXXX--------------MILVLLMVILMMIFIVQSIAFITLYERHLLGSSQNRLGP

*S. carpocapsae* ---XXXX--------------FFFLLIQVILMVFFIVQGIAFITLYERHLLGSSQNRLGP

*A. suum* ---XXXX--------------LILMLVQVILIMIFVIQSIAFVTLYERHLLGGSQQRIGP

*A. simplex* ---XXXX--------------LVLLLLEVILVMLFVVQAIAFVTLYERHLLGGSQQRIGP

*T. canis* ---XXXX--------------LIVMLVEVILVMLFVVQAIAFITLYERHLLGVIQAIFDG

*S. stercoralis* ---XXXX-------------IFLFMILLVFFVVFFVMQSIAFITLYERHLLGLSQSRLGP

----------------------------------**************************

1800

=========+=========+=========+=========+=========+=========+

***Agamermis sp.*** NKMFFNGLFQPFLDGVKLFKKNSLINLKNHYILYKIMSFMILLVSLIMWIMIP-FSSWSN

***T. cosgrovei*** NKVSFWGWIQPILDGIKLLKKTNILNLKIYTTIYFFMSFAILILALLIWAVMP-FYLWNL

***S. spiculatus*** NKTLIIGMIQPILDGLKLIKKGIIFNKKIYIIMFNMGAFFLLMLAIFLWMSFP-FLMWIN

*X. americanum* NKLAFSGLLQPVMDGLKLLTKNMYMPVITQMLLFIG-PIISFMVFMIFWVLVLPWNGNFM

*B. malayi* NKVGYSGIFQALFDGLKLLKKEQLFFYFSSWLSFLLMPVCSFILMIFFWFTLPYFFVFMS

*O. volvulus* NKVGYCGVLQALFDGLKLLKKEQLLLCFSSWLSFLFMPICGFVLMVFFWFTLPYFFSFLS

*D. immitis* NKVAYCGFFQAFFDGLKLLKKEQLVFFCSSWISFLFVPVCGFVLMIFFWFTLPYFFSFLT

*N. americanus* TKVSFMGVLQALLDGVKLLKKEQMMPLNSSDLSFLLVPGVSFLVMYLEWFVLPYFFEFLS

*C. oncophora* TKVTFMGVLQALLDGVKLLKKEQLLPIYSSDLSFLFVPGVAFVIMYLEWFVLPYVFNVLN

*A. duodenale* TKVSFMGVLQALLDGVKLLKKEQMTPLNSSDLSFLLVPGVSFVVMYLEWFILPYFFNFLS

*A. caninum* TKVSFMGVLQALLDGVKLLKKEQMTPLNSSDLSFLLVPGVSFVVMYLEWFILPYFFNFMS

*B. phlebotomum* TKVSFMGVLQALLDGVKLLKKEQLTPLNSSDLSFLLVPGVSFVVMYLEWFVLPYFYNFLS

*H. contortus* TKVSFMAVLQALLDGVKLLKKEQLLPVLSSDMMFLLIPGLSFVVMYLEWCVLPFFYSFVS

*C. elegans* TKVTFMGLAQALLDGVKLLKKEQMTPLNSSEVSFLLVPGISFVVMYLEWFTLPYFFDFIS

*S. carpocapsae* TKVSFMGVLQAIMDGFKLLKKEQITPFYSSDISFLLVPGISFVVMYLEWFTLPYFFSFFS

*A. suum* NKVSFMGFLQAIFDGVKLLKKEQMTPLNSSEISFILVPGIFFIVMYLEWFVLPFFYDFMT

*A. simplex* NKVSFMGIVQAIFDGIKLLKKEQMTPLNSAETSFILVPGVFFSVMFLEWFVLPYSFDFMT

*T. canis* IKLLKKEQMTPLNSSEVSFILVPGVFFLVMYLEWFVLPYAFDFITFEYSVLFFLCLIGFS

*S. stercoralis* TKVGFFGVMQAIVDGVKLLSKEQFLPYNSSYFFFLLETTVTFCLMYLEWFVLPFFFDFFT

************************************************************

1860

=========+=========+=========+=========+=========+=========+

***Agamermis sp.*** KIMLPLFIILMFGISSYMMLLLGWSSMNNYSLMGGSRSLAQVLSFEMLITLLVLITFVLK

***T. cosgrovei*** KMLVLMWILMVLGLSAFMILMIGWSSLSKFALLGSNRSISQVLSFEVNFTMLIFLPFVMK

***S. spiculatus*** KSMVMFWILLLLGLSTYMLFLIGWSSLSKFSFLGSMRALAQTISLEVLFSMLMFLGFSLM

*X. americanum* FRCSALLMFLMLGFSAYSVVIMGWGITSMFSKLGSLRAMLQSLSFEVSLILAFFMTLIHM

*B. malayi* FDYSGVFLFCLMGVSAYFIMLSGIFSGGKYSFIGGLRACAQSYSYEIAFSVYLLIFLLFN

*O. volvulus* FEYSGVFLFCLMGVSVYFIMLSGVFSGSKYSLIGGLRACVQSYSYEIAFSIYLLVFLLFN

*D. immitis* FEYSGVFLFCLMGVSVYFIMLSGIFSGGKYSFLGGIRSCAQSYSYEIAFSVYLLVFFLFN

*N. americanus* FEYSMVFFLCLIGFSVYATLISGIVSKSKYGIIGALRVSSQSISYEIAFSLYLLAIMLHF

*C. oncophora* FEYAVLFFLCLIGFSVYAMLISGLVSKSKYGIVGALRASSQSVSYEIAFSLYILSIVIHY

*A. duodenale* FEYSLLFFLCLIGFSVYTTLVSGIISKSKYGIVGALRASSQSISYEIAFSLYLLAVMIHY

*A. caninum* FEYSLLFFLCLIGFSVYTTLISGVVSKSKYGIVGALRASSQSISYEIAFSLYLLAIMIHY

*B. phlebotomum* FEYSVLFFLCLLGFSVYSTLISGIVSKSKYGIIGALRASSQSISYEIAFSLYLLSIMLYY

*H. contortus* FEYVILFFLCLIGFAVYTTMISGVVSKSKYGMIGALRASSQSISYEIAFSLYVLSVLVHN

*C. elegans* FEYSVLFFLCLIGFSVYTTLISGIVSKSKYGMIGAIRASSQSISYEIAFSLYVLCIIIHN

*S. carpocapsae* FEFSVLFFLCLVGFSVYTTLISGVVSKSKYGLIGAIRASSQSVSYEIAFSLYLLCIMIHN

*A. suum* FEYSILFFLCLIGFSVYTTLVSGMVSKSKYGMVGAIRASSQSVSYEIAFSLYLLAIVMHI

*A. simplex* FEYSILFFLCLIGFSVYTTLVSGAVSKSKYGMVGAIRASSQSVSYEIAFSLYLLAIIIHI

*T. canis* VYTTLVSGVVSKSKYGSIGAIXAXXQXVSYEIAFSLYLLAVIVHVNMFCFSSVFNLSLLV

*S. stercoralis* LNYSVLFFLCLIGFSVYTTLISGIVSKSKYSMLGSIRASSQSISYEISFTFFVFCVVLHV

************************************************************

1920

=========+=========+=========+=========+=========+=========+

***Agamermis sp.*** SSMSLKILDY---KSPMLFFFILICFS-ISIILEAHRAPLDLAEGESELVSGYNTEFSSL

***T. cosgrovei*** CSMTYASFLS---MSLNFLTILLTG---LTLVMEVQRAPADLSEGESELVSGYNTEYSSV

***S. spiculatus*** SKYN-TTYYN---QSNNLVQVTLTFYFFITILIEAHRAPMDLSESESELVSGYNTEFSSV

*X. americanum* NSVNLSNFLT-----CFEPSITWVFMWIILCLMESNRAPFDLLEGESELISGFNIEMSSV

*B. malayi* KSLCLSFSFC-------LFFFFIFLPFFCLVLIDLHRAPFDLSECESELVSGFNVEYSSV

*O. volvulus* KGLCLSFSFC-------LFFFLFFFPFFCLVLVDLHRAPFDLSECESELVSGFNVEYSGV

*D. immitis* KSLCLFFSFS-------FFFFCFLFPFSCLVLVDLHRAPFDFSECESELVSGYNVDYSSM

*N. americanus* SVFFFVSSFC-------ISLLVIYVPFLIMIIAELNRAPFDFAEGESELVSGYNVEYASV

*C. oncophora* SVFVFFSFNS-------LSLLVLYLPVFVMIVAELNRAPFDFAEGESELVSGYNVEYGSV

*A. duodenale* SMFTFVSDLI-------LSLLIIYLPFLIMIIAELNRAPFDFAEGESELVSGYNVEYASV

*A. caninum* SMFTFVSGFT-------LSLLIIYLPFLIMIIAELNRAPFDFAEGESELVSGYNVEYASV

*B. phlebotomum* SVFSFCSEFS-------VSLMVIYIPFLVMIIAELNRAPFDFAEGESELVSGYNVEYASV

*H. contortus* KFFSFMSQYL-------IVLFIIWLPFLVMIIAELNRAPFDFAEGESELVSGFNVEYGSV

*C. elegans* NVFNFVSKFN-------LSLLIIYIPFLIMVIAELNRAPFDFSEGESELVSGFNVEFASV

*S. carpocapsae* SMFFFHSVFS-------LSFFVIYIPFLLMVLAELNRAPFDFAEGESELVSGYNVEFSSV

*A. suum* NMFCFFSFFN-------LSLFIVYLPFLFMVLAELNRAPFDFAEGESELVSGYNVEYSSV

*A. simplex* NMFYFYSFFN-------LSLVVIYLPFLFMVLAELNRAPFDFAEGESELVSGYNVEYSSV

*T. canis* IYLPFLFMVLAELNRAPFDFAEGESELVSGYNVEYSSVAFVLLFLGEYGALLFFSTLTSV

*S. stercoralis* GVFSFFSFFS-------FSMMALFFPFLIMIIGKLNRAPLDFSEGESELVSGYNIEYSSV

********* *******************************************

1980

=========+=========+=========+=========+=========+=========+

***Agamermis sp.*** YFILIFLSEYSSLILMCMLFFVILWNNSILLLVLMLIIIIIRKLLSSNSVWSSDKNYVIK

***T. cosgrovei*** LFTSIFLAEYSNMMSLNMLMLVLFFNKVYVSVFMIFITLMLLVRACYH--EFSKTMKIIM

***S. spiculatus*** TFVMIFLSEYSNMIMFMGLAMFLWL-KQLILYWLVLLTCILMVRSCYPRIRYDHLMNLCW

*X. americanum* LFVLVFLSEYGILFCLGMLVSIPFSEGL-SGTALFCTSFMLFVRSCFPRVRYDSLMVLMW

*B. malayi* GFASLFLGEYGNLLYFSCLTSSLFFGMS-FFFFYVVVCLIIFCRSAYPRFRFDVLMYICW

*O. volvulus* GFAALFLGEYGNLLYFGCLTSSLFFGMS-FFFFYFIVCMIVFSRSAYPRFRFDKLMGVCW

*D. immitis* GFAFLFLGEYGNLLYFNCLVSSLFFDMS-FFFFYFFVCFIIFSRSSYPRFRFDMLMSLCW

*N. americanus* AFVLLFLSEYGSLIFFSIMSAMLFFKFS-MLMGFLIFSLLIFIRSSYPRFRYDMMMMMFW

*C. oncophora* AFVLLFLSEYGSLIFFSVLCSVLFFNFS-MLMSFVIFSLLIFIRSSYPRFRYDKMMSSFW

*A. duodenale* AFVLLFLSEYGSLIFFSVMSSMLFFNFS-MLMSFLVFSIMIFIRSSYPRFRYDMMMMMFW

*A. caninum* AFVLLFLSEYGSLIFFSVMSSMLFFDFF-YSYKFFNFSIMIFIRSSYPRFRYDMMMMMFW

*B. phlebotomum* AFVLLFLSEYGSLIFFSVMSSMLFFNFS-MMFSFIVFSLLIFIRSSYPRFRYDMMMLMFW

*H. contortus* AFVLLFLSEYGSLIFFAVFMSMMFFNFS-LVMGFVIFSLFIYIRSAYPRFRYDMMMMMFW

*C. elegans* AFVLLFLSEYGSLIFFSVLSSAMFFKFS-IFMAFSIFSLLIFIRSSYPRYRYDLMMSLFW

*S. carpocapsae* AFVLLFLSEYGSLIFFSTISSVLFFNFS-FFMIFFLFSILIFIRSAYPRYRYDFMMSFFW

*A. suum* AFVLLFLGEYGALLFFSTLTSVLFFGFS-YVVIYCMFTILVFVRSSYPRFRYDLMMYFFW

*A. simplex* AFVLLFLGEYGALLFFSTLTSVLFFDFS-FVMIYLMFTTLVFIRSAFPRFRYDLMMSFFW

*T. canis* LFFSFSFLAVYLMFTLLVFIRSAYPRFRYDLMMSFFWFKLLPVSLIFLGYFVAVFL----

*S. stercoralis* AFVLLFLSEYGVLIFFCVLFSFLFFSGS-VFMSFVIFSLLIFIRSSFPRYRYDMMMGFFW

****************************--------************************

2040

=========+=========+=========+=========+=========+=========+

***Agamermis sp.*** TNTIFNYIMIFMFNIMIFNKFN-----XXXX-------IKLLIYFLIMIFSLNIYLWWWT

***T. cosgrovei*** EDIVQKDIVQKSE--ICFKS-------XXXX-MIMFYKSLLCVYMLILIMSLNFTTWWWL

***S. spiculatus*** KNMFFSLIMLMIL--FYPMKFI-----XXXX---------MQFYILFMLVSLNINSWWWV

*X. americanum* QAFLPV-VVMLSFQLKFF---------XXXX---MYMIFLLLGYSFVMVLGLNQTSYILI

*B. malayi* FLFLPIGFYFFGFSFVVFML-------XXXX-LLFFVFFFMLFLSFINFCVLDYIVWWSV

*O. volvulus* FLFLPIGFYFFGLSFVVFMLCLFSLIFXXXXILLFFVFFLLLFLSFINFCVVDYIVWWSI

*D. immitis* FVFLPFGFYFFGLSFVVFLFCLFSLIFXXXXIVVFFVFFVMVFLSCVNFCDVDYIIWWSV

*N. americanus* FKFLPVSLIYLVYFYLLFI--------XXXX-LYLFLMVFVLFLSLLGLLVNNVLVWWSI

*C. oncophora* FKFLPISLIYLSFFYVLFF--------XXXX-LLAFMFMMVFFFGLMCLMXNNILIWWSV

*A. duodenale* FKFLPIWLIYLMFFYVLFI--------XXXX-LLLFLMMFVIFLSLLSLLVNNIFVWWSV

*A. caninum* FKFLPISLIYLMFFYVLFI--------XXXX-LVLFLMMFVIFLSLLSLLVNNVFVWWSI

*B. phlebotomum* FKFLPISLIFLFYFFVLYV--------XXXXIFVIFFYVFCFFLSILSLMVNNVLVWWSV

*H. contortus* FKFLPLSLIFL----------------XXXX-----MSVFVLFLCLMCMLVNNVMMWWSI

*C. elegans* FKLLPISLIMLCFYAVIFYY-------XXXX-LIVFISLFTLFLTLLSILTNNVIVWWSI

*S. carpocapsae* FKLLPVSLILLGFFVILFF--------XXXX-LIIFLSFSVFFIFFVNLFTSNILVWWSG

*A. suum* FKLLPVSLIFLGYFVIFLF--------XXXX-LLLFFCIFVVFLCVLNFFTSNVLVWWSV

*A. simplex* FKLLPVSLIFLGYFIIVLL--------XXXX-LLLFFCFFVSLLCFLNFFSSNVLVWWSV

*T. canis* ---------------------------XXXX-ILVFWVVFVVLLSFLNFFSSNVLVWWSV

*S. stercoralis* FIVLPISMILFLFFCLLFF--------XXXX---FFFYFFVIFVFFLNCFICNIVFWWSI

----------------------------------------------------********

2100

=========+=========+=========+=========+=========+=========+

***Agamermis sp.*** WLMVELLNWFLMLMMK-SNYILLFLMWQSFSSVMIM--FSVFYMNNMILLFILLKMGMPP

***T. cosgrovei*** WMSFELLNWVLLLFL-ETNQALIFLIWQSLASIFFLYMLVLVKNWGMIQWILLIKMSVPP

***S. spiculatus*** WLMLEVLNWTFIYFLKNMNLLIVFFMWQSISSIIFLYSFLFFKKKLLIFISLLMKTSLPP

*X. americanum* FLVLEWLSWVFSITL----TTLSMKYLLIQSYFSLVFLIIIFMAPELIIFSLLLKMGFPP

*B. malayi* FVICTFVFVFLTIG--GGSTSCLVNYYVIQEVCGYY--FLVFDSWKFQFFFLMLKSGSAP

*O. volvulus* FVICTFVFVFLVGG-ELGFSSCLVNYYVIQEVCGYY--FLVFDGWKLQFLLLMLKSGSSP

*D. immitis* FVVCTFVFIFFVCNNSLDNLGCMVNYYVVQEICGYY--FLLFDSWKLQFLFLMLKSGSSP

*N. americanus* FLLMTLVFIMLNKGL--KSYSGLFIYFVIQESLGLL--FLMFSFGLFQLLILMLKIGMAP

*C. oncophora* FLMMTLLFVMLNKMN--MSFNSLFNYFVLXESLGLL--FLLL-MGNMQLMVLFLKVGVAP

*A. duodenale* FLLMTLVFVILNKKV--NSYSTLFNYFVMQESLGLL--FLMFSFSYFQLIILMFKIGMAP

*A. caninum* FLLMTLVFVMLNKKV--NSYSTLFNYFVMQESLGLL--FLMFSFSYFQLIVLMFKIGMAP

*B. phlebotomum* FLLMTLIFVMLNKKI--NSYASLFNYFIIQESLGLL--FLMFSFYYFQLLIIFLKIGMAP

*H. contortus* FLLMTLMFVTINKKM--ESYSTMMNYFIMQESLGLM--FLLLTSYYMQLMILMMKIGVAP

*C. elegans* FLLMTVVFILLNKSS--KSYTSIFNYFVIQESLGLL--FLLCSGGLLQFFIILLKIGVAP

*S. carpocapsae* FLLMTIIFVFINKNL--YCYSGLLNYFIIQEVLGLF--FIVSFMEGFQFFFLLMKVGVSP

*A. suum* FLLMTVVFVCLSKGS--GSYVGILNYFVIQESLGLF--FLVFNVFLLQFFIVMMKVGVAP

*A. simplex* FLLMTVVFVCLSKGL--GSYASILNYFVIQESLGLF--FLVFNFFLLQFFIVMLKVGVAP

*T. canis* FLLMTVTFVCLSKSI--GAYTSVLNYFVIQESLGLL--FLVFNTFLLQFFVVMMKIGVAP

*S. stercoralis* FFLMTLVFVFLNFN---NSYSS-INYFIFQEFLGLL--FVFFSFGVFQFFIILLKVGVSP

*************-----******************--**********************

2160

=========+=========+=========+=========+=========+=========+

***Agamermis sp.*** FHHLIIN---SQFNWSFISFIVFNTIHKYLPCLILMNFMYMNLMFLMLIFLGILFFLFNF

***T. cosgrovei*** FHFMFYSFLLKMKMKGFMIFISIHKIIPTLFILNIFKMQWFVLLTNG--LSFLSFFFSNS

***S. spiculatus*** FHLWMWMLLSVMDWKSMVLLMCVHKYTPILIMMNNFSYLNCFYFIIFSLISFFNMWMASS

*X. americanum* FYNWAITLFKFFSKSSFLFVMTLHKVLPTVFSSKCFS---VYFGLLSIVMLCALFLQATD

*B. malayi* FHFWLFSVLSNLKGWSILWFLTLQKLPYFVVLLNFCSDFFFLFLFFGMIFCYVHFFLLRN

*O. volvulus* FHFWLFSVLGGLNKWFVLWFLTLQKLPYFVVLVNFCGDFFFLFLFFGMIFCYFQFFLLRS

*D. immitis* FHFWVFSVLGGLKKWFVLWFLTLQKLPYFVVLINFCSDFFFFFLFFGMVVCYLQFFLLRN

*N. americanus* FHFWVFSVTNSVFGFNLMWFLTFQKLPFLLIFLQLMFGKLVLLLFFGIVFCMFQMLLMKS

*C. oncophora* LHFWIFSVTNSVFGFNLMWFLTFQKLPFLFVIVQMVEVKFLFFLFFGLMLCLLQMLLVKS

*A. duodenale* FHFWIFSVTNSVFGFNLMWFLTFQKLPFLLIFLQMMVNSLIYLLMIGLFFCLFQMLLVKT

*A. caninum* FHFWIFSVTNSVFGFNLMWFLTFQKLPFLLIFLQMMTGSLVFLLLVGLFFCLFQMLLVKT

*B. phlebotomum* FHFWIFSITNSVFGYNLVWFLTFQKLPFLVILLQFIFGKMIFLLFIGLFFCLFQMLLMKS

*H. contortus* LHFWIFSVTNNVNGMNLMWFLTFQKLPFFFVLLQMLINYMLMFLLMGVLLCMIQMLVTKS

*C. elegans* LHFWIFNVTNNIFNYGLMWFLTFQKLPFLTILLQIFWLSSVYILLFGLLICYVQIFVMKS

*S. carpocapsae* LHFWVFNVLEGVYGWGIMWFLTFQKLPFLPVLLVLVDFXFFCFIVLGIFFCYFQMLILKD

*A. suum* FHFWVFSVTGSLYDWLLMWFLTFQKLPFLPVLVQLFDFSAFFIFLFGICVCYFQLFVLKG

*A. simplex* LHFWLFSVTNSIYDWLLMWFLTFQKLPFLPVLVQLFDFKMVYLFFFGIAVCYLQLFMLKS

*T. canis* LHFWVFSVTNSLGGWLLMWFLTFQKLPFLPVLVQLYDFKVVFLFLFGIFVCYLQLFVLKS

*S. stercoralis* FHFWCLNIFNGLSGFSTFWFLTFQKLPYFPVFSFIFDYSFFFFLVFGIIFCYFNLFFCKS

**************************--------------------**************

2220

=========+=========+=========+=========+=========+=========+

***Agamermis sp.*** SLKLFILGMSIVDMFWMISSWLISMKIFTIYLLMNLFILLMWLNSWKTSNINFYSFNIQI

***T. cosgrovei*** FKMMFFYLV-SGDGAWILMLWSSHQMVYLIYFLYFSLLVYLVNDWHNQNK--------LL

***S. spiculatus*** LKKIFWVIL-LNDTVWLMLSFYCMLNMMFIYMMFNFIIMFIFMNWKLKFS------GYLM

*X. americanum* LFLVLMFSSFLHGWWLILAGYSYQKLFWQYWMVYGSVLSLFLMSIAFSSLSLFSNGQTTL

*B. malayi* FLDMVVIGS-TESFNWLLLLGMFSFNEVFVLFFFYYFVMFLIISYIYNSG--INFISLEM

*O. volvulus* YRDLLVVGS-AESFNWLLLLGIFSFNEVFVLFFFYYFVMFFVISYVYGGF--LGFLSLEM

*D. immitis* YSDVVIIGS-VESFNWLLLLGIFSFNEGFVFFFFYYVTMFFVISYVYSGF--LSFFSLEM

*N. americanus* YKNLLILSS-TESFNWVTLGLVFSFLNVFFLFLYYFVLMLLFIPKFEIYNF-FSYLGWET

*C. oncophora* FKNLLILSS-TESFNWILLSYVFSILNVIFIFLYYFFVMXYLIPKFTMN-V-NEFVDWET

*A. duodenale* YKNLLVLSS-TESFNWVTLGFLMSFLNVLLIFFYYFILMVIIIPKFEVFNV-LNFVGWET

*A. caninum* YKNLLILSS-TESFNWITLGFLMSFLNVLFIFVYYFVLMIMVIPKFEVFNV-VNFVGWET

*B. phlebotomum* YKNLLILSS-TESFNWIVLGFLMSFFNVFVIFFYYFFVMLFLIPKFEFLNV-VGYLGWET

*H. contortus* FKNLMILSS-TESFNWILMSWIFMFVNSIYVFLYYLIFMLYLINNYSKNEK-SKYFSWET

*C. elegans* YKNLLIISS-TESFNWIVLGVFFSMFNTFYLFIYYFVLMVLLISKFSKTSG-YNFINWET

*S. carpocapsae* YKKLIIISS-TESFSWLILVGYFNFFNMYFLFFYYFFLMCFMLPYLNNFY--CNFLNWEL

*A. suum* YKSMMVISS-TESFNWVVLTCFLSVVNVIYLFFYYVVLMAFLMPNFNVKD--FNFVNWEV

*A. simplex* YKNMMIISS-TESFNWVILTSFLSVINVVYLFFYYIVLMIFLMPLFNSKD--LNFINWET

*T. canis* YKNMMVISS-TESFNWVVLTCFLSVVNVLYLFFYYLCLMVMLMPSFVVKD--LSFVNWET

*S. stercoralis* FKSMVFISS-VESFNWLLISLFSSLFSGFFLFLYYVFVMFFLLDYSSSKG--YDFYNWEL

*********-*****************************---------------------

2280

=========+=========+=========+=========+=========+=========+

***Agamermis sp.*** ILIFLCSIPPLSSFVMKWLIVGFLNFN---MGLVSLMGSWFIMILMWMWLSLQLINKELI

***T. cosgrovei*** MVVMIMGFPPMIMFNLKWMILIHLNLTLSLALMMTILLNIVYF-WSFWVIGLNFKLLKTI

***S. spiculatus*** MLLFVTSLPPLPTFQLKWLILSSLSINMMVVLIVNLLMLLFFMILLFMTLKMHKMFKDSL

*X. americanum* SGISFLVLSGFPPFLLFWMKVSVFIILMKSSVYLAFMLIFSSVVSLFVYFRVLCLSLSLS

*B. malayi* LMIFFNVPLSLTFFLKVLLLFGTSSMVGFFYFFLLLFMCLMSLGVSYLFFCVSMIGFNFG

*O. volvulus* LMFFFNVPLSITFFLKVIVLFGSSFFVGFYYLFLLLFMPLMSLGVGYLFFLVSMMSFNCG

*D. immitis* LMVFFNVPLSITFFLKVLLLFGSGFFVGFYYYFLLLIMPLMSLSIGYLFFLISMSGFNQG

*N. americanus* VLVFMNLPFSVNFFVKIFSLSEIFKVYSFIFLLLLFMMFFSVLSLSFWMVNLSTKLNVNV

*C. oncophora* VLVFLNLPFSVNFFVKIFALVNIVSWMNFWILFLLFLMFMSMVSLSFWLVSLSVKSFNSN

*A. duodenale* MLIFMNLPFSVNFFVKIFSLSEIFKVYSIGVLLLLFMMFFSVLSLSFWMVNLSTKFLYIF

*A. caninum* MLIFMNLPFSVNFFVKIFSLSEIFKIYSIWVLLLLFMMFFSVLSLSFWMVNLSTKFSQIF

*B. phlebotomum* MLVFLNLPFSVNFFVKIFSLSEVLKVYGVLVLFLLFLMFFSVLSLSFWLVNLSSKFFFDL

*H. contortus* VLVFMNLPLTVNFFVKIYALMNVVLNINIYIMILLFFMFMTILSMSFWMINLSVKNINNN

*C. elegans* TLVFLNIPFSVSFFVKIFSLSEIFKYDSFFTLFLLFTMFLSVLAFSFWLINLSMKNNEET

*S. carpocapsae* VLVFLNMPFTFTFFVKIFSLSFIFFYNSFLFLFILFLMFLSMISFSFFLVNLSVMNFSNI

*A. suum* LLVFLNVPFSVSFFIKIFVLSEVFKLDGLFLLFLLLMMFLSMLCFSLWLVNMSVKNMKML

*A. simplex* LLVFLNVPFSVSFFIKIFSLGELLKLDSMFMLFLLFLMFLSMMCFSLWLVNMSVKHMKMV

*T. canis* VFVFLNVPFSVSFFIKIFSLSELFKLDGVFVLFLLFVMFLSMLCFSLWLVNMSVKTMWFL

*S. stercoralis* VFLFMNFPMTITFFIKIFSIIFFFNISSFYLLFVLFLMVFSVFGFGFWFFNVGLKFSYYG

-------------------------------------------------***********

2340

=========+=========+=========+=========+=========+=========+

***Agamermis sp.*** FESKDINMMMIMLMMLWLL--------------------XXXX---MVNIIVNLLIMITI

***T. cosgrovei*** SKNLTFLGVLNLLAFISTQ--------------------XXXX----IVKILSFLLILVL

***S. spiculatus*** LSLYYMSFSMGFWFFFM----------------------XXXXLWNLIQQIFVLLLIMLL

*X. americanum* DKNNMKFIPVLTISLLVGFF-------------------XXXX------MVVILFVLVIL

*B. malayi* LKYYDYFFYLLFCIGLLSVF-------------------XXXX-LFLNLIIVFFFSFLVP

*O. volvulus* FKYYDYFVYVLFCIGLLSCF-------------------XXXX-LFLNFIVIFFFSFLVP

*D. immitis* LKYYDFFVWILLCIGFLSYF-------------------XXXX-LFLDFFVVFLFSFFVS

*N. americanus* -KYNNFMFLFFLPLTLIILL-------------------XXXX--ILSLLLVVFFALFLL

*C. oncophora* -KYSK-MYFMFLPLMMIILV-------------------XXXX--IVVLFWVVLITLVLL

*A. duodenale* -KYNKGMFMFFLPMTLISFN-------------------XXXX--ILNLLLVIFFALFLL

*A. caninum* -KYNKGMFMFFLPMTLVILL-------------------XXXX--ILNLLLVIFFALFLL

*B. phlebotomum* -KYNKGLFFLVVPFTVVLLI-------------------XXXX--ILNLLLVVFLSLVLL

*H. contortus* -EYNKFIYLVIMPLMTIMMI-------------------XXXX--LVQLLMVVFITFFLL

*C. elegans* SNNNKMNYFIIFPLMVISII-------------------XXXX--ILVLLMVLVFTLVLL

*S. carpocapsae* QYSLKYNYFFLIPFIFLILI-------------------XXXX--FFILSFVFFISIFFL

*A. suum* GDNFKVLFFLVFPMMVFSVIYYFSKILLCRLDKAEFFLKXXXX--LLVLVMVVLFTLVLL

*A. simplex* GDNVKNLFFLVVPIMMLSVV-------------------XXXX--LMVLVMVIIFTLLLL

*T. canis* SNNLKSVFFFCCPHDGDFCNLLS----------------XXXX----VLVMVIVFTLLLL

*S. stercoralis* FYNFSFFYFVFFPLMFFCLF-------------------XXXX--FFVLFLVTLFSFLFV

---------------------------------------------------*********

2400

=========+=========+=========+=========+=========+=========+

***Agamermis sp.*** IFMIHFLIFDYSKFFMEY--KPFECGFENMYWLHQKLNIHFFKIGIVFILFDLELLLLVL

***T. cosgrovei*** LIMLNFMLS-LFKSNVNS-LKIFECGFEIFYWSKPKTSIHFFKVGLIFILFDLEFLFLLL

***S. spiculatus*** VLVLFYILNFLLKINSNSMLSIYECGFDCVYWVHNKMNLHFFKMLLIFIIFDLELMLLVF

*X. americanum* IFFLLQLILSDYSLFTQDMLSPLESGFESLKTS-SLFGSYFFLMAVLFVLFDMELILILP

*B. malayi* FFMYFFSMVFSYKFVDFGKLSSYECGFDLSLSVRDSFSVVFFLIVLIFVVFELEVIIFII

*O. volvulus* FGMYLLSFFVSFKDFYGAKLSSYECGFDVVKKVHVGFNLVFFSIVLLFVVFELEVLIFII

*D. immitis* LLMYFFSMFVSYKDFFESKVSSYECGFDVCKKVHVGFNLVFFSIVLLFVVFELEVIIFVF

*N. americanus* LLLYLLNFYLSVKLTWFSKISAFESGFLSVGKIQNSFSIHFFIMMLMFVIFDLEIVMFLG

*C. oncophora* VLLYVLSFVISLKKSELLKVSTFESGFVSLSKVQNSFSIHFFVIMLMFVIFDLEIVMFLG

*A. duodenale* AVLYLLNFFLSIKKSDLLKVNAFESGFISIGKIQNSFSIHFFIMMLMFVIFDLEIVMFLG

*A. caninum* LVLYLVNFFLSIKKNDLLKVNAFESGFISIGKIQNSFSIHFFIMMLMFVIFDLEIVMFLG

*B. phlebotomum* FFLYFLNFFLSIKDFNLLKVGAFESGFLSIGKIQNSFSIHFFVMMLMFVIFDLEIVMFLG

*H. contortus* FVLYILNFFISVKKVELIKINTFESGFMSVGKIQNSFSIHFFIMMLMFVIFDLEIVMFLG

*C. elegans* FAFYLINFLLSIKDMGKNKISAFECGFVSVGKIQNSFSIHFFIMMLMFVIFDLEIVMFLG

*S. carpocapsae* FLFYFINFFLSNKLLEKNKVSSFESGFVSVGKVQNSFSIHFFVIMLMFVIFDLEVVMLIG

*A. suum* FVFYIGNFVLSCKDFYKNKISSFECGFVSIGKIQNSFSIHFFIMMLMFVIFDLEVVMFLG

*A. simplex* LLFYLGNFVLSCKDFYKNKISSFECGFDSVGKIQNSFSIHFFIMMLMFVIFDLEVVMFVG

*T. canis* VIFYLGNFVLSCKDFYKNKISSFECGFVSVGKIQNSFSIHFFIMMLMFVIFDLEVVMFVG

*S. stercoralis* FFFYLFVCFASFFDFYFCKSTSFESGFMSVGLIQNSFSIHFFVIMIMFVVFDLEIVIFIG

************************************************************

2460

=========+=========+=========+=========+=========+=========+

***Agamermis sp.*** SFS---KFMMLIWIIMIFIFFSLWIEFKSFSINWTMXXXXMMSAMISLMLFML---MIMV

***T. cosgrovei*** LFK---ETKIYTLMVLSFIYFTIWLELVMKSYLWSNXXXX------MKFLYIS---LALL

***S. spiculatus*** SIKL-FSHLIIILMIYMFIMFTMLMELNLLTLKWNNXXXX------ILLFIMT---LMTM

*X. americanum* GVLSSSLVDSFWILLFSFMLVTLLLEWVLSGLKWIVXXXX---MVALFVSFMVGVALLLV

*B. malayi* LIQGDFYGIFSFFLFFLYVVFSFYMEWYFGKLLWCFXXXX------VFLFFFC---FLYF

*O. volvulus* LIQGDFYSLLSFFVFFFYVVFSFYMEWCFGKLIWFCXXXX------LFLFFFS---FLFL

*D. immitis* LVQGDLFSVFSFFMFFFYVVLSFYMEWSFGKLIWICXXXX------VFLFVLT---FLFF

*N. americanus* LLISDFSSFVSFIMLVFFIFGGFYMEWWFGKLVWIIXXXX------IIFLFIS---LFML

*C. oncophora* LVLSDFSAFVGFFMLMFFIMMGFYMEWWYGKLIWVIXXXX------IIFMFVS---LMMF

*A. duodenale* LLVSDFASFVSFLMLIIFIFGGFYMEWWYGKLVWVIXXXX------IIFLFIS---LFML

*A. caninum* LLISDFASFVSFLMLIIFIFGGFYMEWWYGKLVWVIXXXX------IIFLFIS---LFML

*B. phlebotomum* LLISDFSSFFSFLILMIFIFGGFYMEWWYGKLIWVVXXXX------IMFLFIS---LFML

*H. contortus* LLMSDLNSLISFVMLMFFIFVGFYLEWRMGKLVWIVXXXX-------IFMFVS---LLML

*C. elegans* ILVSDLSSYISFLMMFIFILGGFYMEWWYGKLVWVIXXXX------IMFLFVS---LFMF

*S. carpocapsae* LLVSDFNSFFSFIMLLFFVLGGFYMEWWYGKLIWIIXXXX-------MFLFFS---ILSF

*A. suum* ILVSDLNSLISFFMLLMFIFGGFYMEWWYGKLVWLIXXXX------IIFIFIS---FLSL

*A. simplex* ILISDISSLLSFLMLLFFILGGFYMEWWYGKLIWLIXXXX------IIFMFIS---SLSL

*T. canis* VLVSDVSSLVSFLLLMLFILGGFYMEWWYGKLVWLIXXXX------IIFMFIS---FLSL

*S. stercoralis* ILISDLNSFVCFFFLFFFLFLFFYMEWYYGKPVWNFXXXX------LFLFFIG---IFFL

**********************************--------------------------

2520

=========+=========+=========+=========+=========+=========+

***Agamermis sp.*** ---LSHYYMIIFMMIVLELLNLIFLLMILKYMN--LEIVLLSLMIQVLDSLIIMVFMIIS

***T. cosgrovei*** ---INRLNQIIFSVICLEAMNIGLVILLS-MAT--PMLLLIILSFQVMESLLVLTFLLKH

***S. spiculatus*** ---MKNYSNLIFYLITMEVMMFLVIIQLILIMSNSFYLIFMFLSIQVSDSVLLMVLFLKN

*X. americanum* SFSVFKWSKPLLVIVLLEGVVVLLLCLLFQWVA--GPVILAILSFFVGEALILLTSFFSV

*B. malayi* ---VFKYNYLIFVLLGIELLFFSLLVYYIFFFN--SIVFFYFLCFGLMSGVVGLVIFFFS

*O. volvulus* ---FFKYERLVFILLGIEFLFFSLLVYYVFLFE--SVMFFYFLCFGLMSGVLGLVIFFFC

*D. immitis* ---VFKYDRLIFVLIGIEFFFFSLLVYYVYFFE--SVLFFYFMCFGVISGVVGLVIFFFC

*N. americanus* ---FFKWYRFIFILISLEFMMMSLFIKFSGILT--EIMFFYFMCFSVISSILGMVVMVGG

*C. oncophora* ---LFKWYRFIFILIALEFLMMSLFVSFMNNIV--EMMFFYFMCFSVISSIVGMVIMVTS

*A. duodenale* ---FFKWHRFIFILISLEFMMMSLFMKFMGLLT--EIMFFYFMCFSVISSILGMVVMVGG

*A. caninum* ---FFKWHRFIFILISLEFMMMSLFMKFMGLLT--EIMFFYFMCFSVISSILGMVVMVGG

*B. phlebotomum* ---LFKWHRFIFILISLEFMMMSLFIKFMGLLT--EIMFFYFMCFSVISSILGMVVMVGG

*H. contortus* ---FFKFYRFIFILIALEFLMMSLFVKFISIVS--NMMFFYFMCFSVVSSILGMVMMVGN

*C. elegans* ---IFKWQRLIFILISLEFMMLSLFLKFSYVLG--EMMFFYFMCFSVISSILGMVVMVGN

*S. carpocapsae* ---LFKWRRLLFVLISLEFLMMSLFIIYSYMLN--EMMFFYFMCFSVISSVLGMLMMIGS

*A. suum* ---FFKWQRLMFILISLEFIVMSLFILFSGDLN--EMMFFYFMCFSVVSSVLGMVVMVGN

*A. simplex* ---FFKWQRLIFILISLEFIVMSLFIYFSTVLN--EMMFFYFMCFSVISSVLGMIVMVGN

*T. canis* ---FFKWQRLMFILVSLEFIVMSLFIYFSGVLN--EMMFFYFMCFSVVSSVLGLVLVVGN

*S. stercoralis* ---FYKFRRFIFVIISFEFLMMGVFYLFSFFFG--FFSFFYFLCFSVFCSLMGVVLMVYF

---************************--------*************************

2580

=========+=========+=========+=========+=========+=========+

***Agamermis sp.*** LFSWEKSNNWSNLLNFSVXXXX----------------------MLCKILMFSTILIVFN

***T. cosgrovei*** ISAISHNNG---MLVLQ-XXXX------------------MLNCKFNLHPELSALFTCWM

***S. spiculatus*** SFFWVNLNM---WYLL--XXXX--------------------------MIIMFLLIKTLK

*X. americanum* LRWSGSPYG--SIFFY--XXXX----------------MMFWMALFSIMVIQLNDYSLVT

*B. malayi* VKSFGVDKV---MFYF--XXXX-LYLFFFIVLLFFFSPFLFFVFFSIFVLLGFFDYSWCG

*O. volvulus* VKGFGVDKV---MFYFL-XXXXLLYLFFFVVLLFFFSPFLFFVFFMFFVLYGFFDYSWFG

*D. immitis* VKGYGFDKV---MFYFL-XXXX-LCLFFFVVLSLFFTPFLFFVFFMFFILFGFFDFSWSG

*N. americanus* MKFYGNDQC---IF----XXXX-LLEWLLLSLMMIFSPVYFFLFLLFFSFMMLKNLSWSG

*C. oncophora* VKFYGNDYC---IY----XXXX-LFEFMMVSFLFFFLPNYFFFLLLLFSVSLFNKISWNG

*A. duodenale* MKFYGSDQC---IF----XXXX-LMELLFMSLMMFFKGLYFFFFIIMFGIFLFKDLSWSG

*A. caninum* MKFYGSDQC---IF----XXXX-LMEFLLMSLMMFFKPLYFFFFMIMFSFFLFKNLSWSG

*B. phlebotomum* MKFFGNDQC---IF----XXXX-LLEYLFLSFLMFFSPIYFFLFLIIFCFLMFKNISWMG

*H. contortus* MKYYGDDKC---VF----XXXX-MIKFLIFSTMFFFVPYFFYLFMMMLMMKMLNEMSWMG

*C. elegans* MKFFGSDNC---IF----XXXX-LLEFLFISLLWLFKPIYFLLFTVMFSFLIFNNFSWGG

*S. carpocapsae* VKFYGSDNC---LF----XXXX-LMYLFLLVLFFFYNPFLFFFFFFFFSFISINIYCWGG

*A. suum* VKFYGSDLC---LF----XXXX-LLDILLFSLYFFFEPVLFFFFMVVFGFVALNNYSWLG

*A. simplex* MKFYGSDQC---LF----XXXX-LMEMLLFSLYFFFSPFMFFLFMVFFSFCVLNNYSWGG

*T. canis* MKFYGSDQC---LF----XXXX-MMELLLFSFYFFFSPWLFFMFMVVFSFFMLNSYAWGG

*S. stercoralis* IKFYGCDYV---FF----XXXXFFSFFKVFFCWFFFFMIFFLLFSFAFCFFCFSFFCWSG

*********-------------------------------********************

2640

=========+=========+=========+=========+=========+=========+

***Agamermis sp.*** LYSWFENFAILMILSVSLVWISLNFMKMYFKTSIILMNFMWMLMLIFFMTNTIWKFYILF

***T. cosgrovei*** LTSWWFMFSKLLISVLFIIWLVLKMVWGLNFNNKLYTMILGLIIVLFLVTDYLWKFYMLF

***S. spiculatus*** VYVMFKSWLNLFLLIMMVIWLLLNFLSLMTWSKNLLVNSMMIIIWLFFISNSVSLLYIIY

*X. americanum* NVGLLDSFSVFMIWLAVGSVV-LAYLSS--RSFTILWGSLMVFCISTFLMSNLFWFYLMF

*B. malayi* CFFFFDSFNFVFLSFMSVFILGFICVSEVLGGLVFYSCLIVFFSVCFFFSGSFLILYIFY

*O. volvulus* CLFFFDSFNFVFLSFMSVFVMGFICVSELLSGLVFYSCLVVFFSVCFFYSGSFLMLYVFY

*D. immitis* LFFFFDSFNFVFLSFMSVFVLGFICVSEILVGLVFYSCLVVFFSVCFFYSGSFLVLYVFY

*N. americanus* LFFVVDSNVYVLLIFMMIFIYGMVLISEKNFSLLMLSSGLILVCLMFFVSSNMLMLYMYF

*C. oncophora* MFFFVDSYVFLLLIMMMLFIYGMVMISEKNMNLLILSGVLIVICYFFFVSSNLLMLYMFF

*A. duodenale* IFFVVDSNVYVLLIFMMIFIYGMVLISEKNFSLLILSGVLIMLCLMFFVSSNMLMLYMYF

*A. caninum* LFFVVDSNVYVLLIFMMIFIYGMVMISEKNFSLLVLSGVLIMLCLMFFVSSNMLMLYMYF

*B. phlebotomum* LFFVIDSNVFVLLVFMMIFIYGVVLLSEKNFNLLILSGMLIVICLFFFVSSNILMLYMYF

*H. contortus* LFFFVDSNVYVLLIFMMMFIYGMVMISENNKNLLILSGVLIFICLFFFVSTNMMMLYVFF

*C. elegans* LFLVLDSYSFILLIVMSLFILGIIVISEKNNNLLILSEILVFICIIFFIPSNMMMLYMFF

*S. carpocapsae* LFFFFDSFVFSLLILMSLFILGVVIMSEKNFNLIILSEILVVMCVFFFIPVNIIMLYVFF

*A. suum* CFYFFDSFSFILLIVMSLFILGVVLLSESNFMLLLLSEVLVVVCVFFFVPSNVILMYMYF

*A. simplex* LFFFLDSFTFVLLVIMSLFILGVVVLSEKNHMLLLLSEILVFVCIFFFVPVNIIMFYMFF

*T. canis* CFFFLDSFSFILLVVMSLFILGIVLLSEKNNSLLVLSELLVFVCVFFFVPVNVIMMYVFF

*S. stercoralis* NFFLIDSYFFMLLFVLCLFIFSLVLVSERHRVIVFFSKVLLVISFFFFFPYSVFYLYVFF

************************************************************

2700

=========+=========+=========+=========+=========+=========+

***Agamermis sp.*** EMNMIPMVLIIMSWGSNMARINSSIYMMLYTFFFSLPVLVIIMNNLKFMGFSSLDLNNHY

***T. cosgrovei*** ELSMIPMLVLILGYGINLSRLMASFYMLMYMLIFSFPMLSVILIN--MCLSTNLLVENWN

***S. spiculatus*** ESIMLLMILLIVGWGLNPYKFNAFMYLMAYSMLFSLPAMMVVLVNYQMLLTYNLMAKNLN

*X. americanum* ELSLLPIFVMILFWGGQPERLSASMYFIVYTIVFSVPLLVMIMG--IGIVYFWWPLY-SM

*B. malayi* ELTMLPVLFCLLGYGRQVEKISACYYLVFYTLFFGMPFLFFFSHVFNFFNFVYYD---FF

*O. volvulus* ELTMVPILFCLLGYGRQVEKISACYYLIFYTLFFGMPYLFLYSHVFFFLNFVYYD---FF

*D. immitis* ELTMIPMLFCLLGYGRQVEKISACYYLIFYTLFFGMPYLFLYSHVFFFMNFVYYD---FF

*N. americanus* ELSMFPILVMILGYGSQIEKVNSGYYLLFYAAICSFPFLFIYYKSFFLFSMCYFD---FV

*C. oncophora* ELSLFPILVMILGFGSQIEKINSGYYLLFYASLCSFPFLYVYYKSNFMLSLCYFD---FF

*A. duodenale* ELSMFPILIMILGYGSQIEKINSGYYLLFYAAVCSFPFLFIYYKSMFMFSFCYFD---FV

*A. caninum* ELSMFPILIMILGYGSQIEKINSGYYLLFYAAICSFPFLFIYYKSMFMFTFCYFD---FV

*B. phlebotomum* ELSLFPILVMILGYGSQIEKVNSGYYLLFYASFCSFPFLFIYYKSLFLFSYCYFD---FF

*H. contortus* EMSMFPILVMILGFGQQIEKINSAYYLMFYASFCSFPFLFIYFKSNLMLSFCYFD---FL

*C. elegans* ELSMFPILVMILGYGSQIEKINSSYYLMFYAAFCSFPFLFVYFKSNFLLVFTYYN---FV

*S. carpocapsae* EFSMFPILVMILGYGSQIEKISSSYYLIFYASLCSFPFLFVYFSSDLSFMLVYFD---FF

*A. suum* ELSMFPILVMILGYGSQIEKINSSYYLIFYAALCSFPFLFVYFKSFFFISLVYFD---FN

*A. simplex* ELSMFPILVMILGYGSQIEKINSAYYLIFYAAFCSMPFLFVYFKSYFFFSWVYFD---FN

*T. canis* ELSMVPILIMILGYGSQIEKINSAYYLIFYAAFCSFPFLFVYFKSFFFISLVYFD---FN

*S. stercoralis* ELSMVPIIILILGYGYQIEKVNSFFYLVFYATFCSVPFLFVYFSLDFFFVVPYFD---CF

*******************************************************---**

2760

=========+=========+=========+=========+=========+=========+

***Agamermis sp.*** LNNIFNFMLILMFMVKIPVFGLHYWLPKAHVEASTMGSMILASGLLKTGSFGFFKTLLWN

***T. cosgrovei*** LNIFTSYFTVMPFLTKCPMFGLHLWLPKAHVEASTTGSMILAAGLLKMGTYGLMKLMFWN

***S. spiculatus*** VSVLSFIMLMMMFLVKMPVFFLHYWLPKAHVEASTTGSIILASGLLKLGSVGVFKIMNWG

*X. americanum* PSLLMSITFTLPFLVKLPVFGLHFWLPKAHVEASTSGSMILASLLLKLGSFGLFRVLSMM

*B. malayi* MSFELVFLLSLCFLVKFPVYFLHAWLPKVHVESPTSTSMVLAGVMLKLGGAGIYRISKSL

*O. volvulus* VSYEFIFLLSLCFLVKFPVYFFHVWLPKVHVEAPTSASMILAGVMLKLGGAGVYRISKSL

*D. immitis* FSYEFIFLLSLCFLVKFPVYFLHVWLPKVHVEAPTSSSMILAGVMLKLGGAGVYRISKSF

*N. americanus* ISWELFFILTLSFMMKFPVYFLHLWLPKAHVEAPTTASMLLAGLLLKLGTLGYLRILGSM

*C. oncophora* MNWELFFVLTLSFMMKFPVYFLHLWLPKAHVEAPTTASMLLAGLLLKLGTAGFLRIMNSM

*A. duodenale* ISWELFFILSLSFMMKFPVYFLHLWLPKAHVEAPTTASMLLAGLLLKLGTAGYLRILSSM

*A. caninum* ISWELFFILSLSFMMKFPVYFLHLWLPKAHVEAPTTASMLLAGLLLKLGTAGYLRILSSM

*B. phlebotomum* ISWGMFFILSLSFMMKFPVYFLHLWLPKAHVEAPTTASMLLAGLLLKLGTAGFLRVLGSM

*H. contortus* MSWEMFFILTLSFMMKFPVYFLHLWLPKAHVEAPTTASMLLAGLLLKLGTAGFLRVMKSM

*C. elegans* ISWEMFFILSLSFMMKFPIYFLHLWLPKAHVEAPTTASMLLAGLLLKLGTAGFLRILGSL

*S. carpocapsae* LSWEVVLILSLSFMMKFPVYFLHLWLPKARVEAPTTASMLLAGLLLKLGTAGFMRILGSL

*A. suum* LSWEMVFVLSLSFMMKFPVYFLHLWLPKAHVEAPTTASMLLAGLLLKLGTAGFLRILGCL

*A. simplex* LSWEMVFILSLSFMMKFPVYFLHLWLPKAHVEAPTTASMLLAGLLLKLGTAGFLRILGCF

*T. canis* LSWEMVFVLSLSFMMKFPVYFLHLWLPKAHVEAPTTASMLLAGLLLKLGTAGFLRILGSL

*S. stercoralis* ISWEMVFIISLAFMMKFPVFFLHLWLPKAHVEAPTSASILLAGLLLKFGTVGFVRIMKSL

************************************************************

2820

=========+=========+=========+=========+=========+=========+

***Agamermis sp.*** --SAIL-NYSWMLIGFVFSSLYCCMQSDQKKLIALSSVSHMSLACCSLLSFSNIGMFGMI

***T. cosgrovei*** --AWTI-NNTWLLLGMVLSVYLCSLQSDFKKLVAYSSVAHMSMSII-ILNLSIISFISMV

***S. spiculatus*** NFIKLS-NYSFFVVGTFVMSLCCLFQTDFKKLIALTSVVHMNMSLTSIFYSSVSGLKSFT

*X. americanum* VLS---VFPILMFFCAIISSIFTIMQVDFKKLVAYSSVTHMTFLSVASMTVNKTILFVMV

*B. malayi* NFFGFELLVFFSLIGMVFCSFICIFQSDCKSLAAYSSICHMGFVLLSEISMLYYGKSMAL

*O. volvulus* NFFGFEMLIFFSLISMVFCSFICVVQSDCKSLAAYSSVCHMGFVLLSEISMVYYGKSMAL

*D. immitis* NYYNFEFLIFFSLVSMIFCSFICMVQSDCKSLAAYSSICHMGFVLLSELSMVYYGKSMAL

*N. americanus* NFVYNNFWIIISLLGMILASVSCTFQSDSKSLAAYSSVTHMSFLLLSMVYIFMSSKLGSL

*C. oncophora* SFIYNNFWILISLLGMILASICCVFQSDSKSLAAYSSVTHMSFLLLSLSLLLISGKVAGL

*A. duodenale* NFVYNNFWIIISLLGMILASFSCVFQSDSKSLAAYSSVTHMSFLLLSMIYIMMSSKIGSL

*A. caninum* NFVYNNFWIIISLLGMILASFSCVFQSDAKSLAAYSSVTHMSFLLLSMIYIMMSSKIGSL

*B. phlebotomum* NFVHNNFWVILSFLGMILASVCCTFQSDSKSLAAYSSITHMSFLLLSLVLVFMSSKLSGL

*H. contortus* NFVYNNVWIIIALLGMILSSFICVFQSDSKSLAAYSSVTHMSFLLLSLIFMMMSSKNSAL

*C. elegans* SFVHNNVWILIAFLGMILGSFCCVFQSDSKALAAYSSVTHMSFLLLSLVFITMSSKISSV

*S. carpocapsae* NFIHLNFWFILAFLGMILASFCCIFQSDSKSLAAYSSVTHMSFLLLSFLFMFMSSKTSSL

*A. suum* SFVHNNVWIVLAFLGMILASFCCMFQSDAKALAAYSSITHMSFVLMALVFIIMSGKTGGV

*A. simplex* SFTHNNVWMILAFLGMILSAFCCIFQSDAKALAAYSSITHMSFLLMALVFVLMAGKTSGL

*T. canis* CFVHSNVWVLLAFLGMILAAFCCVFQSDAKALAAYSSITHMSFLLMAIVFVMMSGKTSGI

*S. stercoralis* SYCHINYWFFFSVLGMFISCFVCVFQSDFKSLAAYSSVVHMNFLLFFLLFFGLYSKTGSF

************************************************************

2880

=========+=========+=========+=========+=========+=========+

***Agamermis sp.*** ILNFTHSIISSFLFLNSGIFSSFSKMRLFKYLPK--TSFHN-IILLVVSIILNLGLPPAL

***T. cosgrovei*** MINLSHTLISSCMFYFSGLMSALVKSRLIFFMST--TILTQ--YILFCLMLMNLSVPPMV

***S. spiculatus*** MINVIHSISSFMLFYLAGMLMVFSKTRLVYLQMM--LKFSLLFYLFMCAIFMNLSVPPFF

*X. americanum* LFSVAHGWVSSSLFFLVGQSSSVSYSRLGVLLS-SSSNLFWFYLMFGLILMSNSSIPPMP

*B. malayi* IMMLSHGYTSVLMFYFIGEFYHIASSRLVYYLRGYFSINLLFCLIFCLTMISNFGFPSSI

*O. volvulus* VMMLAHGYTSVLMFYFIGEFYHIANSRLIYYLRGYFNVSMLFCLMFCLTMVSNFSFPVSV

*D. immitis* VMMLSHGYTSVLMFYFIGEFYHIANSRLVYYLRGFFCVSMLFCLMFSLTMLSNFGFPSSI

*N. americanus* LLMLAHGYTSTLMFYLIGEFYHVSSTRMIYFMNSFFSSSMLFGLVFSLVFLSNSGVPPSL

*C. oncophora* MMMLAHGYTSTLMFYVIGEFYHVSSSRMIYFMNNFMSSSMMISILFSLIFLSNSGVPPSL

*A. duodenale* MMMLAHGYTSTLMFYLIGEFYHTSSTRMIYFMNSFFGSSMFLVFIFSLVFLSNSGVPPSL

*A. caninum* MMMLAHGYTSTLMFYLIGEFYHTSSTRMIYFMNSFFGSSMFLEXIFSLVFLSNSGVPPSL

*B. phlebotomum* MMMLAHGYTSTLMFYLIGEFYHISSTRMIYFMNSFFSSSMFFGLIFSLVFLSNCGVPPSL

*H. contortus* MMMLAHGYTSTLMFYVIGEFYHTSSTRMIYFMNSFMNSSMIFSIMFAMIFLSNSGMPPSL

*C. elegans* MLMLAHGYTSTLMFYLIGEFYHTSGSRMIYFMSSFFSSSMIMGILFSVVFLSNSGVPPSL

*S. carpocapsae* MLMLSHGYTSTIMFYLIGEFYHSSGSRMIYYMNSFFNSSIFMVVVFSFVFLSNSGVPPSL

*A. suum* ILMLAHGYTSTLMFYLVGEFYHVSGSRMVYYMSSFFGSGMIMALLFAVVFLSNMGTPPSL

*A. simplex* IMMLAHGYTSTLMFYLIGEFYHVSGSRMVYYMNGFFSSSMIMAIIFSVVFLSNSGTPPSL

*T. canis* VMMLAHGYTSTLMFYLVGEFYHVSMSRMVYYMNGFFGSSMIMALIFVVVFLSNSGTPPSL

*S. stercoralis* LMMVSHGYTSSVMFYFIGEFYRSLGSRMIYFLNSLMSSSFIFSFLMSLVFLSNCGMPPSI

************************************************************

2940

=========+=========+=========+=========+=========+=========+

***Agamermis sp.*** SFISEITCMAGVFMNNLLSAIMIFIAMILSLFFSYIYIFFSNQSFISNCFYINNKLLFMF

***T. cosgrovei*** SFISEYYTISSIFIKSHLSVLTLLLFGLISLMYTSMLYNFMNFNYN-KMKQISITSLSLI

***S. spiculatus*** SFMGELIYYSLVSVSNYIYLCLNLMILVISLMFS--LLLVNNMSMKKIVKPMLSSLMCLV

*X. americanum* SFFPEVLTICGALMSISVAVVCFALFSLLVCYFNSILFLGVSKSKGHHLKGGMLNMSEAI

*B. malayi* SFFSEYLMLNWFSSVFYFSVFFLFIYYLVSFYYSIYVMVCFMVGNKVSYVCDSRAVVCLP

*O. volvulus* SFFSEYLMLNFFSSVFYVGFLFLFFYYLVSFYYSVYILVCFLVGGKVSYVCDGRSVVCLP

*D. immitis* TFFSEYLMFNWFSSIFYISVLFFFFYYLLSFYYSIYVLICFFVGNKFSYVFDGRGIVCLP

*N. americanus* SFLSEFIVVVNSVIVSKLFFFMIFLYFMISFYYSLFLIVCGLMGKMFINVNNCNIGFSMS

*C. oncophora* SFLSEFMIVINNYMMSKLLFFFIFLYFMVAFYYSLFLIVAAFAGKYMLNFNNNNYGLSLF

*A. duodenale* SFLSEFLVVVNSIMVSKMFFFMIFVYFMISFYYSLFLIVCSLMGKMFININNFNIGFSMA

*A. caninum* SFLSEFLVVVNSIMVSKLFFFMIFIYFMISFYYSLFLIVCSLMGKMFINLNNFNAGFSMA

*B. phlebotomum* SFLSEFMIVVNSIMISKMFFFMIFIYFLISFYYSLFLIVSSIMGKFFFNISNFNVGFSFS

*H. contortus* SFLSEFIIITNSMMLNKILFFFVFVYFMISFYYSLFLIVNSFAGKVYINYNNNNFGIMMF

*C. elegans* SFLSEFLVISNSMLISKSMFVMIFIYFVVSFYYSLFLITSSLMGKGYHNFNTWNVGFSAP

*S. carpocapsae* SFVSEFITISSGISFFFFSFILVFIYFVFSFYYSIYLITNCLMGKNLIFFNTWGLGFSIP

*A. suum* SFLSEFIVISSSLNMMKFSFWVLFVYFFSAFYYSIYLLTSSVMGKGYVNFSIWNVGFSVP

*A. simplex* SFMSEFVAITSALNMMKFSFWLLFAYFFVGFYYSIYLLTSAVMGKAFINMSIWNVGFSVP

*T. canis* SFISEFSAISVSMSLFKFSFWVLFMYFFVAFYYSIYLLTSSVMGKSFVDMSVWNVGFAIP

*S. stercoralis* SFFSEFFSFSGFFSLFYNVFFLVFFYFFVSFYYCVYIIVISFLGKKFFFFKSWFFFWSLP

*****------------------*************************************

3000

=========+=========+=========+=========+=========+=========+

***Agamermis sp.*** LIHFTLMMFWFSIF---------------XXXX-----IICNLKKKNNLKLLQYLMNFLL

***T. cosgrovei*** FWHFCSMMYWLVLYYSEGLLTFFKNGGLKXXXX----------------------MFMPL

***S. spiculatus*** LPSVMGVMLWLN-----------------XXXX----------MITTNAIIKNKYFNFSF

*X. americanum* IILIQVGLSVVSIMLIFRF----------XXXXMIWNYLLACLVMNFKTWFYYIWFLLCC

*B. malayi* LIFMVYNFFWFIFVI--------------XXXX-------------FLLFVWYILIFYFF

*O. volvulus* LVFMMYNFFWFIFVI--------------XXXX-------------LLLFVWYVLIFYFF

*D. immitis* VMFMMYNFFWFIFVI--------------XXXX-------------LLLFVWYILFFYFF

*N. americanus* MVVMMFNIFWISMLY--------------XXXX-------------INILIFLMGLIFLL

*C. oncophora* MIVMMYNIFWLTMFF--------------XXXX-------------INILIYLIVMIFFF

*A. duodenale* LVLMMFNIFWLTMFY--------------XXXX-------------INILIFLMSIAFLV

*A. caninum* LVLMMFNVFWLTMFY--------------XXXX-------------INILIFLMSVVFVI

*B. phlebotomum* VVLMMFNVFWFSLFY--------------XXXX-------------INILIFLMLLVFLF

*H. contortus* LMVMMYNIFWLSYFT--------------XXXX---------------------------

*C. elegans* LVLMMYNVFWLSVFY--------------XXXX-------------INISIFLIGFVFFM

*S. carpocapsae* LIFMMYNIFWITLFI--------------XXXX-------------FSICVYLIFFCFFF

*A. suum* LVFMMYNIFWMSVFF--------------XXXX-------------IDISIFLMVFLLFC

*A. simplex* LVLMMYNILWLSIFF--------------XXXX-------------IDISIFLMVLLLFG

*T. canis* LVVMMYNIFWMSVFF--------------XXXX-------------MDISVFLMVFLLFV

*S. stercoralis* LIFFCFNVFGFFLFF--------------XXXX-------------IFFCCFLFLFFVLF

**********--------------------------------------------------

3060

=========+=========+=========+=========+=========+=========+

***Agamermis sp.*** MVIPFTNMNLMWSFINKNS-----YLSVYFCVLNLIFLISLWNIFYNVLQFSKNYMK-IL

***T. cosgrovei*** MVVEI--------YLNAEV-----YCSFLTSISSLIFLYILSMIFSKIMKFAKSYLP-SI

***S. spiculatus*** FVPYININKEIIKLEFFFN-----DLSILNNWQSLIFCVSLNLIFLMIWKFSLFYMS-SS

*X. americanum* LLIPVTSLPFKIVVTDFISNVGMFSLSILGNMQVFVFLVLVIVISLCVFSWSRFYMH-NK

*B. malayi* LIFLILLLP-YGKWIYVCGFSDFFSFVFTYNFEICLFFFVLLLVSFMVFVYGSFYMQGAS

*O. volvulus* LVVIFLFVP-YGKWSYSFGFSDYFNFTFIYNFEVCLFFLVLLLVSFMVFVYGSFYMVGVS

*D. immitis* LFSVIFFLP-YGKWVFNMGFNDFFSFILVFNFEICLLIFVLLLVSFMVFIYGSFYMLGVS

*N. americanus* LMLVILFIP-VMKLGLMLLEWDFLSFKFSFYFNSLLFSIILGLVTLSVLIFSTYYLDGEL

*C. oncophora* IVLMILFLA-EMKLSISFVEWDFFSLKLNLYFNSLLFSLILGIVTLSVLVFSTYYLDGEI

*A. duodenale* LLVMILFVP-FFKLGLLFLEWDFLSLKFNFYFNSILFSLILGLVTLSVLVFSTYYLDSEL

*A. caninum* LLMMILFVP-FFKLGLVFLEWDFLSLKFNFYFNSILFSLILGLVTLSVLVFSTYYLDSEL

*B. phlebotomum* LLFFIMFMP-FMKLGLFFFEWDFLSLKFSFYFNSVLFSFVLGLVTLSVLIFSTYYLSGEL

*H. contortus* -----------MKINFMMLEWEFLSFKMNIYFNSVLFSLILAVVTLTVLIFSTYYLNNEL

*C. elegans* GGISVWLMP-TFKLGIFFLEWDFLSLKFNFYFNSILFSFILFLVTFSVLVFSTYYLNSEL

*S. carpocapsae* FLMFFLMFF-NFKLSFFFFDWSFLSLKFNFYFNSILFSFILLVVTLSVLVFSTYYLDGEL

*A. suum* VSLFLIFFVSCVKLSFFFVEWDFLSFKISVYFNSIMFSLILLLVTISVLVFSTYYLSGEL

*A. simplex* VCPFIMLFP-ISKIAFFLVEWDFLSFKISVYFNSIMFSLILLLVTMSVLVFSTYYLDGEL

*T. canis* VCLVLILAP-FFKLSLSLVEWDFLSFKVSVYFNSVMFSLILLLVTLSVLVFSTYYLDGEL

*S. stercoralis* FVLFLFFVP-FFKFSFFFLILVLLFFKFNFYLNSIIFSFVLILISVSVFFYSLYYLNMEI

------------************************************************

3120

=========+=========+=========+=========+=========+=========+

***Agamermis sp.*** N-TYFNFMMFMFLLSMI-TLIVCNSWLTLFFGWELLGLTSFFLILYYNNWNSMSGSFLTV

***T. cosgrovei*** S-TEFLMLMVAFYTSMV-LMIMSNSWMVIFLGWEGLGVTSYCLVLYWKNWNSVTGSYLTL

***S. spiculatus*** SFKMFVMMLSLFYWSMV-LLIVSSSLFSLFLAWEGLGITSYLLILFYYNWNSIQSSNLTF

*X. americanum* SLSWFFSTLFIFVLSMI-MLIFSESLFFIFLGWEGLGVSSFLLIIFYQNWMSVNGGLLTL

*B. malayi* RLFYFFFFLFLFVLSMGGLIVFSGSIILVLIFWDFLGVSSFFLVLFYNNVVSRSSSMSTV

*O. volvulus* RLFYFFFFLFLFVLSMGGLIVFSGSIVLTLVFWDFLGVSSFFLVLFYGNVSARSGAMSTV

*D. immitis* RLFYFFFILFLFVFSMCGLIVFSGSVVLTLIFWDFLGVSSFFLVLFYGNVGSRNGAMSTV

*N. americanus* NFNYYYFVLLVFVGSMF-MLNYSSSIFIMLLSWDLLGISSFFLVLFYNNWDSNSGAMNTA

*C. oncophora* NFNYYYFVLLIFVGSMF-MLNYSNNVINMLISWDLLGISSFFLVLFYNNWDSNSGAMNTA

*A. duodenale* NFNYYYFVLLIFVGSMF-MLNYSSSIFTMLLSWDLLGIYSFFLVLFYNNWDSNSGAMNTA

*A. caninum* NFNYYYFVLLIFVGSMF-MLNYSSSIFTMLLSWDLLGISSFFLVLFYNNWDSNSGAMNTA

*B. phlebotomum* NFNYYYFVLLIFVGSMF-MLNYSSSIFTMMLSWDLLGISSFFLVLFYNNWDSSSGAMNTA

*H. contortus* NFNYYYFVLLIFVGSMF-MLNFSNNVLAMLMSWDLLGISSFFLVLFYNNWDSNSGAMNTA

*C. elegans* NFNYYYFVLLIFVGSMF-SLNFSNSIFTMLLSWDLLGISSFFLVLFYNNWDSCSGAMNTA

*S. carpocapsae* NFNYYYFVLLIFVGSMF-SLNFSNSVFTMLISWDILGISSFFLVLFYNNWDSCSGAMNTV

*A. suum* NFNYYYFMLLVFVGSMF-SLIFSSGCFSMLVSWDLLGISSFFLVLFYNNWDSCSGAMNTV

*A. simplex* NFNYYYFVLLIFVGSMF-SLIYSNNCFSMLLSWDILGISSFFLVLFYNNWDSCSGAMNTV

*T. canis* NFNYYYFMLLIFVGSMF-SLIYSSSSFTMLFSWDLLGISSF-LVLFYNNWDSCSGAMNTV

*S. stercoralis* NFFYYFFVLLVFICSMF-FLVFCDSVFFVLLGWDLLGISSFFLVLFYNNWDSCSGSMNTV

*****************--*****************************************

3180

=========+=========+=========+=========+=========+=========+

***Agamermis sp.*** MSNRLGDMFFILSL--------YFFMSFKYSTSMMIMLSMMCFTKSAQFPFSTWLPAAMA

***T. cosgrovei*** MTNRVGDAFLLMSF--------FVLSMKIKSVQFFLLLLVLMMTKSCQVPFSGWLPAAMA

***S. spiculatus*** ISNRFGDSFMLIML--------FLS----ESFLLLLVIFVMLLTKSAQVPFSSWLPAAMA

*X. americanum* LTNRVGDACLIISFS---YWMFLLSPTFNLNQFLIILVFILAFTKSAQWPFTSWLPAAMA

*B. malayi* FTNRIGDFCIFLFFNGFIFFSVGVFSYQFFSSLLVFMLFISAFVKGGQYPFGSWLPKAMA

*O. volvulus* FTNRIGDFCIFLFFNGFVLFSMSFLSYQFFGSLLVFMLFVSSIIKGGQYPFGSWLPKAMA

*D. immitis* FTNRIGDFCIFFFFNGFVLFSLGHFSYQFFSSLMIFMLFLSSFIKGGQYPFGSWLPKAMA

*N. americanus* LTNRIGDFFIFSFFSGSLFFGYYFLGFELLCGSMVLLLLLTSFTKSAQFPFSSWLPKAMS

*C. oncophora* LTNRVGDFFMFTFFSGSLFYGYYFLSLEFLCGFSVILLLIASFTKSAQFPFSSWLPKAMS

*A. duodenale* LTNRIGDFFIFSFFSGAIFYGYYFLSFEMMCSSMVLLLLLTSFTKSAQFPFSSWLPKAMS

*A. caninum* LTNRIGDFFIFSFFSGAIFYGYYFLSFEMMCSSMILLLLLTSFTKSAQFPFSSWLPKAMS

*B. phlebotomum* LTNRIGDFFIFCFFSGGIFYGYYFFSFELVCSFMVLMLLLTSFTKSAQFPFSSWLPKAMS

*H. contortus* LTNRLGDFFLFTFFSGCVFVGFYFISFEFFSLSMMILLLLTSFTKSAQFPFSSWLPKAMS

*C. elegans* LTNRLGDYFMFVFFGLSVFSGYYFLSFSMFSSYMSLLLLLTAFTKSAQFPFSSWLPKAMS

*S. carpocapsae* LTNRLGDFFIFFFFSFSFFSSFYFLSLSFFVCFSSLMLILTAFTKSAQFPFSGWLPKAMS

*A. suum* LTNRLGDFFLFVFFSSTIFSSYYFLSLSFFCWLSSLMLLLASFTKSAQFPFSGWLPKAMS

*A. simplex* LTNRLGDFFLFVFFSSVLYSSYYFLNLSLFMGLSSLMLLLASFTKSAQFPFSGWLPKAMS

*T. canis* LTNRLGDFFLFIFFTSVMFSSYYFLSLSWFCGVSSLMLLLASFTKSAQFPFSGWLPKAMS

*S. stercoralis* LTNRMGDFFIFVFFSLSFFTFFNFFSFSYFCFYSSFFLVLAAFTKSAQFPFGGWLPKAMS

************************************************************

3240

=========+=========+=========+=========+=========+=========+

***Agamermis sp.*** APTPVSSLVHSSTLVTAGIYLYYRFNLMLFNLN--KIFVMLFLITTFIGSLSALIETDLK

***T. cosgrovei*** APTPVSALVHSSTLVTAGVYIVYLFEFPFNSLGGSKIFLVIFLLTMAMGSWSALLEVDMK

***S. spiculatus*** APTPVSALVHSSTLVTAGIILSMKYKL-YEVMNVSKLMFLLGLLTAITGSASALNSMDIK

*X. americanum* APTPVSALVHSSTLVTAGIWMLIRFFY----NSSSILWLLFGSLTTLMASLAALLEADTK

*B. malayi* APTPVSCLVHSSTLVTAGVMLMDCYFYVCLNSDVLTFVFYVGFFTMVFSGLCALVEQDAK

*O. volvulus* APTPVSCLVHSSTLVTAGVMLMDCYVYVSLNSDVLSFVFYVGFFTMVFSGFCALVEEDAK

*D. immitis* APTPVSCLVHSSTLVTAGVMLMDCYVYVSMSADVLSFVFYVGFFTMLVSGVCSLFESDVK

*N. americanus* APTPVSSLVHSSTLVTAGLILLMNFSKLMLNGSVMMLVLLVGMFTMFFSSISALVEEDMK

*C. oncophora* APTPVSSLVHSSTLVTAGLILLMNFSNLLLNNLVLMIMLLIGVFTMFFSSITALVEEDMK

*A. duodenale* APTPVSSLVHSSTLVTAGLILLMNFSKIMLNGSVMMVILLIGLFTMFFN-VTALVEEDMK

*A. caninum* APTPVSSLVHSSTLVTAGLILLMNFSKIMLNGSVMMIILLIGLFTMFFSSVAALVEEDMK

*B. phlebotomum* APTPVSSLVHSSTLVTAGLILLMNFGSLMLNSLVMMVVLLIGLFTMFFSSIAALVEEDMK

*H. contortus* APTPVSSLVHSSTLVTAGLILLMNFWYFSFNNVFMNVILIVGSVTMFFSSVTALVEEDMK

*C. elegans* APTPVSSLVHSSTLVTAGLILLMNFNNLVMQKDFISFVLIIGLFTMFFSSLASLVEEDLK

*S. carpocapsae* APTPVSSLVHSSTLVTAGLILIMNFSEIVFNINISQIILLIGIFTMFFSSITALVEEDMK

*A. suum* APTPISSLVHSSTLVTAGLVLIMNFSEMILNKDVIMIIMVVGVFTMFFSSMAALVEEDLK

*A. simplex* APTPISSLVHSSTLVTAGLVLVMNFTELILHKYVISIILVIGVFTMFFSSMAALVEEDLK

*T. canis* APTPVSSLVHSSTLVTAGLVLVMNFSEMVFNKNVLGVVLVIGIFTMFFSSMAALVEEDSK

*S. stercoralis* APTPVSSLVHSSTLVTAGIILMIKFNVIIMSSFLLKLVLISGVFTMFFSSFCALYENDLK

************************************************************

3300

=========+=========+=========+=========+=========+=========+

***Agamermis sp.*** KLVAFSTLSQLGLIFMSFFSSIHGLMFYHLLVHAFFKSILFIISGLMIWSMNSNQ---NL

***T. cosgrovei*** KIVAFSTLSQLGLISLGFYSLISGMLILHLLTHAIFKSLMFMIMGMLIMSMYGSQNTFNF

***S. spiculatus*** KMVAFSTLSQLGFMVMSFYSSMVTFVLGHLLIHAFLKSLLFLMVGLNMMAMSSQQ--LTF

*X. americanum* KIVALSTLSQLGMMVLSLYLGGKLICFFHLVCHALAKANLFLIVGSMLSLNYSQQ---DS

*B. malayi* KVVALSTMSQIGFCFLAIGSGLHYISFIHMISHSFFKSLLFMQIGYLIYINCGNQDYRGY

*O. volvulus* KIVALSTMSQIGFCFLAIGSGLHYLSYVHMISHSFFKSLLFMQMGYLIFINFGQQDYRGY

*D. immitis* KVIALSTMSQIGFCFLAIGSGLHYLSYVHMISHSLFKSLLFMQVGYVIYINLGQQDYRGY

*N. americanus* KVVALSTLSQMGFSMVTLGLGMSFVSFLHLVSHALFKSCLFMQVGYVIHSNFGQQDGRGY

*C. oncophora* KVVALSTLSQMGFSMLTLGLGLSFISFVHLVSHALFKSCLFMQVGYLIHCNXGQQDGRSY

*A. duodenale* KVVALSTLSQMGFSMVTLGLGMSFVSFIHLVSHALFKSCLFMQVGYIIHCNYGQQDGRGY

*A. caninum* KVVALSTLSQMGFSMVTLGLGMSFVSFIHLVSHALFKSCLFMQVGYIIHCNYGQQDGRGY

*B. phlebotomum* KVVALSTLSQMGFSMVTLGLGLSFISFIHLVSHALFKSCLFMQIGYVIHCNYGQQDGRGY

*H. contortus* KVVALSTLSQMGFSMMTLGLGLSFVSLVHLMSHALFKSCLFMQVGYVIHSSFGQQDGRNY

*C. elegans* KVVALSTLSQMGFSMVTLGLGLSFISFIHLVSHALFKSCLFMQVGYIIHCSFGQQDGRNY

*S. carpocapsae* KVVALSTLSQMGFSMLTIGLGLSFVSFTHLVSHALFKSCLFMQVGYMIHCSFGQQDGRGY

*A. suum* KVVALSTLSQMGFSMLTVGIGLSFVSFIHLLSHALFKSCLFMQVGYLIHCSLGQQDGRNY

*A. simplex* KVVALSTLSQMGFSMFTVGLGLSFVSFIHLLSHALFKSCLFMQVGYLIHCSFGQQDGRNY

*T. canis* KVVALSTLSQMGFSMFTVGLGLSFVSFIHLLSHALFKSCLFMQVGYLIHCSLGQQDGRNY

*S. stercoralis* KVVALSTLSQMGLSMVSFGFGLSFLCYVHLLSHAIFKSCLFMQIGYLIHCSFGQQEGSGY

************************************************************

3360

=========+=========+=========+=========+=========+=========+

***Agamermis sp.*** NKASIFN-VMIFSLMLVSILNMMSFTMTSGFLSKEILISMSLSSSMMFNIIMLVILSFTI

***T. cosgrovei*** SSFHLYN-YVSLFLIILSLLNMMSVMTTSGFFSKETLLLLMNLNKTLIIILMIFVLSFTF

***S. spiculatus*** SSSMFLK-FKYGSMYLIIMINFSSFYLTSFYLSKEMNVLALTVMNLFLLFLLMTVLMMSL

*X. americanum* RKLSVSI-NTLSMALLISILSLGGTLFQSGMYSKEQILLTHFVLSNSVYSWMILTALVTL

*B. malayi* SYLGICAPILVQLQVFVSVVCLCGLLFTSGCCSKEYFMSRFYYSSFNFFLVFFYFFGVFL

*O. volvulus* SFFGFCAPVLVQLQIFLSVFCLCGLLFTSGSCSKEYFMSRFYYDSYGFFLVFFYFFGVFL

*D. immitis* SFYNLCCPVLVQLQVFLSVVCLCGLLFTSGGCSKEYFMSRFYYDSFSFFLVFFYFLGVFL

*N. americanus* GNNGNLP-MFMQLQPLVTLFCLCGLIFSSGMMSKDLILELFFFNNYMMFFSLMFFISVFL

*C. oncophora* SYNGVFP-LFMQVQLLVTLFCLCGLIFSSGMVSKDMILELFFLNNNYLLLSLLFFVSIFL

*A. duodenale* GNNGNLP-MFMQLQLLITLFCLCGLVFSSGMVSKDAILELFFFNNYMIVFSFMFFVSVFL

*A. caninum* GNNGNLP-MFMQLQLLITLFCLCGLVFSSGMVSKDAILELFFFNNYMLMFSMMFFVSVFL

*B. phlebotomum* GGNGNLP-MLIQLQLLVTLFCLCGLIFSSGMVSKDLILEYFFFNNYILFFSLMFFISIFL

*H. contortus* GYNGMLP-LFVQLQLLVTLFCLCGLMFSSGMVSKDLVLEMFFSNSEVMIFSIMIFVSIFL

*C. elegans* SNNGNLP-NFIQLQMLVTLFCLCGLIFSSGAVSKDFILELFFSNNYMMFFSLMFFVSVFL

*S. carpocapsae* NNLGNIP-VFIQLQLLVTLFCLCGLFFSSGAVSKDFILEIFFSNSFMFFFCFMFFISVFF

*A. suum* SNLGNVP-YFIQLQLLVTLFCLCGLVFSSGAVSKDYILEFFFSNFFMVVFACMFFFSVFL

*A. simplex* SNLGNLP-SFIQLQLLVTLFCLCGLIFSSGAVSKDFILEFFFTNFFMIFFSVMFFLSVFL

*T. canis* SNLGNLP-SFIQLQLLITLFCLCGLVFSSGAVSKDFILEFFFSNFFMSGLALMFFLSVFL

*S. stercoralis* S-FGNLP-YFVQLQILITLFCLCGLFFLSGSVSKDYILTYFFSNYFMFFFSFFFFISVFI

*******--***************************************************

3420

=========+=========+=========+=========+=========+=========+

***Agamermis sp.*** IYSYRLITSFKMFLNFKMSVNFKMLG-IILTYLMFMTLFSYMWFLNWFFSFNFNVLIKSL

***T. cosgrovei*** SYSYRIIKIFLLAETSLMSNHISYNKNYLITYSVFLSFSAWLWLNNFCY-MKYDFLLTLF

***S. spiculatus*** FYAIR----FMMIISFKSNKMIKMLGYSDLGVAKIIVMTTIFMFTSLYYKNSSLLMIKPA

*X. americanum* TLSYCLK-LFFMCLWSESEKTLTTSSSVVMHLPILVLSMCTLVFGYFYNNNTFINVGTFS

*B. malayi* TFCYCYRMMYLFRVGCSFFDYVGFSSKLFYYSCFLLVFFSVVFTFWWISGMMGLVFVFNR

*O. volvulus* TFCYCYRMFFLFRVGAFGFDYVGFSSKLFYFSCFFLVFFSVVFTFWWVFSLLSFSVAFNR

*D. immitis* TFCYCYRMLFLFRVGISGLDYVGFSSKLFYGSCFFLVFFSVVFTFWWIFGLLSFSVAFNR

*N. americanus* TFGYSYRLWKSFFLSFSKVVLVYSSTFIMNFLSLLLVIFSVVFIWWLSFNMLLLPSLYLY

*C. oncophora* TFGYSYRLWKSLFVNFLKVXNNYSSSYVMNFLSLLLVIFSXVFLWWINMNLFFVPSIFLY

*A. duodenale* TFGYSYRLWKGFFLSFSKIVSIYSSTLVMNFLSLLLILFSVFFLWWLNFNMLYIPSLFLY

*A. caninum* TFGYSYRLWKGFFLSFSKIVSVYSSTLVMNFLSIVLVMFSVFFLWWLNFNMLFIPSIFLY

*B. phlebotomum* TFGYSFRLWKSFFLTFSKVVLVFSSSLVMNFLSMFLVFFSIFFLWWLNLNMMCVPCLFLY

*H. contortus* TFGYSYRLWKSLFLSFKKVIYNYSSSMVMNFLSLMLIMFSVFLLWWMNYNMFVFPTLLLY

*C. elegans* TFGYSFRLWKSFFLSFNKVMNHYSSTVFMNFLSLVLVIFSISFLWWMNFNLLNIPSLFLY

*S. carpocapsae* TFGYSYRLWKSFFLNFSSSVNNFSSGFVMNFLSFFLVFFSIVFLWWMNLNIMFLPSFFLY

*A. suum* TFGYSYRLWKGFFMSFSRPVFCFSSSVVMNFLSLLLVLFSIFFIWWMNFNMLCMPCLFLY

*A. simplex* TFGYSYRLWKGFFMSFGKPVFHYSNGVAMNFLSLVLVFCSIVFIWWLNFNMLSLPALFLY

*T. canis* TFGYSYRLWKGLFMSFSRSVYHFSSSVAMNFLSLLLVVFSIF-IWWVNFNMLTVPSLFLY

*S. stercoralis* TFLYSYRIWSSLFLTFFNSLYYSCSSFFIFFISIPLVFFSLFFIWWLNLNFFSIPVVFVY

************************************************************

3480

=========+=========+=========+=========+=========+=========+

***Agamermis sp.*** MAFMMLSMIFKSLIIN------------------------VFNNLTMFN-FFSSYVCFVN

***T. cosgrovei*** LLGLWMSLFTMFNFIS------------------------LFSTLLKLNNIFMKFFTKKY

***S. spiculatus*** IYFKVMTILFLLLVLTNHKWF-------------CYVTMFSIYKFNTINNYFSNSMMHMY

*X. americanum* SYWVLLFMFFFFASVSFPLLF-------------G----FSLQLLVSKSAFMLGFFKYIS

*B. malayi* FEFLVGFFYLFFIYCFFNYVFRYVVIEFVNKYFMDSYSFFIYKFVPNFFYFDVFILGFNY

*O. volvulus* FEFLVVYFYLFFVYCFCGYFFRYFVVEFKGKFFMDHYACFIYKIFPSFFYFDVFIMGFNY

*D. immitis* FEFLVVNFYFFFVYCFLNYFFRYFFLEFKNKFFMDSYSFIVFKIVPSFFYFDSLAMGFNY

*N. americanus* MDFYVPLFYIMLIMIFSYFVFKLLFKELVYKFLVDYLAKNVIYKLKNLKFMDNNLNKFGY

*C. oncophora* VDFYSPLYYIFMIIIFSYFVYKILFKELIYKFLVDYFAKNVIYKVKNLKFLDLWLNKIGF

*A. duodenale* LDFYAPLFYIILIVLLSYFVFKLLFKELVYKFLVDYLAKNAIYKVKNLKFMDNNLNKFGY

*A. caninum* LDFYSPLLYIVLIILLSYFVFKLLFKELAYKFLVDYLAKNIIYKVKNFKFMDNNLNKFGY

*B. phlebotomum* VDFFVPLFYLVLIFLFMYLVLNFLFKELVYKFLVDFFSKLVIYKLKNLKFVDYFINKLGY

*H. contortus* VDFYVPLLYIVIICLVSYFVYKMMFKELMYKFFVDYLAKNVIYKVKNMKFFDLNLNKLNF

*C. elegans* VDFFGPLVFLFMMIFLSFLILKMLFKELMYKFLVDYLAKNSIYKMKNLKFMDLFLNNINS

*S. carpocapsae* MDFFVPIFFLFFFVFVFFLNLKVMLMELIYKFSVDFLPKYSLLLMKSTKFSENFFTSFNS

*A. suum* VDFFVPLFFVVMIMVVGFLCVKLLLKEFVYKFLVDFFAKGWVYGLKNYKFFDLFLGGINS

*A. simplex* VDFFAPLFFLFLIIFLSFFCVKMLLKEFVYKFLVDLFAKETIYGLVNYKFFDLLLNNINS

*T. canis* VDFFVPLFFLIVMVFVAFFCVKFLLKEYVYKFLCDLFAKDIVYFLKSYKFFDLFLNNINS

*S. stercoralis* IDFYFFFFFILLFFFFYFIVFKFLSFS-FYCFLMDYFPILFTRFFYDFKFFDLFLNKLNF

***---------------------------------------------------------

3540

=========+=========+=========+=========+=========+=========+

***Agamermis sp.*** LEKDVMNSSFFFMYAFQTKMMNWKIISLAVLFMTII--------------------XXXX

***T. cosgrovei*** LDLAILDMAFYKMFVSN---MDNSLLVLSLLIFMFI--------------------XXXX

***S. spiculatus*** SDEMFSSLKYFLFKNFMLFNMNKLWLMIIVFLIMMNM-------------------XXXX

*X. americanum* SYILMLEPIYFIFSNIFLLKTTLSTLTPLTIILLVILFV-----------------XXXX

*B. malayi* FFVGLFRLFSFFFFSLFRGFYHVAVLLVFFFMVSFLFF------------------XXXX

*O. volvulus* FFFGLFRLFSFFFFSWFRGFYHVGVLIVFFFMLFFLFF------------------XXXX

*D. immitis* FFFGIARFISFFFFSLFRGYYHTGVLIIFFFMLFFLFF------------------XXXX

*N. americanus* MGFNFLGSLG-MFFTNYMVSFKYNNLIILMFFIFIFL-------------------XXXX

*C. oncophora* YGFNWIGMIS-NNFSYFMSTFKYNNVIFLIFLIFLLL-------------------XXXX

*A. duodenale* MGFNFLGGFS-TYFIGYMNSFKYNNLIILIFLLFIFL-------------------XXXX

*A. caninum* MGFNFLGGFS-TYFIGYMNSFKYNNLIILVFLLFIF--------------------XXXX

*B. phlebotomum* LIFSFLSSIS-YFFTYYLVSFKYSSLVLLVFFLFLSL-------------------XXXX

*H. contortus* VNFFAFSTFS-NVFMKYMGNFKYNNVIFLIFL------------------------XXXX

*C. elegans* KGYTLFLSSG-MFKNYYLKSLNFNSVVVLIFIFFMIC-------------------XXXX

*S. carpocapsae* KGFSFFSFLN-FFFNSSMKGLNFNSIVIVIIVLFIFF-------------------XXXX

*A. suum* LGVTFFSFTG-FWSNSYMKSLYFNSVVIVLVLFFFLVWGCILSLKYALCKRMILALXXXX

*A. simplex* KGVTFFSFSG-FLSNSYMKSLNFNSIMVVLMLFFFLI-------------------XXXX

*T. canis* KGVTFFSFQG-FWGNSYMKSLNFNSVVIVLMLIFFLIWGCILN-------------XXXX

*S. stercoralis* TFFSFFSFSSSISMSSYFNKFNFLVVLFFLFLLFLDI-------------------XXXX

------------------------------------------------------------

3600

=========+=========+=========+=========+=========+=========+

***Agamermis sp.*** ----MKIKMLIGTMIFMLLMNHPSYMCMCLAIFCLFLGFFMKSLVPNIFIYSMLIIFIGG

***T. cosgrovei*** --MKTLWFYVLIFMTIN----NPSLVVLSLMALSLNFMWFIKNMYLSLTLYMVSLTYLGG

***S. spiculatus*** --MLSFYAIMLMGMFIDYSVFMFLLMTLFMLFMCLVMKIKINLSL-----YLTMIIFLTG

*X. americanum* -MSEMLLLFTTLLMTLQSTFWCSLLTIPLSILSIFITRQVLEFSG--LVVFIFCLVFIGG

*B. malayi* YFFYFSFFLSILFFCLSFLDWDPMKSCIMMCLGILFMSCYISLGVHVWYSYFIVLIFLSG

*O. volvulus* IFFYVSVFFAFVFFCLSFLDWDPLKSCVMMCLGVMSMSCYVSLGVHVWYSYFVVLIFFSG

*D. immitis* YFFYCSFFFAFFFFCLSFLEWDSLKSCVMMCLGIVFMSCYLSFGIHVWYSYFIVLVFFSG

*N. americanus* -LYKFFLLISLFGALMSYMNMDPMKSSFFLILSMMMCMPMLSFSGYVWFSYFICLLFLSG

*C. oncophora* -ILSFFXFVPLMGSLMSYMSLDPMKSSFFLIWSMLLLMPCXSFGVNIWFSYFISXLFLSG

*A. duodenale* -MFKFFLVISLIGGVMSYVNMDPMKSSFFLILSMLMCMPMLSFSGYVWFSYFICLLFLSG

*A. caninum* -VFSFFLVVSLVGGVMSYMNMDPMKSSFFLILSMLMCMPMLSFSGYVWFSYFICLLFLSG

*B. phlebotomum* -IFKFFLLFSLLGGVLSYLNIDPMKSSFFLIMSMMMCMPLLSFMGYVWFSYFICLLFLSG

*H. contortus* -----MIMVAMLMSVLSYLSVDPMKSSFFLILTLLLSMPVLSFSGNVWFVYFVCLLFLSG

*C. elegans* -MVKVFFVLAVLSSIISYINIDPMKSSFFLIFSLLFSMPVISMSMHIWFSYFICLLFLSG

*S. carpocapsae* --LLFYFFFSVFFCFLSYINMDPMKSSFFLVLSLLLSMPLISFYFYIWFSYFICLLFLSG

*A. suum* -LLGSFFFLAIISCVMSYINVDPMKSSFFLIFSLLMVMPLISFFLHVWFSYFICLLFLSG

*A. simplex* -LVSGFFLLAILSCVMSYVNLDPMKSCFFLIFSLLMIMPLISFSLHVWFSYFICLLFLSG

*T. canis* -LLMYFFLLAIFSCALSYVNLDPMKSCFFLIFSLLMVMPLISFFLHVWFAYFICLLFLSG

*S. stercoralis* ---LFFNFFCLFFSCLSFLVFDPLKSAFFLVLSLIFLVFFISFGFYVWFSYFICLIFLSG

----------------------**************************************

3660

=========+=========+=========+=========+=========+=========+

***Agamermis sp.*** ILLLLFYLTMINTNKTSWQN----------MLYLTIFMAPDFYEIHNLKFSCSTIFIEMF

***T. cosgrovei*** ILLLLFYISMLFSNKPYWN----------IIFLLSLSMLFPCYKWHMLVFSFINANLNCN

***S. spiculatus*** IMVMMFYIILITQNYYETKN-----LKAFILLLIMLIMIKNFPIMLIFNFQLEVFDYELK

*X. americanum* LLLLIVMVSTLSHQESSLIID---KMSMIAVYMMSYFFLLKVSDIDFSNKVSAMFWYEKT

*B. malayi* IFSLLTYFCSISGCNYYYNYGYFFVVFIFFCFLFFIFFDYD-FFFFNFDYDFLSICYDFN

*O. volvulus* IFSLLTYFCSMSNFIFYYNY-FFFFSLFLVSFFFVFVVDFD-FSLFFSDFNFLYVCYDFS

*D. immitis* IFSLLTYFCSMININYYYNY-YYFFFFLFFFLFFFFFFDYDVFFFFFFDGNFLYVYYDFN

*N. americanus* IFVILVYFSSLSKISISKGY-----VVLLVFFLTLLVFGMN-FGVVVASVSLNVFYYSIF

*C. oncophora* IFVILVYFSSLSSFSMKNVS-----LSFVVLFLSLMLMLMLRYYQVKVFLSLNVFYYSLY

*A. duodenale* IFVILVYFSSLSKVNMVKGY-----LVFISLLFSLMVIGLN-YNMVLYNVSLSVFYYKIF

*A. caninum* IFVILVYFSSLSKINMVKSY-----LVVLSLIFSFFVISLN-YNMVLSNVSLSVFYYKVF

*B. phlebotomum* IFVILVYFSSLSKFSLVSGF-----LWFLAFVLSFLVLGMN-FNVNVGLINLNVFYYSIY

*H. contortus* IFVILVYFSSLSSVKYKKMY--FVNLLGFMLLMIGVMMVMLKKDLDQSMVGMNEFYYNVY

*C. elegans* IFVILVYFSSLSKINVVKSY-----MAVFLLLLSMLYFSPT-VLTYSSYLGLSGFYYSIY

*S. carpocapsae* VFVILVYFSSLSSVSNTKMP-----LSIFCFLLSFFSFFPS-YFFYYTNLSINNFYFNVY

*A. suum* IFVILVYFSSLSKIGYVVTP-----FYFVGGVLSVFFFYPF-FYSVTDVVAVNNFYFSVY

*A. simplex* IFVILVYFSSLSKVSAVGTP-----FYFLGGCLSVFFFYPF-FYGVFSVVSINNFYYSVY

*T. canis* IFVILVYFSSLSKIGSAETP-----FYTVGVILTVCLFFPF-FYGIFSSVSFNNLYYSVY

*S. stercoralis* IFVIIVYFSSFSKYIYVHIN----FFPVFFVFLFFFFYSFFFFLDPIYHNSLNFFYYDLF

*******-----------------------------------------------------

3690

=========+=========+=========+========

***Agamermis sp.*** NLKTLMIFMMGVLMVMLMIMNTFLLKVSFMRQF-XXXX

***T. cosgrovei*** --KFIVMWILLTMLGVLTMINIVLSNLSYTRQVTXXXX

***S. spiculatus*** NITMLVMLIMVIIIVVLFMISYLVMKLKLLRQLQXXXX

*X. americanum* --PLMISLPLMSLFISLMTISFFLSNSKMMSRVVXXXX

*B. malayi* --FYYVFWVVLILFILLSLISFNLGCDSFMRGL-XXXX

*O. volvulus* --YYYVFWVVFILFLFLILISFSLNGFGYMRSL-XXXX

*D. immitis* --FCYVFWIIFVLFLLLIMISFSFNGGGYMRGL-XXXX

*N. americanus* --WWLFFYLLLILLFFMNFTSYFLNFSGALRKL-XXXX

*C. oncophora* --WIVLIYVILMLLIFMNFVSYFLNFSGALRKV-XXXX

*A. duodenale* --WMIILFILFILLFFMNFTSFFLNFSGALRKV-XXXX

*A. caninum* --WMIILYILFILLFFMNFTSFFLNFSGALRKV-XXXX

*B. phlebotomum* --WLVLFFIFFVLLFFMNFTSFFLNFSGALRKV-XXXX

*H. contortus* --WLMLIFILLTLLLFMSFVSYYLNFSGALRKI-XXXX

*C. elegans* --WFIFCFILVCLLFFMNFSSYFLNFSGALRKV-XXXX

*S. carpocapsae* --WLIIFFIIMVLLVFMNFTSYYLNFSGALRKF-XXXX

*A. suum* --WMLLVWVIFVLIFFMNFTSYFLNFSGALRKV-XXXX

*A. simplex* --WSFFIWLIFILIFFMNFTSYFLNFSGALRKV-XXXX

*T. canis* --WGVLVWVILILIFFMNFTSYFLNFSGALRKL-XXXX

*S. stercoralis* --FSFIFWVVFCLFFFMNFISFFLCFSGALRLY-XXXX

------------*******************-------

Parameters used

minimum number of sequences for a conserved position: 10

minimum number of sequences for a flanking position: 16

maximum number of contiguous nonconserved positions: 8

minimum length of a block: 10

allowed gap positions: with half

use similarity matrices: yes

Flank positions of the 35 selected block(s)

Flanks: [57 110] [113 122] [125 208] [238 281] [287 368] [371 738] [785 813] [816 1006] [1037 1090] [1092 1176] [1182 1282] New number of positions in pasted_alignment-gb: 2837 (76% of the original 3698 positions)
